# The Efficacy and Mechanism of Chinese Herbal Medicines in Lowering Serum Uric Acid Levels: A Systematic Review

**DOI:** 10.3389/fphar.2020.578318

**Published:** 2021-01-25

**Authors:** Liqian Chen, Zhengmao Luo, Ming Wang, Jingru Cheng, Fei Li, Hanqi Lu, Qiuxing He, Yanting You, Xinghong Zhou, Hiu Yee Kwan, Xiaoshan Zhao, Lin Zhou

**Affiliations:** ^1^ Department of Traditional Chinese Medicine, Zhujiang Hospital of Southern Medical University, Guangzhou, China; ^2^ Syndrome Laboratory of Integrated Chinese and Western Medicine, School of Chinese Medicine, Southern Medical University, Guangzhou, China; ^3^ Department of Nephrology, General Hospital of Southern Theatre Command, PLA, Guangzhou, China; ^4^ Department of Nephrology, The First Affiliated Hospital of Zhengzhou University, Zhengzhou, China; ^5^ Department of Traditional Chinese Medicine, The Affiliated Ganzhou Hospital of Nanchang University, Ganzhou, China; ^6^ School of Chinese Medicine, Hong Kong Baptist University, Hong Kong, China; ^7^ Endocrinology Department, Nanfang Hospital, Southern Medical University, Guangzhou, China

**Keywords:** Chinese herbal medicine, serum uric acid, serum urate, efficacy, mechanism

## Abstract

*Background*. Chinese herbal medicines are widely used to lower serum uric acid levels. However, no systemic review summarizes and evaluates their efficacies and the underlying mechanisms of action. *Objectives.* To evaluate the clinical and experimental evidences for the effectiveness and the potential mechanism of Chinese herbal medicines in lowering serum uric acid levels. *Methods.* Four electronic databases PubMed, Wed of Science, the Cochrane Library and Embase were used to search for Chinese herbal medicines for their effects in lowering serum uric acid levels, dated from 1 January 2009 to 19 August 2020. For clinical trials, randomized controlled trials (RCTs) were included; and for experimental studies, original articles were included. The methodological quality of RCTs was assessed according to the Cochrane criteria. For clinical trials, a meta-analysis of continuous variables was used to obtain pooled effects. For experimental studies, lists were used to summarize and integrate the mechanisms involved. *Results.* A total of 10 clinical trials and 184 experimental studies were included. Current data showed that Chinese herbal medicines have promising clinical efficacies in patients with elevated serum uric acid levels (SMD: −1.65, 95% CI: −3.09 to −0.22; *p* = 0.024). There was no significant difference in serum uric acid levels between Chinese herbal medicine treatments and Western medicine treatments (SMD: −0.13, 95% CI: −0.99 to 0.74; *p* = 0.772). Experimental studies revealed that the mechanistic signaling pathways involved in the serum uric acid lowering effects include uric acid synthesis, uric acid transport, inflammation, renal fibrosis and oxidative stress. *Conclusions.* The clinical studies indicate that Chinese herbal medicines lower serum uric acid levels. Further studies with sophisticated research design can further demonstrate the efficacy and safety of these Chinese herbal medicines in lowering serum uric acid levels and reveal a comprehensive picture of the underlying mechanisms of action.

## Introduction

Hyperuricemia refers to an abnormally high concentration of serum uric acid (sUA), typically defined as >7 mg/dL in men and >6 mg/dL in women. The data from the National Health and Nutrition Examination Survey (NHANES) 2007–2016 showed that the prevalence rates of hyperuricemia were 20.2% among men and 20.0% among women between 2015 and 2016 in the United States (Chen-Xu et al., 2019). A cross-sectional survey in China showed that the prevalence of hyperuricemia was 8.4% among Chinese adults from 2009 to 2010 (Liu et al., 2014a). Hyperuricemia and gout remain as a considerable burden, which not only adversely affect patients’ health and quality of life (Burke et al., 2015; Gamala and Jacobs, 2019), but also cast an economic burden in the society (Rai et al., 2015). SUA is associated with cardiovascular diseases, such as hypertension (Kuwabara et al., 2018) and atrial fibrillation (Tang et al., 2014). Elevated sUA can lead to decreased renal function, which in turn reduces the excretion of UA in urine, resulting in an increased risk of hyperuricemia or gout (Sato et al., 2019). Elevated sUA may also contribute to the pathogenesis of metabolic syndrome (Battelli et al., 2018; Battelli et al., 2019), non-alcoholic fatty liver disease (Xu et al., 2015), diabetes (Kim et al., 2015). Urate-lowering therapy is a therapeutic strategy for controlling gout, chronic kidney disease, metabolic syndrome and many other diseases. Current interventions for elevated sUA include xanthine oxidase inhibitors, uricosuric agents and anti-inflammatory drugs. Although both febuxostat and allopurinol are effective in reducing sUA, allopurinol may produce a mild skin rash and severe cutaneous reactions (Strilchuk et al., 2019), while febuxostat has a higher risk of all-cause and cardiovascular mortality (White et al., 2018). Benzbromarone is a uricosuric drug which is widely used, but studies have reported possible complications such as hepatotoxicity (Strilchuk et al., 2019). Although these drugs are clinically used, their efficacies are unsatisfactory, and are usually coupled with adverse side effects in the long-term use (Dalbeth et al., 2016).

Hyperuricemia belongs to the arthromyodynia disease category in traditional Chinese medicine. Chinese herbal medicine has been used to treat hyperuricemia for a long time and has significant clinical efficacy (Lin et al., 2016). Chinese herbal medicines with high efficacy and low incidence of adverse reactions have drawn increasing attention from scholars. Studies have compared Chinese herbal medicine with Western medicine for their efficacies in lowering sUA levels. However, these studies differ in their treatment protocols and evaluation methodologies, which greatly limit their clinical applicability. In addition, the mechanism of Chinese herbal medicine in lowering sUA levels is still being explored. Some herbs such as *Phellodendri Chinrnsis* Cortex, *Atractylodes Lancea* (Thunb.)Dc. (Chen et al., 2015b), *Smilacis Glabrae Rhixoma* (Liu et al., 2015), reduce UA intake and/or increase UA excretion by regulating various physiological and cellular pathways. Some herbs like bergenin (Chen et al., 2020a), *alpinia oxyphylla* seed extract (Lee et al., 2019b) and *rhizoma smilacis glabrae* extracts (Liang et al., 2019), promote renal and gut uric acid excretion in hyperuricemia models and also decrease the serum levels of inflammatory cytokines. Most Chinese herbal medicines act on multiple targets to achieve their sUA lowering effects. Therefore, the aim of this systematic review is to evaluate the evidence for the efficacy of Chinese herbal medicines in lowering sUA levels in patients with hyperuricemia, compared to no intervention, placebo or urate-lowering agents, and to comprehensively summarize the mechanisms underlying the sUA lowering effects reported from experimental studies.

## Materials and Methods

### Data Source and Search Strategy

Preferred Reporting Items for Systematic Reviews and Meta-Analyses (PRISMA) (Moher et al., 2009) was used to construct the report of the current study and the completed checklist is provided in Supplementary material, Supplementary Table S1. The electronic databases PubMed, Wed of Science, the Cochrane Library and Embase were systematically searched by three researchers (Liqian Chen, Zhengmao Luo and Ming Wang). For any discrepancies between researchers, consensus was reached through discussion. We reviewed literatures published from 1 January 2009 to 19 August 2020 on elevated sUA that had been treated with Chinese herbal medicines. The following combination of terms were used as search keywords: (Traditional Chinese Medicine OR Herbal Medicine OR Chinese Herbal Drugs OR Chinese Plant Extracts OR (Plants, Medicinal) OR Phytochemicals OR herb* OR natural product) combined with (gout OR pain paralysis OR hyperuricemia OR uric acid). The full search strategy in PubMed was provided in Supplementary material, Supplementary Table S2. The search did not exclude articles based on language. To look for additional relevant studies, references of all potentially relevant articles were also retrieved, and authors of studies that met the inclusion criteria but lacked data would be contacted. Abstracts, meeting proceedings, and personal communications were not used for the purpose of this study.

### Study Selection

The articles in this review included clinical trials and *in vivo* and *in vitro* studies. When screening clinical trials, the followings are the inclusion criteria:

- A). Types of trials: All randomized controlled trials (RCTs) investigating the use of Chinese herbal medicine in lowering sUA will be eligible for inclusion.
- B). Types of participants: In accordance with the NHANES-III laboratory definition, hyperuricemia is typically defined as >7 mg/dL in men and >6 mg/dL in women (Choi et al., 2020). All adult patients (18 years and older, no upper age limit) with a diagnosis of hyperuricemia will be considered for this review.
- C). Types of interventions: According the General guidelines for methodologies on research and evaluation of traditional medicine from WHO, herbal medicines are defined as materials or products derived from plants that have medical or other beneficial effects on human health, including herbs, herbal materials, herbal products, finished herbal products that contain parts of plants or other plant materials or compositions as active ingredients, as well as materials of inorganic or animal sources. The interventions included in this study include Chinese herbal medicine in various prescriptions, such as herbal formulas, herbal extracts, active ingredients of herbs. Clinical trials comparing Chinese herbal medicine with no intervention, placebo or urate-lowering agents were included in our study. Chinese herbal medicine plus placebo or combined with urate-lowering agents compared to the same medications was also included. We do not limit the formulations or administration of herbal preparations for clinical use.
- D). Types of outcome measures: Outcome measures should include at least one essential outcome, such as change in sUA levels after treatment and the overall efficacy.

When screening *in vivo* or vitro experimental studies using the herbs, the following conditions should be met before inclusion: A) Original article investigating the use of Chinese herbal medicine in lowering sUA will be eligible for inclusion. B) Studies using experimental models of mice, rats, rabbits or cell cultures will be considered. All models used should present corresponding pathological symptoms. C) The model should only be treated with Chinese herbal medicine before or after intervention. If medication other than herbs were being used, both the treatment group and the control group must be administered. D) Outcome measures should include change in sUA after treatment.

### Data extraction

The data were extracted by three researchers (Jingru Cheng, Fei Li and Hanqi Lu) independently to obtain the following information: A) the design of study; B) characteristics of trial participants (including sample size, age, period); C) type of intervention (including dose regimen, duration); D) type of outcome measure (including the level of sUA). The reported mean (standard deviation [SD]) or risk estimates and 95% confidence intervals (CIs) of sUA were also extracted.

### Quality Assessment

The quality assessment of the studies was performed independently by three researchers (Qiuxing He, Yanting You and Xinghong Zhou), and all discrepancies between researchers were resolved through discussion. The methodological quality of RCTs was assessed according to the Cochrane Handbook for Systematic reviews of Interventions. The scores for each bias domain and the final score of risk of systematic bias were graded as low, high or unclear risk. The overall level of evidence was considered “strong” if there were consistent findings among multiple high quality RCTs, and “moderate” if findings were consistent among multiple low-quality RCTs and/or one high-quality RCT. Level of evidence was “conflicting” if findings were inconsistent across the studies, and “no evidence from trials,” if there were no RCTs.

### Statistical Analysis

Statistical analysis was performed with the software STATA version 16.0. The *d* index and the standard deviation (SD_*d*_) values of sUA for each RCT were calculated before using STATA. The *d* index and SD_*d*_ values of sUA were continuous, standard mean difference (SMD) with 95% confidence interval (95% CI) was calculated. The overall effect was calculated by a Z-test, and *p* < 0.05 (2-tailed) was deemed statistically significant. Potential heterogeneity was assessed by *I* ^2^ statistics. A fixed effects model was chosen if *I* ^2^ ≤ 50%, otherwise, a random effects model was applied. Subgroup analysis was performed to illuminate the heterogeneity according to the study characteristics, such as interventions other than Chinese herbal medicine. A ‘leave-one-out’ sensitivity analysis was carried out to test the reliability of the results. Potential publication bias was evaluated by the Egger’s test and Begg’s test. When the geometric mean and CIs were reported, tools provided in the Cochrane Handbook were used to convert the geometric mean and CIs to arithmetic mean and SD of the raw data.

## Results

### Literature Flow

**Figure 1:**
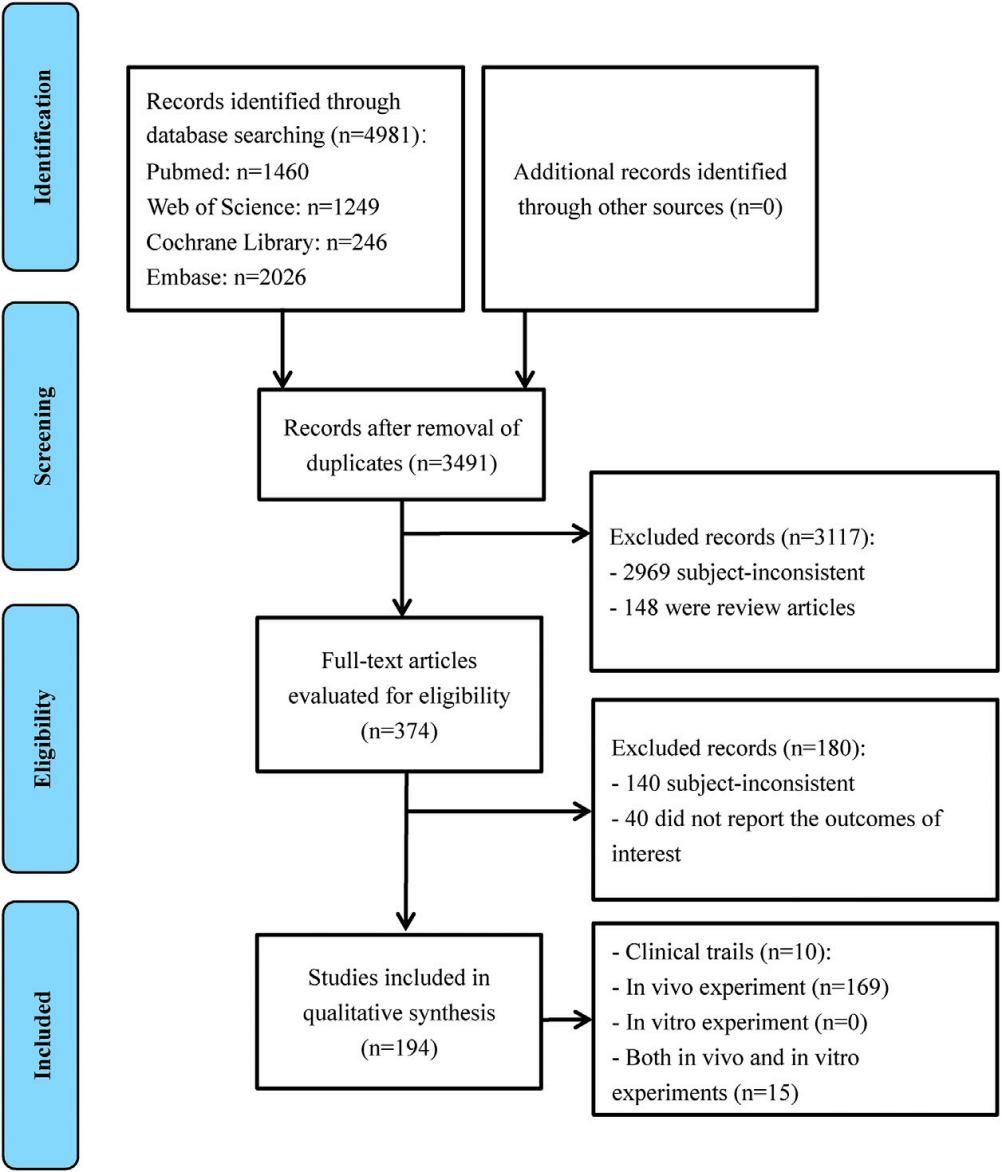
Flowchart of study selection for the systematic review.

The initial electronic search of the literature yielded 4981 potentially relevant citations. After duplicate removal and title/abstract screening, 374 full-text articles were retrieved for detailed assessment. Of these studies, 180 articles did not meet the inclusion criteria. Finally, 194 articles were included in the review with 10 clinical trials, 169 *in vivo* experiments, 0 *in vitro* experiments, and 15 that were a combination of both *in vitro* and *in vivo* experiments (Figure 1).

### Results of clinical trials

#### Study Characteristics

The specified characteristics of the included clinical trials as well as their study populations were summarized in Table 1. All clinical trials included were RCTs which mainly explored the effects of Chinese herbal medicine on patients with elevated sUA levels such as hyperuricemia and gout. In order to objectively observe the therapeutic effects of herbs on lowering sUA levels, the changes of sUA levels before and after treatment in 10 clinical trials were listed in Supplementary material, Supplementary Table S3. Of the 10 included trials, nine were treated with herbal formula, and the remaining one was treated with a combination of herbal extracts and active ingredients. In the subgroup analyses of intervention, two studies compared the therapeutic effects of Chinese herbal medicine and placebo (Rozza et al., 2016; Xie et al., 2017), three studies compared Chinese herbal medicine and Western medicine (Zhang et al., 2009; Zhou et al., 2013; Wang et al., 2014), two studies compared Chinese herbal medicine, Western medicine and placebo or no intervention (Zhang et al., 2011; Wang et al., 2019b), one compared two kinds of Chinese herbal medicine and Western medicine (Yu et al., 2018), and two compared Chinese herbal medicine combined Western medicine and Western medicine (Chen et al., 2009; Xiang et al., 2009).

**TABLE 1:** Clinical trials for herbs lowering serum uric acid.

Compounds/formula	Design	Disease	Sample size	*T*/*C*	Age (years)		Course of disease		Period	Dose regimen	Duration	Outcome	Side effect	References
					*T*	*C*	*T*	*C*						
Chuanhutongfeng mixture	RCT	Chronic gouty arthritis	165	58/55	51.10 ± 9.10	50.50 ± 8.90	9.87 ± 5.50	9.91 ± 5.42	2014.05–2015.02	125 ml, po, bid	8 weeks	sUA↓	Diarrhea	Wang et al. (2019b)
Yellow-dragon wonderful-seed formula	RCT	Gout	72	24/24	45.33 ± 9.86	49.21 ± 9.47	39.42 ± 29.00 months	42.96 ± 32.38 months	2012.03.12–2014.12.15	100 ml, po, tid	4 weeks	sUA↓	No side effect is found	Yu et al. (2018)
Yellow-dragon wonderful-seed formula + Gypsum Fibrosum	RCT	Gout	72	24/24	46.13 ± 10.75	49.21 ± 9.47	55.25 ± 36.58 months	42.96 ± 32.38 months	2012.03.12–2014.12.15	100 ml, po, tid	4 weeks	sUA↓	No side effect is found	Yu et al.(2018)
Compound tufuling oral-liquid	RCT	Gout	210	139/71	46.00 (21.00)	49.00 (19.00)	7.00 (9.00) years	5.00 (9.00) years	2012.06.09–2013.05.31	250 ml, po, bid	12 weeks	sUA↓	Leukopenia	Xie et al. (2017)
ZinutriK	RCT	HUA	16	16/16	59.00 ± 11.90		N/A	N/A	N/A	po	4 weeks	sUA↓	No side effect is found	Rozza et al. (2016)
The Chuanhu anti-gout mixture	RCT	Acute gouty arthritis	176	88/88	51.76 ± 13.21	53.82 ± 14.19	N/A	N/A	2011.09–2012.09	250 ml, po, qd	10 days	sUA↓	Diarrhea, nausea	Wang et al. (2014)
A series of tongfeng granule	RCT	Gout	90	60/30	54.10 ± 13.10	49.90 ± 14.20	55.30 ± 47.70 months	53.00 ± 47.30 months	2010.05–2011.12	Huzhang tongfeng granule: 12 g, po, bid; Yinlian tongfeng granule: 10 g, po, bid; Jinhuang Ointment: Ad us.ext, qd	12 weeks	sUA↓	Indigestion, change in frequency of urination and defecation	Zhou et al. (2013)
Xiezhuo chubi recipe	RCT	HUA	99	28/28	56.07 ± 17.62	53. 18 ± 16. 40	N/A	N/A	2009.05.01–2010.03.01	0.5 package, po, bid	20 days	sUA↓	No side effect is found	Zhang et al. (2011)
Retention enema of Chinese herbal medicine	RCT	HUA	78	40/38	51.50 ± 3.40	51.60 ± 3.30	5.40 ± 1.70 years	5.20 ± 1.90 years	2006.10–2007.12	150 ml for high enema for over 60 min, qd	6 weeks	sUA↓	No side effect is found	Chen et al. (2009)
Modified sanmiao powder	RCT	Chronic uric acid nephropathy	94	47/47	42.36 ± 15.11	44.76 ± 14.98	13.28 ± 10.63 years	14.32 ± 12.68 years	2002.06–2008.06	1 package, po, qd	12 weeks	sUA↓	ALT↑	Xiang et al. (2009)
Serial gout granules	RCT	Gout	60	30/30	52.67 ± 10.59	51.10 ± 8.43	6.49 ± 4.78 years	7.02 ± 4.86 years	2007.03–2008.03	Huzhang tongfeng granule: 12 g/package, po, bid; Yinlian tongfeng granule: 10 g/package, po,bid	12 weeks	sUA↓	Not mentioned	Zhang et al. (2009)

Data are presented a mean ± SD or a median (QR) for continuous variables and number for categorical variables. T, treatment group; C, control group; N/A: not available; sUA, serum uric acid, HUA, hyperuricemia.

#### Meta-Analysis

In order to investigate the efficacy of Chinese herbal medicine in lowering sUA, we performed a subgroup analysis based on interventions. A random effects model was used for the analysis because *I* ^2^ = 96.4%.

##### Chinese Herbal Medicine vs Placebo or No Intervention

**Figure 2:**
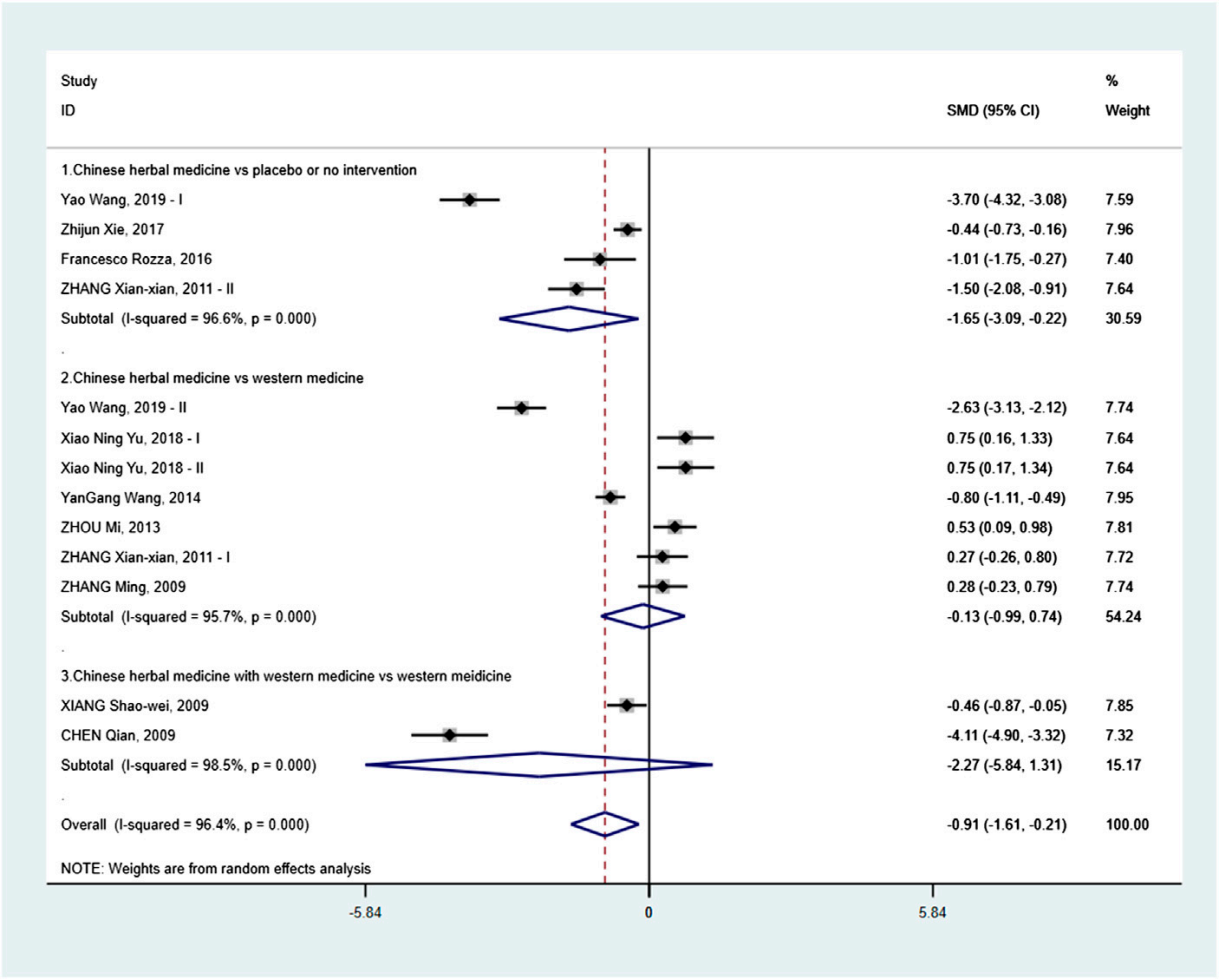
Effects of Chinese herbal medicine on serum uric acid in patients with elevated serum uric acid.

Four RCTs were analyzed. The subgroup meta-analysis showed that all of them were statistically significant differences between Chinese herbal medicine and placebo or no intervention. The combined SMD was −1.65 with a 95% CI of −3.09 to −0.22 (*p* = 0.024). Therefore, there was a significant difference between the Chinese herbal medicine and placebo or no intervention in the reduction of sUA (Figure 2).

##### Chinese Herbal Medicine vs Western Medicine

Seven RCTs were analyzed. On subgroup meta-analysis, the combined SMD was −0.13 with a 95% CI of −0.99 to 0.74 (*p* = 0.772), indicating no significant difference between the Chinese herbal medicine and Western medicine in the reduction of sUA (Figure 2).

##### Chinese Herbal Medicine Plus Western Medicine vs Western Medicine

Two RCTs were analyzed. Subgroup meta-analysis (Figure 2) showed no statistical significance between the Chinese herbal medicine plus Western medicine and Western medicine in the reduction of sUA (SMD −2.27, 95% CI: −5.84 to 1.31, *p* = 0.214).

##### Meta-regression Analyses

The meta-regression showed that samples size, duration of treatment, type of diseases and intervention other than Chinese herbal medicine did not influence these results (all *p* values >0.05) (Supplementary Figure S1).

#### Quality Assessment

RCT was assessed according to the Cochrane Handbook for Systematic reviews of Interventions (Table 2). Generally, the methodological quality was assessed to be moderate. Most of the studies (8/10, 80%) have details on the random grouping of patients, but only half of the studies (5/10, 50%) fully reported the scheme of concealment allocation. Only four trials (4/10, 40%) had their subjects and investigators blinded during the study, and four trials (4/10, 40%) had all the subjects, investigators and outcome evaluators blinded.

**TABLE 2:** Methodological quality assessment of randomized controlled trials according to the Cochrane Handbook.

Compounds/formula	A	B	C	D	E	F	G	H	References
Chuanhutongfeng mixture	+	+	+	+	+	+	+	?	Wang et al. (2019b)
Yellow-dragon wonderful-seed formula	+	+	−	−	+	+	+	?	Yu et al. (2018)
Compound tufuling oral-liquid	+	+	+	+	+	+	+	?	Xie et al. (2017)
ZinutriK	−	?	+	+	+	+	+	?	Rozza et al. (2016)
The Chuanhu anti-gout mixture	+	+	+	+	+	+	+	?	Wang et al. (2014)
A series of tongfeng granule	+	?	−	−	−	+	+	?	Zhou et al. (2013)
Xiezhuo chubi recipe	+	+	−	?	?	−	?	?	Zhang et al. (2011)
Retention enema of Chinese herbal medicine	−	−	−	−	?	+	+	?	Chen et al. (2009)
Modified sanmiao powder	+	?	−	−	?	+	+	?	Xiang et al. (2009)
Serial gout granules	+	?	−	?	?	+	+	?	Zhang et al. (2009)

A, Adequate sequence generation; B, Allocation concealment; C, Blinding (patient); D, Blinding (investigator); E, Blinding (assessor); F, Incomplete outcome data addressed; G, Selecting reporting; H, Free of other bias; +, Low risk; -, High risk; ?, Unclear.

#### Sensitivity Analysis

Sensitivity analysis was conducted to confirm the efficacy Chinese herbal medicine in lowering sUA. The pooled SMDs were repeated by sequentially removing one of the included studies with a random-effects model (Supplementary Figure S2). None of the studies changed the overall effect.

#### Publication Bias

There was no evidence of publication bias according to the Begg’s test (*p* = 0.428) and Egger’s test (*p* = 0.344) for the meta-analysis of Chinese herbal medicine on lowering sUA (Supplementary Figure S3).

### Results of experimental studies

We categorized 184 *in vivo* and *in vitro* experiments into three groups: 56 active ingredients (Table 3), 78 natural products (Table 4), and 52 herbal formulas (Table 5). Among them, one article described two active ingredients (Lin et al., 2018), and one article (Su et al., 2014) studied one active ingredient and one natural product concurrently. To assure the quality of the studies included, detailed information on herbs (including source, concentration, quality assessment, chemical analysis, and compound purity) was summarized in Supplementary material, Supplementary Tables S4–S6.

**TABLE 3:** Active ingredients on lowering serum uric acid based on *in vivo* and *in vitro* studies.

Type	Model	Active ingredients	Inducer	Animal/cell	Major findings	References
*In vivo*	HUA	Bergenin	Yeast, PO	Mice	SLC2A9, ABCG2, PPAR-γ, IL-6, IL-1β, TNF-α	Chen et al. (2020a)
*In vivo*	HUA	Phloretin	Adenine and PO	Mice	α-SMA, TGF-β, IL-1β, NLRP3, caspase-1, IL-18, XOD, URAT1, GLUT9	Cui et al. (2020)
*In vivo*	HUA	Polydatin	PO	SD rats	N/A	Han et al. (2020)
*In vivo*	HUA	Total glucosides of herbaceous peony (*Paeonia lactiflora* Pall*.*) flower	Adenine, ethambutol	Rats	XOD, URAT1, OAT1, GLUT9	Kang et al. (2020)
*In vivo*	HUA and hyperlipidemia	Total flavonoids of *Mori Cortex*	High fat diet, adenine, ethylamine butanol	SD rats	URAT1, IL-6, TNF-α, OAT1	Dang et al. (2019)
*In vivo*	Gouty arthritis	Pulchinenoside b4	MSU	SD rats	N/A	Lyu et al. (2019)
*In vivo*	Hyperuricemic nephropathy	Pterostilbene	Adenine and PO	Mice	Fibronectin, collagen I, α-SMA, TGF-β1, Smad3, Src, STAT3	Pan et al. (2019)
*In vivo* and *in vitro*	HUA	Liquiritigenin	PO, xanthine	Mice, MDCK-hOAT4, HEK293-hURAT1	OAT4, URAT1	Wang et al. (2019e)
*In vivo*	Nonalcoholic fatty liver disease and HUA	Resveratrol	PO and yeast	Rats	FOXO3a, SIRT1, NF-κB	Xu et al. (2019)
*In vivo*	Diabetic nephropathy	Total flavonoids from *Oxytropis falcata* Bunge	High-fat diet	Mice	MCP-1, NF-κB, IL-6, TGF-β1, JAK 1, STAT3, STAT 4, SOCS-1, SOCS-3	Yang et al. (2019b)
*In vivo*	Intestinal injury	*Ganoderma atrum* polysaccharide	Acrylamide	SD rats	MDA, catalase, SOD, glutathione, IL-2, IL-1β, TNF-α, IL-4, IL-10, ALP, endothelin-1	Yang et al. (2019d)
*In vivo*	HUA and acute gouty arthritis	Luteolin	PO and MSU	Mice	XO, URAT1, GLUT9, IL-1β, TNF-α	Lin et al. (2018)
*In vivo*	HUA and acute gouty arthritis	Luteolin-4′-O-glucoside	PO and MSU	Mice	XO	Lin et al. (2018)
*In vivo*	HUA and gouty arthritis	Hirudin	Hypoxanthine, sodium uric	Mice, wistar rats	XOD, GLUT9	Liu et al. (2018)
*In vivo*	HUA	Arctiin	Adenine and ethambutanol	Rats	XOD, MCP-1, TNF-α	Louxin et al. (2018)
*In vivo*	HUA	Mangiferin aglycon derivative J99745	PO	Mice	XO, URAT1, GLUT9, OAT1, ABCG2	Qin et al. (2018)
*In vivo*	HUA and gouty arthritis	[6]—shogaol	PO, MSU and hypoxanthine	SD rats	IL-1β, TNF-α	Wang et al. (2018)
*In vivo* and *in vitro*	HUA	Dioscin	PO	Mice, HCT116 cells	URAT1, GLUT9	Zhang et al. (2018b)
*In vivo*	HUA	Epigallocatechin-3-gallate	PO and yeast	Mice	URAT1, GLUT9, XO	Zhu et al. (2018)
*In vivo* and *in vitro*	HUA	Taxifolin	Mice: GMP and IMP, cells: Guanosine and inosine	Mice, AML12 cells	XO	Adachi et al. (2017)
*In vivo*	HUA	Vaticaffinol	PO	Mice	XDH, XO, GLUT9, URAT1, OAT1, OCT1, OCT2, OCTN1, NLRP3	Chen et al. (2017b)
*In vivo*	HUA	Caffeoylquinic acid	PO	Mice	XO, GLUT9, OAT1, URAT1, IL-1β, TNF-α	Jiang et al. (2017)
*In vivo* and *in vitro*	Gout	Procyanidins	Mice: MSU, cells: LPS and MSU	Mice, raw 264.7 cells	NLRP3, IL-1β, caspase-1, ROS, NR 1, p38, ERK	Liu et al. (2017)
*In vivo*	HUA	Gypenosides	Lipid emulsion	Rats	ADA, XDH, URAT1, GLUT9, OAT1	Pang et al. (2017)
*In vivo*	Gouty arthritis	Total saponin fraction from dioscorea nipponica makino	MSU	Rats	TLR2, TLR4, IRAK1, TRAF6, NF-κB, IκBα, IKKα, TAK1, IL-1β, IL-6, TNF-α	Zhou et al. (2017)
*In vivo*	HUA	Saponins extracted from dioscorea collettii	Adenine and ethambutol	SD rats	URAT1, GLUT9, OAT1, OAT3	Zhu et al. (2017)
*In vivo*	HUA	Total saponins from dioscorea septemloba thunb	Adenine	SD rats	OATP1A1	Chen et al. (2016b)
*In vivo*	HUA with renal dysfunction	Emodinol	PO	Mice	XOD, GLUT9, URAT1, ABCG2, OAT1, OCT1, OCT2, OCTN1, OCTN2, OIT3	Hui et al. (2016)
*In vivo* and *in vitro*	HUA	Pallidifloside D	PO	Mice, PC12	PRPS, HGPRT, PRPPAT	Li et al. (2016)
*In vivo*	HUA	Mangiferin	PO	Mice	XOD	Niu et al. (2016)
*In vivo*	HUA	Salvianolic acid C	PO	Mice	XOD	Tang et al. (2016)
*In vivo*	HUA	Green tea polyphenols	PO	Mice	XO, URAT1, OAT1, OAT3	Chen et al. (2015a)
*In vivo*	HUA	Flavonoids and phenylethanoid glycosides from Lippia nodiflora	PO and hypoxanthine	SD rats	XO	Cheng et al. (2015)
*In vivo*	HUA	Pallidifloside D	PO	Mice	XO, URAT1, GLUT9, OAT1	Hou et al. (2015)
*In vivo*	Gout	Lemnalol	MSU	Rats	TGF-β1, MMP-9, cathepsin K, TRAP	Lee et al. (2015)
*In vivo*	HUA and nephropathy	Rhein	Adenine and ethambutol	Mice	IL-1β, TNF-α, PGE2	Meng et al. (2015)
*In vivo*	HUA	Nuciferine	PO	Mice	URAT1, GLUT9, ABCG2, OAT1, OCT1, OCTN1, OCTN2, IL-1β, TLR2, TLR4, NF-κB, NLRP3, ASC, caspase1	Wang et al. (2015)
*In vivo*	HUA	Anthocyanins from purple sweet potato	PO	Mice	XO, URAT1, GLUT9, OAT1, OCTN2	Zhang et al. (2015)
*In vivo*	HUA	Total saponin of dioscorea	PO, ethambutol	SD rats	N/A	Chen et al. (2014b)
*In vivo*	HUA	*Chrysanthemum* flower oil	PO	Rats	XO	Honda et al. (2014)
*In vivo*	HUA	Exopolysaccharide produced by Cordyceps militaris	PO	Mice	XO	Ma et al. (2014)
*In vivo*	HUA	Dioscin	PO	Mice	OAT1, URAT1, OCT2, XO	Su et al. (2014)
*In vivo*	HUA	Pallidifloside D	PO	Mice	URAT1, GLUT9, OAT1	Wu et al. (2014a)
*In vivo*	HUA	Smilaxchinoside A, Smilaxchinoside C	PO	Mice	URAT1, GLUT9, OAT1	Wu et al. (2014b)
*In vivo*	HUA	Riparoside B, timosaponin J	PO	Mice	XO, URAT1, GLUT9, OTA1	Wu et al. (2014d)
*In vivo* and *in vitro*	HUA	Total saponins from Discorea nipponica	Mice: PO, cells: IL-1β	Mice, synovial cells	URAT1, GLUT9, OAT1, OAT3	Zhou et al. (2014)
*In vivo*	Uric acid nephropathy	Quercetin	Adenine and ethambutol	SD rats	NLRP3, ASC, Caspase-1, TLR2, TLR4	Hu et al. (2013a)
*In vivo*	HUA	Aspalathin	IMP	Mice	XO	Kondo et al. (2013)
*In vivo*	Gouty arthritis	Quercetin	MSU	SD rats	IL–1β, TNF–α, COX-2, PGE2, NO, MDA, SOD, glutathione peroxidase	Huang et al. (2012)
*In vivo*	HUA	Mangiferin	PO	Mice	XDH, XOD	Niu et al. (2012)
*In vivo*	Hyperuricemic nephropathy	Tanshinone IIA	Adenine	SD rats	NF-κB, MCP-1, IL-10	Wu et al. (2012a)
*In vivo*	Uric acid nephropathy	Tanshinone IIA	Adenine	SD rats	NF-κB, MCP-1, IL-10	Wu et al. (2012b)
*In vivo*	HUA	Curcumin	Fructose	SD rats	NO, GLUT9, RST, OAT1, MRP4, OCT1, JAK2, STAT3, SOCS3, TGF-β1	Zhang et al. (2012)
*In vivo*	HUA	Iridoid glycosides of Paederia scandens	PO and adenine	SD rats	MCP-1, α-SMA, NF-κB	Zhu et al. (2012)
*In vivo*	HUA	Mulberroside a	PO	Mice	GLUT9, URAT1, OAT1, OCT1, OCT2, OCTN1, OCTN2	Wang et al. (2011)
*In vivo*	HUA	Morin	PO	Mice	URAT1, GLUT9, OAT1, OAT3, OCT1, OCT2, OCTN1, OCTN2,	Wang et al. (2010a)

HUA, hyperuricemia; PO, potassium oxonate; MSU, monosodium urate; SLC2A9, solute carrier family 2, facilitated glucose transporter member; PPAR-*γ*, peroxisome proliferator-activated receptor *γ*; IL, interleukin; TNF-α, tumor necrosis factor; α-SMA, alpha-smooth muscle actin; TGF-β, transforming growth factor beta-1; NLRP3, the NOD-like receptor P3 inflammasome; XO/XOD, xanthine oxidase; URAT1, urate transporter one; GLUT9, glucose transporter nine; OAT1/3, organic anion transporter one and three; Smad3, mothers against decapentaplegic homolog three; Src, proto-oncogene tyrosine-protein kinase Src; STAT3, signal transducer and activator of transcription three; FOXO3α, forkhead box O3 alpha; SIRT1, silent information regulator one; NF-κB, nuclear factor-kappa B; MCP-1, monocyte chemoattractant protein-1; JAK2, Janus kinase two; SOCS-1/3, suppressor of cytokine signaling 1/3; MDA, malondialdehyde; SOD, superoxide dismutase; ABCG2, adenosine triphosphate (ATP)-binding cassette transporter two; XDH, xanthine dehydrogenase; OCT1/2, organic cation transporter; ALP: alkaline phosphatase; OCTN1/2, organic cation/carnitine transporter one and two; ROS, reactive oxygen species; NR-1, N-methyl-D-aspartic acid receptor one; p38, phosphorylated p38 mitogen-activated protein kinase; ERK, phosphorylated extracellular regulated protein kinase; ADA, adenosine deaminase; TLRs, toll-like receptors; IRAK, IL-1R-associated kinase; TRAF6, TNF receptor associated factor 6; IκB, inhibitor of NF-κB; IKKα, inhibitor of nuclear factor kappa-B kinase; TAK1, transforming growth factor beta-activated kinase one; OATP1A1, organic anion transporting polypeptide 1A1; OIT3, oncoprotein-induced transcript 3; PRPS, phosphoribosyl pyrophosphate synthetase; HGPRT, hypoxanthine-guanine phosphoribosyl transferase; PRPPAT, phosphoribosyl pyrophosphate amino-transferase; MMP-9, matrix metalloproteinase nine; TRAP, tartrate-resistant acid phosphatase; COX-2, cyclooxygenase-2; PGE2, prostaglandin E2; NO, nitric oxide; ASC, the apoptosis-associated speck-like protein containing a caspase recruitment domain; RST, renal-specific transporter; MRP4, multidrug resistance protein 4. N/A, not available.

**TABLE 4:** Natural products that lower serum uric acid based on *in vivo* and *in vitro* studies.

Type	Model	Natural product	Inducer	Animal/cell	Major findings	References
*In vivo*	HUA	*Clerodendranthus spicatus*	Oteracil potassium	Mice	N/A	Chen et al. (2020b)
*In vivo*	HUA	Sunflower head enzymatic hydrolysate	PO, yeast	Mice	XOD, ADA, URAT1	Liu et al. (2020)
*In vivo*	HUA	Macroporous Resin extract of *Dendrobium candidum* leaves	PO, adenine, yeast	SD rats	XOD, ADA, TLR4, NF-κB	Lou et al. (2020)
*In vivo*	Hyperuricemic nephropathy	Ethanol extract of *Liriodendron chinense (Hemsl.) Sarg* barks	Adenine and PO	Mice	OAT1, OAT3, ABCG2	Pan et al. (2020)
*In vivo*	HUA	Eucommia ulmoides Oliver	Mice: Oxonic acid potassium salt, rats: hypoxanthine	Mice, rats	URAT1, OAT1, OAT3, GLUT9	Fang et al. (2019)
*In vivo*	HUA	Smilax glabra extracts	PO	Mice	XOD, OAT1, OCTN2	Huang et al. (2019b)
*In vivo* and *in vitro*	HUA	*Alpinia oxyphylla* seed extract	PO	Rats, oocytes	XO, URAT1, OAT1, IL-1β, IL-6, TNF-α	Lee et al. (2019b)
*In vivo*	Chronic HUA and gout	*Rhizoma smilacis glabrae* extracts	PO and MSU	Mice	XO, IL-6, IL-12, IL-1β, IL-10, TNF-α	Liang et al. (2019)
*In vivo*	HUA	The extract of O. Sativa	PO	SD rats	XO	Liu et al. (2019)
*In vivo*	Acute gouty arthritis	Ethanolic extract of Polygonum cuspidatum	MSU	Mice	IL-1β, IL-6, TNF-α, NLRP3, ASC, caspase-1	Ma et al. (2019a)
*In vivo*	Uric acid-induced renal damage	Polygonum cuspidatum	Adenine administering and yeast feeding	SD rats	AMPK, FOXO3α, TLR4, NLRP3, MCP-1	Ma et al. (2019b)
*In vivo*	Uric acid-induced renal damage	*Polygonum cuspidatum*	Adenine, yeast	SD rats	AMPK, FOXO3α, TLR4, NLRP3, MCP1	Ma et al. (2019c)
*In vivo*	HUA	*Chrysanthemum* extract	PO	SD rats	XOD, ABCG2, URAT1, GLUT9, IL-1β, TNF-α	Peng et al. (2019)
*In vivo*	HUA	The flavonoid-rich fraction from rhizomes of Smilax glabra Roxb.	Yeast pellets and adenine	SD rats	ABCG2, OAT1, OCT2, OCTN2, URAT1, GLUT9	Wang et al. (2019a)
*In vivo*	HUA	Chicory (*Cichorium intybus L*.)	Fructose	SD rats	URAT1, GLUT9, OAT1, OAT3	Wang et al. (2019d)
*In vivo*	HUA and acute gouty arthritis	Terminthia paniculata extract	PO and MSU	Mice	XO	Yang et al. (2019c)
*In vivo*	HUA	Highly acylated anthocyanins from purple sweet potato	PO	Mice	TNF-α, IL-6, IL-1β, TGF-β1, ICAM1, COX-2, NF-κB	Zhang et al. (2019c)
*In vivo*	HUA	Ethanol extract of Dendropanax morbifera leaf	PO	Mice	XO	Cho et al. (2018)
*In vivo*	Diabetic kidney disease	*Codonopsis tangshen* Oliv.	Unilateral nephrectomy, high fat diet feeding	SD rats	IL-1β, TNF-α, IL-6	Lu et al. (2018)
*In vivo*	HUA	Ethanol extract of Cudrania tricuspidata leaf	PO	Mice	XO	Song et al. (2018)
*In vivo*	Hypouricemic and nephroprotective	*Polyrhachis Vicina Roger*	PO	SD rats	IL-1β, IL-6, TNF-α, SOD, MDA, XOD, URAT1, GLUT9, OAT1	Su et al. (2018)
*In vivo*	HUA	*Lagotis brachystachys Maxim* extract	PO	Mice	URAT1, GLUT9, OAT1	Xiong et al. (2018)
*In vivo*	HUA	Toona sinensis leaf extract	PO	Rats	XO	Yuk et al. (2018)
*In vivo*	HUA	Chaenomeles sinensis fruit extract	PO	Mice	URAT1, OAT1	Zhang et al. (2018a)
*In vivo*	HUA	Fraxini cortex	Hypoxanthine and PO	SD rats	URAT1,GLUT9	Zhou et al. (2018a)
*In vivo*	HUA	Selaginella tamariscina	Xanthine, PO and MSU	Rats	N/A	Chen et al. (2017a)
*In vivo*	HUA	Rhizoma Polygoni Cuspidati and Ramulus Cinnamomi	Hypoxanthine, ethambutol and PO	SD rats	XO	Han et al. (2017)
*In vivo*	HUA	Salvia plebeia extract	PO	Mice	XO	Kim et al. (2017)
*In vivo*	Gouty arthritis	Mollugo pentaphylla extract	MSU	Mice	TNF-α, IL-1β, NLRP3, ASC, caspase-1, NF-κB	Lee et al. (2017)
*In vivo*	HUA and gout	Sunflower head extract	PO and MSU	Mice	XO	Li et al. (2017a)
*In vivo*	HUA	Aristolochia bracteolata extracts	PO	Rats	XO	Li et al. (2017b)
*In vivo*	HUA	Total flavonoids of Humulus lupulus	PO	Mice	XO	Li et al. (2017c)
*In vivo*	HUA	Chicory	D-fructose	SD rats	ABCG2	Wang et al. (2017a)
*In vivo*	HUA	Chicory extract	Fructose	SD rats	GLUT9	Wang et al. (2017b)
*In vivo*	HUA and gout	Cortex fraxini	PO, yeast and adenine	SD rats	N/A	Wang et al. (2017c)
*In vivo*	HUA	*Plantago depressa Willd* extract	PO	SD rats	XOD	Xia et al. (2017)
*In vivo*	HUA	Ganoderma applanatum extracts	Hypoxanthine and PO	Mice	XO, URAT1, GLUT9, OAT1	Yong et al. (2017)
*In vivo*	HUA	Quercus acuta Thunb. extract	PO	Mice	XO	Yoon et al. (2017a)
*In vivo*	HUA	Ethanol extracts of Camellia japonica leaf	PO	Mice	XO	Yoon et al. (2017b)
*In vivo*	HUA and acute gouty arthritis	*Gnaphalium* affine D. Don extract	PO and MSU	Mice	XO, GLUT9, URAT1	Zhang et al. (2017a)
*In vivo*	HUA	Ginkgo folium	Fructose	SD rats	N/A	Zhang et al. (2017b)
*In vivo*	HUA	*Selaginella moellendorffii* Hieron.	PO	SD rats	XOD, MDA, SOD, TNF-α, IL-1β	Zhao et al. (2017)
*In vivo*	HUA	*Dioscorea tokoro Makino* extract	PO	Mice	XOD, URAT1, OAT1	Fei et al. (2016)
*In vivo* and *in vitro*	HUA	Mesona procumbens extracts	THP-1 cells: MSU, mice: PO, SD rats: streptozocin	THP-1 cells, mice, rats	XO, OAT1, GLUT9	Jhang et al. (2016)
*In vivo*	HUA	Ethyl acetate fraction of Dimocarpus longan Lour. extracts	PO	Mice	XO	Sheu et al. (2016)
*In vivo* and *in vitro*	HUA	Synsepalum dulcificum	Mice: PO, cells: MSU	Mice, RAW264.7 cells	XO	Shi et al. (2016b)
*In vivo*	HUA	*Dendropanaxchevalieri* extracts	PO	Mice	XOD, URAT1, GLUT9	Wang et al. (2016a)
*In vivo*	HUA	Tradescantia albiflora Kunth extracts	PO	Rats	XO	Wang et al. (2016c)
*In vivo*	HUA	Cortex fraxini	PO, yeast and adenine	Rats	N/A	Wang et al. (2016e)
*In vivo*	HUA	Cordyceps militaris	PO and hypoxanthine	Mice	XO, URAT1	Yong et al. (2016)
*In vivo*	HUA	The ethanolic extracts of Corylopsis coreana Uyeki	PO	Mice	XO	Yoon et al. (2016)
*In vivo*	HUA	Leaves of Perilla frutescens	PO	Mice	XO	Huo et al. (2015)
*In vivo*	HUA	Ethanol extracts from Dioscoreae nipponicae rhizoma	Hypoxanthine and PO	Mice	N/A	Shan et al. (2015)
*In vivo*	HUA	*Rhododendron* oldhamii maxim. Leaf extracts	PO	Mice	N/A	Tung et al. (2015)
*In vivo*	HUA	Smilax riparia	PO	Mice	URAT1, GLUT9, OAT1	Wu et al. (2015)
*In vivo*	HUA	*Lagotis brevituba Maxim*. extract	PO	Mice	XOD, OAT1, URAT1, GLUT9	Zeng et al. (2015)
*In vivo*	HUA	Total saponins from *Discorea nipponica* makino	Adenine and ethambutol	Rats	OAT1, OAT3, URAT1	Zhou et al. (2015)
*In vivo*	HUA	Phytochemicals from Davallia formosana	PO	Mice	XO	Chen et al. (2014a)
*In vivo* and *in vitro*	HUA	Smilacis glabrae rhizoma	PO and uric acid	SD rats, HUVECs	Catalase	Hong et al. (2014)
*In vivo*	HUA	*Poecilobdella manillensis*	Hypoxanthine	Mice	N/A	Liu et al. (2014b)
*In vivo*	Gouty arthritis	Rhizoma Dioscoreae nipponicae	MSU	Rats	SDF-1, CXCR4, p38, IL-1β, MKK 3/6	Lu et al. (2014)
*In vivo*	HUA	Rhizoma Dioscoreae septemlobae extracts	PO	Mice	OAT1, URAT1, OCT2, XO	Su et al. (2014)
*In vivo*	HUA	Smilax riparia	PO	Mice	URAT1	Wu et al. (2014c)
*In vivo*	HUA	Leonurus	PO	SD rats	URAT1, GLUT9, OCT, OCTN	Yan et al. (2014)
*In vivo* and *in vitro*	Gouty arthritis	Dolichos falcata Klein	MSU	SD rats, raw 264.7 cells	IL-1β, IL-6, TNF-α	Chen et al. (2013b)
*In vivo*	HUA	Ethanol extract of Fructus Gardenia	PO	Mice	URAT1, ABCG2, GLUT9, OAT1, OAT3, OIT3, OCT1, OCT2, OCTN1, OCTN2	Hu et al. (2013b)
*In vivo*	HUA and gout	The crude extract of Jatropha isabellei	PO and MSU	Rats	XO	Silva et al. (2013)
*In vivo*	HUA	Petroleum ether fraction of *Polyrhachis vicina* Roger	Hypoxanthine	Mice	XOD	Wei et al. (2013)
*In vivo*	HUA	Rhizoma smilacis glabrae	Hypoxanthine	SD rats	N/A	Xu et al. (2013)
*In vivo*	HUA	*Balanophora* laxiflora extracts and derived phytochemicals	PO	Mice	XO	Ho et al. (2012)
*In vivo*	HUA	Longan seed extract	PO and hypoxanthine	SD rats	GLUT1, GLUT9, XO	Hou et al. (2012)
*In vivo*	Gouty arthritis	Ethanol extract from *Mangifera indica*	MSU	SD rats	TNF-α, IL-1β	Jiang et al. (2012)
*In vivo*	HUA	*Hibiscus sabdariffa L.* extracts	PO	SD rats	XOD	Kuo et al. (2012)
*In vivo*	HUA	Ramulus Mori ethanol extract	PO	Mice	URAT1, GLUT9, OAT1, OCT1, OCT2, OCTN1, OCTN2	Shi et al. (2012)
*In vivo*	Acute gouty arthritis	Rhizoma Dioscoreae nipponicae	MSU and hypoxanthine	Rats	TRAIL, Neuropilin-2, MMP-13	Yao et al. (2012)
*In vivo*	HUA	The methanol extract from Prunus mume fruit	PO	Mice	XO	Yi et al. (2012)
*In vivo*	HUA	Casein or soya protein combined with palm or safflower-seed oil	PO and uric acid	Rats	NO, TNF-α, IFN-γ, Nitrotyrosine	Lo et al. (2010)
*In vivo*	Gouty arthritis	Extract of Paederia scandens (LOUR.) MERRILL (Rubiaceae)	MSU	SD rats	TNF-α, IL-1β, NF-κB	Ma et al. (2009)

Abbreviations can be referred to Table 3. ICAM1, intercellular adhesion molecule one; IFN, interferon; AMPK, adenosine 5′-monophosphate-activated protein kinase; FOXO3α, forkhead box O3 alpha; SDF-1, stromal cell-derived factor 1; CXCR4, SDF-1 receptor; MKK, mitogen-activated kinase; TRAIL, the tumor necrosis factor-related apoptosis-inducing ligand.

**TABLE 5:** Herbal formulas that lower serum uric acid based on *in vivo* and *in vitro* studies.

Type	Model	Herbal formula	Inducer	Animal/cell	Major findings	References
*In vivo*	HUA and hyperlipidemia	*Dendrobium officinalis* Six Nostrum	PO and hihg-fat sorghum feed	SD rats	XOD, ADA, LPL, FABP1, HPRT1, NLRP3, Caspase-1, TLR4	Guo et al. (2020)
*In vivo*	Gouty arthritis	Simiao decoction	Yeast, MSU	Mice	NLRP3, ACS, caspase1, XOD, ADA, IL- 1α, IL-6, IL-1β, IL-9, IL-12, granulocyte colony stimulating factor; MCP-1, TNF-α, STAT3, APOB, caspase 8, c-FOS, PPARα, FN1, LPL, MIP-1α, MIP-1β	Lin et al. (2020)
*In vivo*	Acute gouty arthritis with HUA	Tu-Teng-Cao	PO, MSU	SD rats	TNF-α, IL-6, IL-1β	Yao et al. (2020)
*In vivo*	HUA	Erding granules	PO	Mice	N/A	Zuo et al. (2020)
*In vivo*	HUA	TongFengTangSan	PO	SD rats	XOD	Chen et al. (2019b)
*In vivo*	HUA	*Alismatis Rhizoma and Rhizoma Smilacis Glabrae* decotion	PO, adenine	SD rats	XOD, URAT1	Cheng et al. (2019)
*In vivo*	HUA	Er Miao wan	Fructose	SD rats	N/A	Huang et al. (2019a)
*In vivo*	Renal stones	Huashi pill	Ethylene glycol, ammonium chloride, calcium gluconate	SD rats	Osteopontin	Yang et al. (2019a)
*In vivo*	HUA	Modified Chuanhu anti-gout mixture	PO	Mice	XOD, URAT1	You et al. (2019)
*In vivo*	Acute gouty arthritis	Jia-Wei-Si-Miao-Wan	Monosodium urate	Rats	TLR4, NLRP3, ASC, caspase-1, NF-κB, IL-1β, IL-18	Yuan et al. (2019)
*In vivo*	HUA	Erding granule	PO	Mice, SD rats	GLUT9, OAT1, URAT1	Zhang et al. (2019a)
*In vivo* and *in vitro*	HUA and acute gouty arthritis	The *Selaginella moellendorffii* prescription	PO, adenine, MSU, LPS	Mice, rats, RAW264.7 cells	NO, NF-κB, NLRP3, IL-1β, PGE-2, IL-8	Zhang et al. (2019b)
*In vivo*	Acute gouty arthritis	Tongfengning capsule	MSU	Rats	TNF-α, IL-1β	Fan et al. (2018)
*In vivo*	T2DM	Spleen-kidney supplementing formula	High-fat diet, low-dose streptozotocin	Wistar rats	TGF-β1, miR-21, PTEN	Tian et al. (2018)
*In vivo*	HUA	Ermiaowan	Xanthine and oxonic acid potassium salt	Rats	N/A	Wei et al. (2018)
*In vivo*	HUA	Compound tufuling granules		Rats	TNF-α, IL-1β	Wu et al. (2018a)
*In vivo*	HUA	ShiZhiFang	PO	SD rats	XOD, OAT1, OAT3	Wu et al. (2018b)
*In vivo*	HUA	Erding formula	Hypoxanthine and PO	Mice	URAT1, OAT3	Zuo et al. (2018)
*In vivo*	HUA	Qi-Zhu-Xie-Zhuo-Fang	Adenine and PO	Rats	XO, E-cadherin, α-SMA	Huijuan et al. (2017)
*In vivo*	HUA	Shizhifang	PO	SD rats	TXNIP, NLRP3, ASC, caspase-1, IL-1β, IL-18, ROS	Wu et al. (2017)
*In vivo*	HUA	Quzhuotongbi decoction	Yeast	SD rats	N/A	Chen et al. (2016a)
*In vivo*	Gouty arthritis	Zisheng Shenqi decoction	MSU	Rats	IL-1β, TNF-α, IκB, NF-κB, NALP1, NALP6, ROS	Han et al. (2016)
*In vivo*	HUA	RuPeng15 powder	PO	SD rats	XO	Kou et al. (2016)
*In vivo*	Gouty arthritis	Xiaofeng granules	MSU	SD rats, rabbits	IL-1β, IL-6, TNF-α, iNOS, ADAMTS-4, TIMP-3	Shi et al. (2016a)
*In vivo*	HUA	Siwu decoction	PO	Mice	XO, URAT1, GLUT9, OAT1, ABCG2, OCT1, OCT2, OCTN1, OCTN2, NLRP3, ASC, caspase-1, IL-1β	Wang et al. (2016b)
*In vivo*	HUA	Jianpi Huashi decoction	PO	Rats	XO	Wang et al. (2016d)
*In vivo* and *in vitro*	Gout	Sanmiao formula	Animals: PO and cold bath, cells: MSU	SD rats, rabbits, primary chondrocyte	IL-1β, TNF-α, MMP-3, TIMP-1, ADAMTS-4, TIMP-3	Zhu et al. (2016)
*In vivo*	HUA	Karapxa decoction	Yeast and PO	Mice	XO	Amat et al. (2015)
*In vivo*	HUA	Ermiao pill	PO	SD rats	XO	Chen et al. (2015b)
*In vivo*	HUA	Si-Wu-Tang and Er-Miao-San	PO and adenine	SD rats	XO, OAT1, OAT3	Guo et al. (2015)
*In vivo*	HUA	Shuang-Qi gout capsule	PO	Mice	URAT1, OAT1, OCT1, OCT2, ABCG2, OCTN1, OCTN2, GLUT9	Kodithuwakku et al. (2015)
*In vivo*	Gouty arthritis	RuPeng15 powder	MSU	SD rats	NF-κB, TNF-α, IL-1β, IL-8	Kou et al. (2015)
*In vivo*	HUA	Compound tufuling granules	Yeast and PO	Mice	GLUT9	Liu et al. (2015)
*In vivo*	HUA and metabolic syndrome	Simiao pill	Fructose	SD rats	Nephrin, podocin, CD2AP, Sirt1, NF-κB, IL-1β, NLRP3, ACS, caspase1	Ma et al. (2015)
*In vivo*	HUA	Xie-Zhuo-Chu-Bi-Fang	Adenine and PO	Mice	URAT1, miR-34a	Sun et al. (2015)
*In vivo*	HUA	Wuling san	Fructose	Mice	URAT1, GLUT9, ABCG2, OAT1, OCT1, OCT2	Yang et al. (2015)
*In vivo*	Urate nephropathy	Compound qingqin liquid	Adenine and PO	SD rats	TLR2, TLR4	Chen et al. (2014c)
*In vivo*	HUA	Modified Simiao wan	Oxonic acid potassium salt, hypoxanthine	SD rats	XOD	Pan et al. (2014)
*In vivo*	HUA	Compound qingqin liquid	Adenine and PO	SD rats	COX-2	Shang et al. (2014)
*In vivo*	HUA	Jianpihuashi decoction	PO	SD rats	XO	Chen et al. (2013a)
*In vivo*	HUA	Wuling san	PO	Mice	URAT1, GLUT9, OAT1, OCT1, OCT2, OCTN2	Ding et al. (2013)
*In vivo* and *in vitro*	Gout arthritis	Shuang-Qi gout capsule	MSU	Mice, SD rats, HUVECs	TNF-α, IL-1β	Kodithuwakku et al. (2013)
*In vivo*	HUA	Tongfeng granule	Adenine and ethambutol	Rats	N/A	Liu et al. (2013)
*In vivo*	HUA	Jieduxiezhuo decoction	Uric acid	Mice	N/A	Pan et al. (2013)
*In vivo* and *in vitro*	MSU-induced inflammation	Modified Simiaowan	MSU	Mice, HUVECs	ICAM-1	Shi et al. (2013)
*In vivo*	HUA	Tongxi powder	Uric acid	Mice	N/A	Yang et al. (2013)
*In vivo*	HUA	Modified Simiao decoction	PO	Mice	XO, URAT1, OAT1, SOD, MDA	Hua et al. (2012)
*In vivo*	Acute gouty arthritis	Compound Shuiniujiao	MSU	SD rats	N/A	Cao et al. (2011)
*In vivo*	HUA	Simiao pill	PO	Mice	URAT1, GLUT9, OAT1, OCT1, OCT2, OCTN1, OCTN2	Hu et al. (2010)
*In vivo*	HUA	Danggui Buxue Tang	Adenine, ethambutol	Rats	NO, NOS, SOD, MDA	Li et al. (2010)
*In vivo*	HUA	Ermiao pill	Oxygen hydrochloride acid potassium salt	Mice	XOD, URAT1	Lü et al. (2010)
*In vivo*	HUA	Sanmiao wan	PO	Mice	XOD, URAT1	Wang et al. (2010b)

Abbreviations can be referred to Tables 3, 4. FABP1, fatty acid-binding protein; HPRT1, hypoxanthine-guanine phosphoribosyl transferase; APOB, apolipoprotein B; FOS, one subunit of activator protien-1; FN1, fibronectin1; MIP-1α, MIP-1 β, serum proinflammatory cytokines; TXNIP, thioredoxin interacting proteins; PTEN, phosphate and tension homology deleted on chromsome ten; NALP1/6, NACHT, LRR and PYD domains-containing protein one and six; ADAMTs, a family of metalloproteinases with thrombospondin motifs; TIMP, tissue inhibitor of metalloproteinase; CD2AP, CD2-associated protein.

#### Active Ingredients That Lower Serum Urate *in vivo* and *in vitro* Studies

Active ingredient is a single ingredient and studies have shown that it plays an important therapeutic role in reducing sUA. Among the active ingredients with potential of lowering sUA (Table 3), 25 of which act on uric acid synthesis, 28 target uric acid transporter, 19 resolve inflammation, nine possess kidney protective function, and four regulate the oxidative stress.

#### Natural Products That Lower Serum Urate *in vivo* and *in vitro* Studies

Natural products include herbs and relatively complex extracts derived from herbs. Among the natural products with potential of lowering sUA (Table 4), 40 of which act on uric acid synthesis, 29 target uric acid transporter, 17 resolve inflammation, eight possess kidney protective functions, and three regulate the oxidative stress.

#### Herbal Formulas That Lower Serum Urate *in vivo* and *in vitro* Studies

Herbal formula is a combination of a variety of herbs. The composition of the herbal formula included in the current study were listed in Supplementary material, Supplementary Table S7. Among the herbal formula with lowering sUA effects (Table 5), 18 of which act on uric acid synthesis, 15 target uric acid transporter, 15 resolve inflammation, seven possess kidney protective functions, and five regulate the oxidative stress.

## Discussion

### The efficacies of Chinese herbal medicines lowering Serum uric Acid levels

This systematic review compares the efficacies of the Chinese herbal medicines and the Western medicine in lowering sUA levels by analysing10 RCTs with a total of 1,060 patients. The meta-analysis has three important findings. First, there is a significant difference between the Chinese herbal medicine, placebo or no intervention in the reduction of sUA levels. Second, the efficacies of Chinese herbal medicines in lowering sUA levels are comparable to that of Western medicine. Third, the efficacies of Chinese herbal medicines plus Western medicine in lowering sUA levels are comparable to that of Western medicine. The heterogeneity of the meta-analysis was high. To investigate the source of heterogeneity in our analysis, we conducted a sensitivity analysis, removing one study at one time from the primary analysis did not change the main finding. In addition, our findings were confirmed by the lack of publication bias and effect modifiers according to the Begg’s test, Egger’s test and meta-regression analysis.

The results indicate that the efficacies of Chinese herbal medicines in lowering sUA levels are comparable to that of Western medicine, which is consistent with the analysis result of Lin et al. (Lin et al., 2016). Compared with the study of Lin et al., our study is more comprehensive, because we also compared the sUA lowering efficacy between Chinese herbal medicine and placebo or no intervention, and between Chinese herbal medicine plus western medicine and western medicine. In addition, we have summarized the underlying mechanisms of herbs in lowering sUA. It should be noted that among the 10 RCTs included, a total of six RCTs used one or several components of Simiao Pills, which is a famous formula in traditional Chinese medicine, including *Cortex Phellodendri Chinensis*, *Atractylodes Lancea* (*Thunb*.) DC., *Coix lacryma-jobi L.var.ma-yuen* (*Roman.*) Stapf (*Yi Yi*), *Cyathula officinalis Kuan* (Xiang et al., 2009; Zhang et al., 2009; Zhang et al., 2011; Zhou et al., 2013; Xie et al., 2017; Yu et al., 2018). The specific doses of herbs in each study have been listed in the Supplementary Tables S4–S6. According to the Pharmacopoeia of the People’s Republic of China revised by the Food and Drug Administration in 2015, except for the use of *Cortex Phellodendri Chinensis*, *Atractylodes Lancea* (*Thunb*.) DC., *Cyathula officinalis Kuan* in one RCT (Xiang et al., 2009) exceeded the recommended dosage, the dosages of these four herbs in the remaining five RCTs were within the recommended dosage range. Except for the RCT in which the dosage exceeded the recommended dosage (Xiang et al., 2009), the dosage of these four herbs in the remaining five RCTs were all at the high level or even reached the critical value within the recommended range. Despite the high dosage, the three RCTs describing the adverse reactions in the six RCTs showed that the incidence of adverse reactions was either lower than that of the placebo group or was not statistically significant compared with the control group.

In addition to clinical studies, experimental studies have also suggested the urate lowering effects of Simiao Pills, which act on xanthine oxidase (XO), uric acid transport-related proteins urate anion transporter 1 (URAT1) and glucose transporter 9 (GLUT9), and also modulate the inflammation, oxidative stress and other processes (Hu et al., 2010; Hua et al., 2012; Shi et al., 2013; Pan et al., 2014; Ma et al., 2015; Lin et al., 2020). The finding suggests that Chinese herbal medicines are mostly multi-targeted or have interplay with other signaling pathways to lower sUA levels. In contrast, conventional Western medicines used to lower sUA levels include allopurinol, probenecid and benzbromarone, which generally act on specific targets. Allopurinol competitively inhibits xanthine oxidase (Strilchuk et al., 2019). Probenecid and benzbromarone are typical urate-promoting drugs target at URAT1 (Azevedo et al., 2019). These may partly explain why the combination of Chinese and Western medicine can improve the index of renal function while lowering sUA levels in patients with hyperuricemia (Chen et al., 2009; Xiang et al., 2009).

Nevertheless, several limitations of the meta-analysis are worth considering. Due to limited reports, participants with elevated sUA levels in this meta-analysis included patients with hyperuricemia and gout, and most participants were from China. These facts indicate that the current meta-analysis could have potential bias. Further trials need to be carried out in a larger comprehensive population to demonstrate the efficacy of Chinese herbal medicines in lowering sUA. In addition, long-term tracking of sUA is necessary to determine whether Chinese herbal medicine can effectively control sUA. However, long-term follow-ups were not available in the current included studies. The incidence of adverse reactions is an important indicator to compare the efficacy and safety of Chinese herbal medicine and Western medicine in lowering sUA levels. However, of the six included studies comparing Chinese herbal medicine and Western medicine, only 2 has reported the adverse reaction events in details. Thus, we cannot evaluate the safety of Chinese herbal medicine vs. western medicine in lowering sUA levels. Moreover, the standardization of the methodologies and the small number of the included trials may lead to an overestimation of the overall efficacy of Chinese herbal medicine. Therefore, studies with high quality are needed to confirm the efficacy and safety of Chinese herbal medicine in lowering sUA levels.

### The mechanisms of Chinese herbal medicines in lowering Serum uric Acid

In current review, 186 *in vivo* and *in vitro* experiments with 56 active ingredients (Table 3), 78 natural products (Table 4), and 52 herbal formulas (Table 5) are included to explore the common mechanism of Chinese herbal medicine in lowering sUA levels. According to the summary of the targets of Chinese herbal medicine in lowering sUA, it is clearly revealed that most Chinese herbal medicine lowers sUA by acting on multiple targets or multiple pathways. The therapeutic mechanism of Chinese herbal medicine included in this study mainly involved the UA synthesis, UA transport, inflammation, renal fibrosis and oxidative stress.

Uric acid, produced primarily in the liver, is the final product of diet and endogenous purine metabolism. Problems with key enzymes involved in UA production can cause abnormal UA levels, including phosphoribosyl pyrophosphate (PRPP) synthetase, purine nucleoside phosphorylase, xanthine oxidase, hypoxanthine-guanine phosphoribosyl transferase (HGPRT) (Cammalleri and Malaguarnera, 2007; Maiuolo et al., 2016). In the circulation, UA exists mainly in the form of urate anion under physiologic pH. The saturation level of monosodium urate in human plasma is limited. Hence, UA must be excreted continuously to prevent its accumulation and reduce the toxicity. The primary scavenger of urate is the kidney, which expels about 75% of urate every day (Chaudhary et al., 2013). In recent years, studies have revealed the complex interaction of transporters involved in urate metabolism (Wright et al., 2010). Several transporters involved in urate metabolism have been identified, including URAT1, GLUT9, organic anion transporters (OAT1/3), and adenosine triphosphate (ATP)-binding cassette transporter 2 (ABCG2) (Enomoto et al., 2002; Lipkowitz, 2012; Mandal and Mount, 2015). Previous studies have found that inflammation, oxidative stress, mitochondrial dysfunction and other factors cause abnormalities in these critical proteins, and thus lead to the disorders in UA metabolism (Lanaspa et al., 2012; Zhou et al., 2018b). On the contrary, abnormal UA levels can also lead to the release of some cytokines, such as tumor necrosis factor-α (TNF-α), nuclear factor-kappa B (NF-*κΒ*), the NOD-like receptor P3 (NLRP3) inflammasome, interleukin-6 (IL-6), interleukin-1β (IL-1β), and so on (Major et al., 2018; Singh et al., 2019).

#### Herbs Lower Serum Uric Acid by Targeting Uric Acid Synthesis

The substrate for UA synthesis is ribose-5-phosphate which can be converted to PRPP via PRPP synthase and then to inosine monophosphate. This intermediate compound produces adenosine monophosphate and guanosine monophosphate, which subsequently release adenosine and guanosine molecules, respectively. Adenosine deaminase converts adenosine to inosine, while guanosine to free guanine. Inosine is degraded to hypoxanthine via purine nucleoside phosphorylase. Xanthine oxidase, one of the key enzymes involved in UA synthesis, converts hypoxanthine to xanthine which then converts to UA. Guanine is directly converted to xanthine, which subsequently to UA by XO. On the other hand, hypoxanthine and guanine enter a salvage pathway through the activity of HGPRT, which converts these purine into their respective nucleotides (Lipkowitz, 2012; Lima et al., 2015; Maiuolo et al., 2016). Thus, PRPP synthase, XO and HGPRT are all key enzymes that can cause abnormal sUA levels. PRPP gene mutations have been implicated in a number of human diseases. Overexpression of PRPP results in the enhanced activity of phosphoribosyl pyrophosphate synthetase-I, which can lead to excessive production of purine. Patients with active phosphoribosyl pyrophosphate synthetase-I may result in UA over production (Mittal et al., 2015). Lesch-Nyhan disease is caused by a wide variety of mutations in the HGPRT gene and is one of the models of gout caused by the increased production of UA (Fu et al., 2014; Harris, 2018). Lack of HGPRT can lead to Kelley-Seegmiller syndrome, which is characterized by hyperuricemia, hyperuricosuria, gouty arthritis and urolithiasis (Chavarriaga et al., 2019). Among these key enzymes, XO is the most commonly studied enzyme. 3,5,2′,4′-tetrahydroxychalcone significantly inhibits the activities of XO in liver, and the decreased content of PRPP in liver will suppresse the UA production (Niu et al., 2018). In our review, nearly half of the studies (83/186, 45%) confirm that herbs regulate XO synthesis and activity. For example, pallidifloside D, a saponin glycoside constituent from the total saponins of Smilax riparia, enhances the hypouricemic effect of allopurinol by regulating XO activity. The combination of allopurinol and Pallidifloside D significantly up-regulates HGPRT expression and down-regulates the expressions of PRPP in PC12 cells (Li et al., 2016).

#### Herbs Lower Serum Uric Acid by Targeting Uric Acid Transporter

About two-thirds of UA is excreted by the kidneys, and the remaining is excreted via the gastrointestinal tract. Renal excretion of UA consists of four steps: 1**)** glomerular filtration, 2**)** presecretory reabsorption, 3**)** secretion and 4**)** post-secretory reabsorption (Maiuolo et al., 2016). Urate is filtered through the glomerulus, so the reabsorption and secretion of UA after glomerulus play an important role in regulating the excretion of UA. Most of the urate (99%) filtered through the glomerulus is reabsorbed in the early S1 segment of the proximal tubule (presecretory reabsorption). Uric acid is then secreted in the S2 segment of the proximal tubule to return approximately 50% of the filtered urate into the tubule lumen. Post-secretory reabsorption occurs primarily in the distal S3 segment of the proximal tubule, followed by about 10% of the secreted urate in the urine (Diamond and Paolino, 1973; Mandal and Mount, 2015). Urate transporters such as URAT1, OAT1, OAT3, GLUT9, ABCG2 play important roles in regulating sUA, and their dysfunction may cause abnormal urate transport. URAT1, the main urate-anion exchanger in the luminal membrane of the proximal tubules, can be inhibited by the uricosuric agents, likes probenecid, benzbromarone and losartan (Enomoto et al., 2002). URAT1 mutations have been found in patients with familial hypouricemia and UA levels below 1 mg/dL, and the UA transport is inactivated when this mutant transporter is expressed in *xenopus* oocytes (Dinour et al., 2011). Clinical studies have shown that inhibition of URAT1 effectively reduces sUA levels and resolves the gout symptoms (Lee et al., 2019a). URAT1 is a branch of the organic anion transporter (OAT). OAT1 and OAT3 exchange urate with bivalent anions, suggesting that they are suitable for basolateral entry of urate during urate secretion (Mandal and Mount, 2015). Among the 14 members of the GLUT family transport glucose or other monosaccharides, GLUT9 does transport essentially urate (Caulfield et al., 2008). GLUT9 mediates urate transport, which is independent of sodium, chlorine and anions, but voltage-dependent (Anzai et al., 2008). Compared with the mutation in URAT1, the complete loss of GLUT9 results in the net secretion of urate (Preitner et al., 2009). ABCG2 is a multidrug resistance transporter that is also implicated as an important urate transporter. Its gene variation has become the main cause of elevated sUA levels (Dehghan et al., 2008). ABCG2-mediated loss or reduction of renal urate secretion will lead to increased renal urate reabsorption (Woodward et al., 2009).

In our review, more than one-third of the studies (72/186, 39%) show that Chinese herbal medicines target uric acid transporters. *Alpinia oxyphylla* seed extract enhances UA excretion in the kidney by reducing URAT1 and up-regulating OTA1 (Lee et al., 2019b). Wuling San down-regulates mRNA and protein levels of URAT1 and GLUT9, as well as up-regulates OAT1 in the kidney of hyperuricemic mice. Moreover, Wuling San also up-regulates organic cation/carnitine transporters which are associated with impaired renal function, leading to kidney protection (Ding et al., 2013). Gypenosides, natural saponins extracted from Gynostemma pentaphyllum, significantly lower sUA levels by reducing XO and increasing in urate excretion through regulating URAT1, GLUT9, and OAT1 (Pang et al., 2017).

#### Herbs Lowering Serum Uric Acid Resolve Inflammation

Uric acid belongs to the damage-associated molecular patterns, altered metabolites of necrotic or stressed cells that the innate immune system sees as an alarm signal (Patel, 2018). Elevated UA levels alters the physiology, boosting the expressions of inflammatory proteins by triggering complex pro-inflammatory cascades that damage cells and tissues (Chen and Lan, 2017). Clinical trials have shown that serum levels of IL-6 and TNF-α are significantly higher in hyperuricemia patients than in healthy people, and that of IL-6 and TNF-α are significantly increased as sUA levels increase (Zhou et al., 2018b). SUA above 9 mg/dl is associated with a gouty arthritis incidence of 4.9%. The accumulation of monosodium urate (MSU) crystals induce a mass of inflammatory cells (such as neutrophils and monocytes) to infiltrate into the site of MSU crystal deposits in patients, resulting in an acute inflammatory response, manifested as acute gout flares (Dalbeth et al., 2016). IL-1 released from these immune cells further triggers the release of various pro-inflammatory cytokines and chemokines, such as IL-8, IL-6 and TNF-α, which can further enhance neutrophil recruitment (El Ridi and Tallima, 2017). Inflammatory cytokines, especially IL-1β, are the key mediators of gouty inflammation. A phase III, international safety study of patients with acute gout arthritis treated with rilonacept (an IL-1 blocker) for 16 weeks shows that rilonacept significantly reduces the risk of gout attacks by 70.3% (Sundy et al., 2014). In experimental studies included in this review, 37 articles (11 active ingredients, 13 natural products, and 13 herbal formulas) describe the effects of these herbs on IL-1. It is worth mentioning that these herbs not only act on IL-1, but also as XO, UA transporter, other inflammatory factors such as IL-6, IL-8, TNF-α, NF-κB, NLRP3, caspase 1, etc.

There are 16 herbs included in this review that interfere with the NLRP3 inflammasome. Consistent with reported studies, elevated UA can be effectively reduced by regulating NLRP3 inflammasome - IL-1 pathway (Dhanasekar and Rasool, 2016; Szekanecz et al., 2019). Acute gout is an inflammatory response to MSU crystals. Innate immune pathways are essential in the pathogenesis of gout, particularly the activation of NLRP3 inflammasome, which leads to the release of IL-1β and other pro-inflammatory cytokines (So and Martinon, 2017). MSU crystals must first be coated with serum proteins and then interact with articular cell’s surface membrane directly or via receptors, to stimulate a cytosolic molecular platform involved in innate immunity and promote the assembly and activation of the NLRP3 inflammasome (El Ridi and Tallima, 2017). NLRP3 inflammasomes are formed by the recruitment of the apoptosis-associated speck-like protein containing a caspase recruitment domain (ASC), and subsequent recruitment of caspase-1. Caspase-1 activates pro-inflammatory cytokines IL-1β and IL-18 by cleaving their respective precursor proteins, pro-IL-1β and pro-IL-18 (So and Martinon, 2017). Neutrophils are recruited and activated in response to the spillover of IL-1, producing ROS, proteolytic enzymes, pro-inflammatory chemokines, cytokines and so on, which recruit and activate macrophages (El Ridi and Tallima, 2017). Thus, the release of IL-1β mediated by inflammasome and the rapid recruitment of neutrophils lead to acute inflammatory episodes in gout patients. It is noteworthy that hyperuricemia may stimulate inflammatory leukocytes through epigenetic modification such as histone methylation, even without MSU crystals, thereby increasing production of IL-1β, IL-6, and TNF-α (Wasilewska et al., 2012; Zhou et al., 2018b).

The activation and maturation of IL-1β in response to endogenous and exogenous stimuli are also involved in the NF-κB pathway (Chen and Lan, 2017). In our review, there are 13 articles focused on NF-κB signaling pathway, showing the inhibitory effect of herbs on NF-κB activation under elevated sUA levels. It has been proved that NF-κB can be activated by UA (Spiga et al., 2017). Its activation transcribes a large number of pro-inflammatory genes. In a resting state, NF-κB combines with IκBα (inhibitor of NF-κB kinase subunit α) form a dimer in the cytoplasm, after IκBα kinase is activated, it phosphorylates IκBα. Subsequently, NF-κB is transferred from the cytoplasm to the nucleus, leading to the transcription and expression of genes related to inflammation. Studies have shown that inhibition of NF-κB restrains inflammation and improves hyperuricemia or gout conditions (Chen et al., 2019a; Wang et al., 2019c). Interestingly, studies focused on NF-κB activity is also related to NLRP3. NF-κB is essential for the initiation, assembly and activation of NLRP3 inflammasome, which is also a key step in the release of inflammatory cytokines in MSU crystal-induced inflammation (So and Martinon, 2017). P38 mitogen-activated protein kinase (MAPK) is also involved in the inflammatory cascade of NF-κB. Uric acid activates NF-κB through MAPK signal pathway, thus leading to the release of inflammatory factors such as TNF-α (Tang et al., 2017).

#### Herbs Lowering Serum Uric Acid Protect Against Renal Fibrosis

Evidence suggests that UA levels can be used to predict the prognosis of chronic kidney disease and end-stage kidney disease. Elevated sUA level is an independent risk factor for kidney disease and lead to renal fibrosis (Fan et al., 2019). Mice with systemic GLUT9 knockout showed moderate hyperuricemia, excessive hyperaciduria and obstructive nephropathy, along with progressive inflammatory fibrosis (Preitner et al., 2009). Once UA exceeds the maximum amount the kidneys can excrete, it gets deposit in the kidney and firstly causes direct pathological damage to the kidney. Secondly, the deposition of UA in the kidney results in the accumulation of neutrophils and monocytes as well as the release of inflammatory factors, which lead to glomerulosclerosis and interstitial fibrosis and aggravate renal injury (Ye et al., 2018). Prevention and treatment of renal fibrosis is the best treatment for kidney diseases caused by elevated sUA. However, at present, modern medical treatments are not effective in reducing renal fibrosis and preventing the progression of diseases.

A total of 24 studies included in this review are focused on hyperuricemic nephropathy, suggesting that herbal medicines improve renal injury induced by elevated sUA levels by regulating renal fibrosis-related signal pathways. Fibrosis is usually associated with strong inflammatory reactions and immunocyte infiltration. Therefore, inhibition of inflammatory cytokines might be a potential method to prevent fibrosis. Vaticaffinol, one of the herbs included in this review, markedly down-regulates NLRP3, ASC, caspase-1, IL-1β, IL-18, IL-6 and TNF-α in hyperuricemic mice, thus significantly decreases sUA levels and improves kidney function (Chen et al., 2017b). Transforming growth factor-*β*1/Mothers against decapentaplegic homolog 3 (TGF-β1/Smad3) signaling is the most potent fibrogenic factor in the regulation of renal interstitial fibrosis process (Loeffler and Wolf, 2015). Pterostilbene, also mentioned in this review, suppresses the activation of TGF-β1/Smad3 and proto-oncogene tyrosine-protein kinase Src/Signal transducer and activator of transcription 3 (Src/STAT3) signaling pathway as to decrease sUA level and alleviate renal fibrosis in hyperuricemic mice (Pan et al., 2019). These findings highlight the fact that herbal medicines may be the potential antifibrotic therapeutics for hyperuricemic nephropathy treatment.

#### Herbs Lowering Serum Uric Acid Modulate Oxidative Stress

In addition to the inflammatory process, oxidative stress is one of the early events related to the elevated sUA. Uric acid entering cells can rapidly induce oxidative stress (Ko et al., 2019; Yin et al., 2019). This state of oxidative stress is governed by the balance between ROS production and their elimination by antioxidants. Since the cell membrane is impermeable to urate anion, cellular concentration of urate depends on the specific transporter of urate and xanthine oxidoreductase (XOR). In mammals, this enzyme exists in two forms, xanthine dehydrogenase (XDH) and XO. XDH transfers electron to NAD^+^ and generates NADH, and XO transfers electron to O_2_ and generates oxidative stress (Isaka et al., 2016). In addition, XOR may lead to the production of the superoxide anion and nitric oxide, especially under low pH or hypoxia conditions (Godber et al., 2000; Battelli et al., 2019). It can be seen that the generation of UA mediated by XO is closely related to the production of ROS. On the other hand, antioxidant enzymes that scavenge ROS are ubiquitous, including superoxide dismutase, glutathione peroxidase and catalase, the changes of these enzymatic activities may lead to oxidative stress. However, in inflammatory diseases including hyperuricemia, the production of the superoxide anion is often faster than its removal by superoxide dismutase (Zamudio-Cuevas et al., 2015).

There is an increasing interest in using herbs to resolve the inflammatory conditions, including hyperuricemia, because they play an anti-inflammatory role by inhibiting the production of ROS, such as procyanidins, Shizhifang, Zisheng Shenqi decoction, quercetin, Modified Simiao decoction and other herbs included in this review. Antioxidants like quercetin inhibits inflammation in rat models of chronic MSU-induced arthritis by decreasing inflammatory mediators such as IL-1β (Huang et al., 2012). Other herbs mentioned above also show significant antioxidant effects. For example, Shizhifang effectively suppresses the NLRP3-ASC-caspase-1 axis through accommodating the ROS pathway, thereby alleviates potassium oxonate - induced hyperuricemia (Wu et al., 2017). In summary, the herbs that possess anti-oxidant effects may be developed as anti-inflammatory agents to treat inflammatory diseases such as gout and hyperuricemia.

#### Limitations and Perspectives From the Experimental Studies

As shown in Tables 3–5, there are fewer studies on the active ingredients of herbs than on the herbal extracts and herbal formulas. Aside from herbal formula, there can be hundreds of active ingredients in a single-flavored herb, making it difficult to identify the ones that actually mediate the therapeutic effects. This partly explains why multiple signaling pathways are involved in herbal treatments. Extraction of components that mediate the therapeutic effects is difficult. In general, safe and effective single-flavored herb can be screened out from literatures or experimental studies, and its effective active ingredients can be separated by pharmacological methods. Then, the mechanism of action of the drug can be explored based on the identified active ingredients from the herbs. In recent years, there are new approaches to identify the active ingredients of herbs and predict their targets, such as Systems Pharmacology. Researchers used Systems Pharmacology as a basis to screen out active ingredients from herbal formulas or herbs, and predict the targets of active ingredients, and finally verify them through *in vivo* and *in vitro* experimental studies (Zhou et al., 2016; Hong et al., 2017; Zhao et al., 2019). It really opens up new ideas for the studies of herbs and shortens the time to find out the mechanisms of action underlying the therapeutic effects of the herbs. However, with the increasing number of herbal studies using Systems Pharmacology, we can find that it also has disadvantages, such as limited Systems Pharmacology databases, data not updated, limited prediction results and so on. In view of this, it is still necessary to explore how to efficiently screen out the active ingredients of the herbs and identify their molecular targets.

## Conclusion

In conclusion, the results of meta-analysis indicate that Chinese herbal medicines have potent therapeutic effects in lowering sUA levels. The signaling pathways involved in the sUA lowering effects include UA synthesis, UA transport, inflammation, renal fibrosis and oxidative stress. Further studies with sophisticated research design can further demonstrate the efficacy and safety of these Chinese herbal medicines in lowering sUA levels. Identification of the active ingredients and delineation of the underlying mechanisms of action can facilitate the clinical translation and application of these ingredients.

## Author Contributions

All the authors contributed sufficiently for their participation in the study, as follows: XZ and LZ conceived, designed, and supervised the study; LC, ZL, MW, JC, FL, and HL conducted the literature search, data extraction, data analysis and interpretation; QH, YY, and XZ appraised the articles; LC, ZL, and MW wrote the paper, HK modified the paper. All authors have read and approved the final manuscript.

## Funding

This work was supported by the Key Project of National Natural Science Foundation of China (No. 81830117), the National Science Foundation of China (Nos. 81774212, 81760821, 81703952), the Natural Science Foundation of Guangdong Province, China (Nos. 2017A030313722, 2018A030313375, 2019A1515010400), and the Science & Technical Plan of Guangzhou, Guangdong, China (No.201903010069).

## Conflict of Interest

The authors declare that the research was conducted in the absence of any commercial or financial relationships that could be construed as a potential conflict of interest.

## Abbreviations

Abbreviations can be referred to Tables 3, 4. FABP1, fatty acid-binding protein; HPRT1, hypoxanthine-guanine phosphoribosyl transferase; APOB, apolipoprotein B; FOS, one subunit of activator protien-1; FN1, fibronectin1; MIP-1α, MIP-1 β, serum proinflammatory cytokines; TXNIP, thioredoxin interacting proteins; PTEN, phosphate and tension homology deleted on chromsome ten; NALP1/6, NACHT, LRR and PYD domains-containing protein one and six; ADAMTs, a family of metalloproteinases with thrombospondin motifs; TIMP, tissue inhibitor of metalloproteinase; CD2AP, CD2-associated protein.
