# Supplementary material for: The Efficacy and Mechanism of Chinese Herbal Medicines in Lowering Serum Uric Acid Levels: A Systematic Review
Source: Front Pharmacol. 2021 Jan 25;11:578318. doi: 10.3389/fphar.2020.578318 (PMC7868570; doi:10.3389/fphar.2020.578318)
Supplement: Supplementary file 1 [file table1.docx]

Supplementary Material

# Supplementary Figures and Tables

# Supplementary Figures

# Supplementary Figure S1


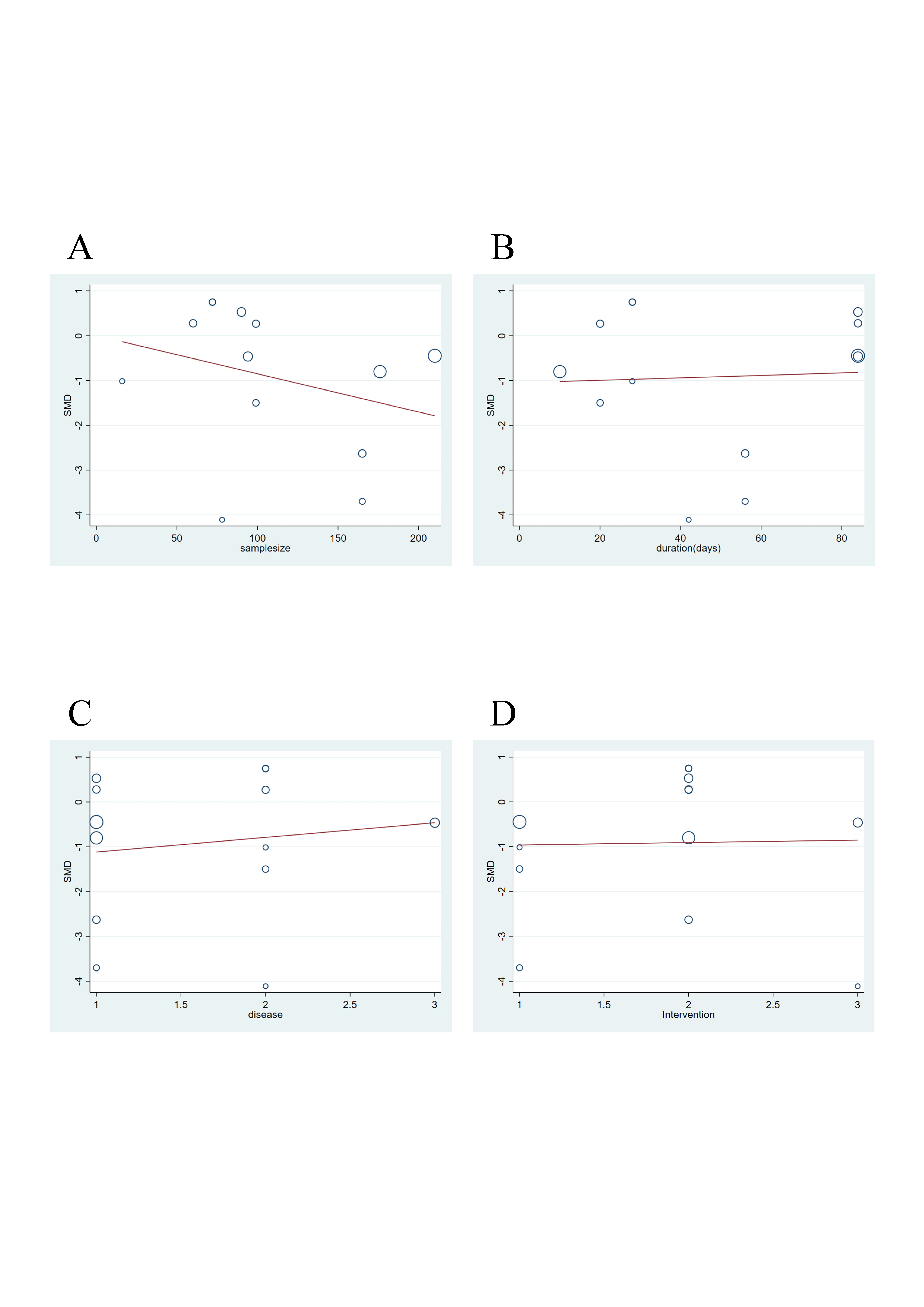


**Supplementary Figure S1.** Meta-regression analyses. A) Meta-regression analysis between SMD and the sample size. B) Meta-regression analysis between SMD and the duration of treatment. C) Meta-regression analysis between SMD and the types of diseases. D) Meta-regression analysis between SMD and the intervention other than Chinese herbal medicine.

# Supplementary Figure S2


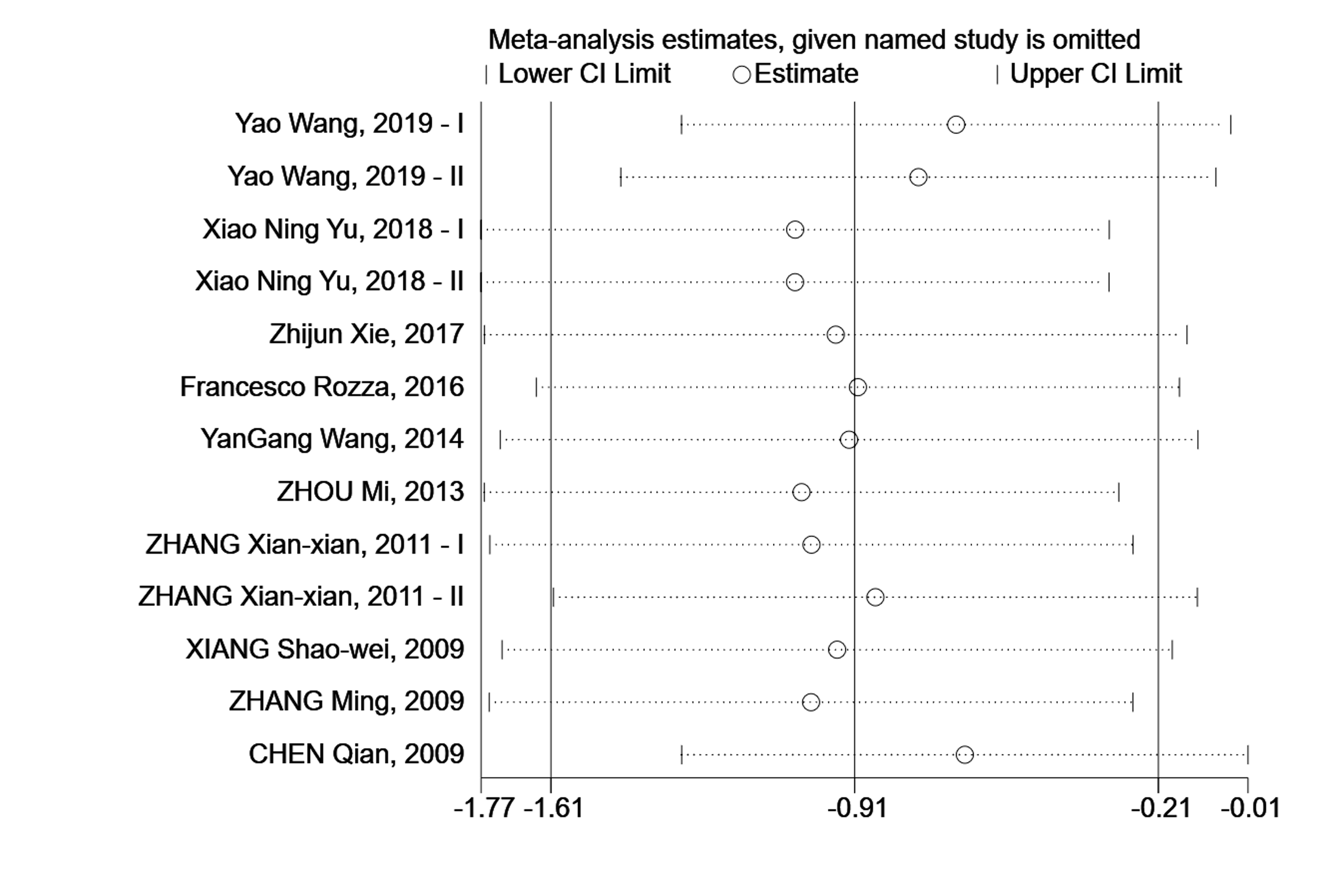


**Supplementary Figure S2.** Sensitivity analysis of the meta-analysis.

# Supplementary Figure S3

# Supplementary Figure S3. Publication bias of the meta-analysis. A) Begg’s funnel plot of the meta-analysis. B) Egger’s publication bias plot of the meta-analysis.


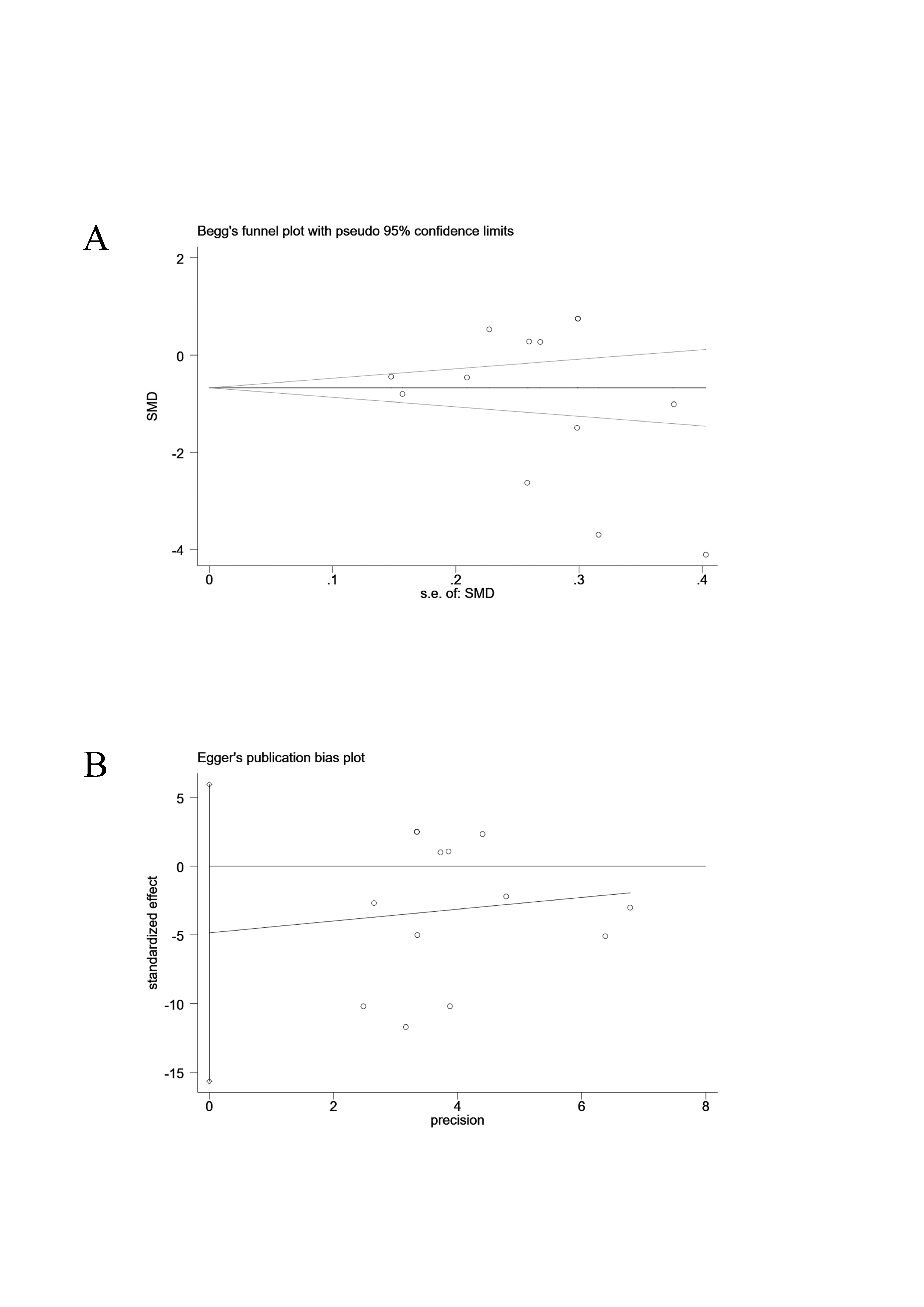


# Supplementary Tables

# Supplementary Table S1. PRISMA Checklist([Moher et al., 2009](#_ENREF_93))

| **Section/topic** | **#** | | | **Checklist item** | **Reported on page #** |
| --- | --- | --- | --- | --- | --- |
| **TITLE** | | | | |  |
| Title | 1 | | | Identify the report as a systematic review, meta-analysis, or both. | Page 1 |
| **ABSTRACT** | | | | |  |
| Structured summary | 2 | | | Provide a structured summary including, as applicable: background; objectives; data sources; study eligibility criteria, participants, and interventions; study appraisal and synthesis methods; results; limitations; conclusions and implications of key findings; systematic review registration number. | Page 1-2 |
| **INTRODUCTION** | | | | |  |
| Rationale | 3 | | | Describe the rationale for the review in the context of what is already known. | Page 2 |
| Objectives | 4 | | | Provide an explicit statement of questions being addressed with reference to participants, interventions, comparisons, outcomes, and study design (PICOS). | Page 2 |
| **METHODS** | | | | |  |
| Protocol and registration | 5 | Indicate if a review protocol exists, if and where it can be accessed (e.g., Web address), and, if available, provide registration information including registration number. | | | - |
| Eligibility criteria | 6 | Specify study characteristics (e.g., PICOS, length of follow-up) and report characteristics (e.g., years considered, language, publication status) used as criteria for eligibility, giving rationale. | | | Page 3-4 |
| Information sources | 7 | Describe all information sources (e.g., databases with dates of coverage, contact with study authors to identify additional studies) in the search and date last searched. | | | Page 4 |
| Search | 8 | Present full electronic search strategy for at least one database, including any limits used, such that it could be repeated. | | | Table S2 |
| Study selection | 9 | State the process for selecting studies (i.e., screening, eligibility, included in systematic review, and, if applicable, included in the meta-analysis). | | | Figure 1 |
| Data collection process | 10 | Describe method of data extraction from reports (e.g., piloted forms, independently, in duplicate) and any processes for obtaining and confirming data from investigators. | | | Page 3 |
| Data items | 11 | List and define all variables for which data were sought (e.g., PICOS, funding sources) and any assumptions and simplifications made. | | | Page 4 |
| Risk of bias in individual studies | 12 | Describe methods used for assessing risk of bias of individual studies (including specification of whether this was done at the study or outcome level), and how this information is to be used in any data synthesis. | | | Page 4 |
| Summary measures | 13 | State the principal summary measures (e.g., risk ratio, difference in means). | | | Page 4 |
| Synthesis of results | 14 | Describe the methods of handling data and combining results of studies, if done, including measures of consistency (e.g., I^2^) for each meta-analysis. | | | Page 4 |
| Risk of bias across studies | 15 | | Specify any assessment of risk of bias that may affect the cumulative evidence (e.g., publication bias, selective reporting within studies). | | Page 4 |
| Additional analyses | 16 | | Describe methods of additional analyses (e.g., sensitivity or subgroup analyses, meta-regression), if done, indicating which were pre-specified. | | Page 4 |
| **RESULTS** | | | | |  |
| Study selection | 17 | | Give numbers of studies screened, assessed for eligibility, and included in the review, with reasons for exclusions at each stage, ideally with a flow diagram. | | Page 4-5,  Figure 1 |
| Study characteristics | 18 | | For each study, present characteristics for which data were extracted (e.g., study size, PICOS, follow-up period) and provide the citations. | | Page 7, Table 1 |
| Risk of bias within studies | 19 | | Present data on risk of bias of each study and, if available, any outcome level assessment (see item 12). | | Table 2 |
| Results of individual studies | 20 | | For all outcomes considered (benefits or harms), present, for each study: (a) simple summary data for each intervention group (b) effect estimates and confidence intervals, ideally with a forest plot. | | Figure 2 |
| Synthesis of results | 21 | | Present results of each meta-analysis done, including confidence intervals and measures of consistency. | | Page 7-8, Figure 2 |
| Risk of bias across studies | 22 | | Present results of any assessment of risk of bias across studies (see Item 15). | | Table 2 |
| Additional analysis | 23 | | Give results of additional analyses, if done (e.g., sensitivity or subgroup analyses, meta-regression [see Item 16]). | | Page 8-9, Figure S1, Figure S2, Figure S3 |
| **DISCUSSION** | | | | |  |
| Summary of evidence | 24 | | Summarize the main findings including the strength of evidence for each main outcome; consider their relevance to key groups (e.g., healthcare providers, users, and policy makers). | | Page 9 - 10 |
| Limitations | 25 | | Discuss limitations at study and outcome level (e.g., risk of bias), and at review-level (e.g., incomplete retrieval of identified research, reporting bias). | | Page 9 - 11 |
| Conclusions | 26 | | Provide a general interpretation of the results in the context of other evidence, and implications for future research. | | Page 9-11, Page 16 |
| **FUNDING** | | | | |  |
| Funding | 27 | | Describe sources of funding for the systematic review and other support (e.g., supply of data); role of funders for the systematic review. | | Page 16 |

# Supplementary Tables S2. Search strategies and the number of records according to different electronic database.

| **Search strategy** | **Database** | **Num. of records** |
| --- | --- | --- |
| (((((((((((((((((((((((Herbal Medicine[MeSH]) OR (Herbal Medicine)) OR (Drugs, Chinese Herbal[MeSH])) OR (Drugs, Chinese Herbal)) OR (Chinese Herbal Drugs)) OR (Chinese Drugs, Plant)) OR (Chinese Plant Extracts)) OR (Plants, Medicinal)) OR (Plants, Medicinal[MeSH])) OR (Pharmaceutical Plant)) OR (Healing Plant)) OR (Medicinal Herb)) OR (herb*)) OR (Phytochemicals[MeSH])) OR (Phytochemical)) OR (Plant Bioactive Compounds)) OR (Plant Derived Compounds)) OR (Plant Derived Chemicals)) OR (Phytomedicine)) OR (natural product)) ) OR (traditional Chinese medicine[MeSH])) OR (traditional Chinese medicine)) AND ((((((((Gout) OR Pain Paralysis) OR Hyperuricemia[MeSH]) OR Hyperuricemia) OR Uric Acid[MeSH]) OR Uric Acid))) | PubMed | 1460 |
|  | Web of Science | 1249 |
|  | Cochrane Library | 246 |
|  | Embase | 2026 |

# Supplementary Tables S3. Changes of serum uric acid before and after treatment in clinical trials.

| **Compounds/formula** | **sUA (μmmol/L)** | | **p-value** | **Baseline difference** | **Reference** |
| --- | --- | --- | --- | --- | --- |
|  | **BT** | **AT** |  |  |  |
| Chuanhutongfeng Mixture | T: 530.00 ± 74.00 | T: decrease sUA: 170.00 ± 49.00 | < 0.05 | NSD | ([Wang et al., 2019b](#_ENREF_129)) |
|  | C: 529.00 ± 72.00 | C: decrease sUA: 70.00 ± 21.00 |  |  |  |
|  | Placebo: 532.00 ± 69.00 | Placebo: decrease sUA: 26.00 ± 23.00 |  |  |  |
| Yellow-dragon Wonderful-seed Formula | T: 562.29 ± 108.30 | T: 526.29 ± 156.15 | NSD | NSD | ([Yu et al., 2018](#_ENREF_172)) |
|  | C: 618.00 ± 114.27 | C: 480.83 ± 144.34 |  |  |  |
| Yellow-dragon Wonderful-seed Formula+Gypsum Fibrosum | T: 585.46 ± 100.06 | T: 566.29 ± 206.08 | NSD | NSD | ([Yu et al., 2018](#_ENREF_172)) |
|  | C: 618.00 ± 114.27 | C: 480.83 ± 144.34 |  |  |  |
| Compound tufuling oral-liquid | T: 550.00 (103.54) | T: decrease sUA: 75.34(95%CI: 57.80-92.88) | N/A | NSD | ([Xie et al., 2017](#_ENREF_152)) |
|  | C: 530.00 (105.00) | C: decrease sUA: 28.81 (95%CI: 4.91-52.71) |  |  |  |
| ZinutriK | T: 470.05 ± 53.55^a^ | T: 416.50 ± 41.65^a^ | 0.006 | NSD | ([Rozza et al., 2016](#_ENREF_103)) |
|  | C: 470.05 ± 53.55^a^ | C: 470.05 ± 59.50^a^ |  |  |  |
| The Chuanhu anti-gout mixture | T: 465.22 ± 79.88 | T: decrease sUA: 63.17 ± 42.06 | < 0.001 | N/A | ([Wang et al., 2014](#_ENREF_133)) |
|  | C: 449.81 ± 68.16 | C: decrease sUA: 29.00±43.38 |  |  |  |
| A series of tongfeng granule | T: 527.60 ± 77.50 | T: 448.20 ± 71.80 | < 0.05 | NSD | ([Zhou et al., 2013](#_ENREF_187)) |
|  | C: 533.10 ± 71.60 | C: 415.40 ± 59.40 |  |  |  |
| Xiezhuo Chubi Recipe | T: 508.00 ± 89.46 | T: 386.57 ± 69.60 | < 0.05 | NSD | ([Zhang et al., 2011](#_ENREF_181)) |
|  | C: 493.71 ± 65.45 | C: 352.21 ± 66.79 |  |  |  |
|  | No intervention: 494.20 ± 45.86 | No intervention: 469.90 ± 42.79 |  |  |  |
| Retention Enema of Chinese Herbal Medicine | T: 483.80 ± 32.29 | T: 287.12 ± 23.78 | < 0.01 | NSD | ([Chen et al., 2009](#_ENREF_12)) |
|  | C: 476.80 ± 33.75 | C: 400.46 ± 21.63 |  |  |  |
| Modified Sanmiao Powder | T: 602.69 ± 127.64 | T: 411.04 ± 90.38 | < 0.01 | NSD | ([Xiang et al., 2009](#_ENREF_151)) |
|  | C: 587.74 ± 131.87 | C: 448.83 ± 83.25 |  |  |  |
| Serial Gout Granules | T: 527.83 ± 100.50 | T: 466.70 ± 60.55 | < 0.05 | NSD | ([Zhang et al., 2009](#_ENREF_177)) |
|  | C: 547.47 ± 87.24 | C: 463.47 ± 46.29 |  |  |  |

Data are presented a mean ± SD or a median (QR) for continuous variables. ^a^Data converted by units of measurement. P-value: differences in treatment group before and after treating.

sUA, serum uric acid; BT, before treatment; AT, after treatment; C, control group; T, treatment group; NSD, no significant difference; N/A: not available.

# Supplementary Tables S4. Detailed information on botanical in the included studies.

| Study | Species, source, concentration | Quality control reported? (Y/N) | Chemical analysis reported? (Y/N) |
| --- | --- | --- | --- |
| ([Chen et al., 2020b](#_ENREF_13)) | - *Clerodendranthus spicatus,* [The herb market of Juhua county, Kunming, China], 15kg | Y-Identified by Dr. W. Fang from Kunming Institute of Botany. A voucher specimen (Luo 20181115) was deposited in Kunming Institute of Botany, Chinese Academy of Sciences. Extraction and Isolation. | Y-HPLC |
| ([Guo et al., 2020](#_ENREF_30)) | - *Atractylodes* - *Cork* - *Coix seed* - *Achyranthes* - *Dendrobium officinalis* | N | N |
| ([Lin et al., 2020](#_ENREF_69)) | - *Atractylodes lancea* (Thunb.), DC 12g - *Phellodendron amurense Rupr.*, 12g - *Achyranthes bidentata Blum,*12g - *Coix lacryma-jobi var. ma-yuen(Rom.Caill.) Stapf,* 30g - [Zhejiang Chinese Medical University Medicine Yinpian Factory (Hangzhou,China)] | Y - Identified by Prof. Chengping Wen and Dr. Lin Huang. The voucher specimen (No. 20190128-1 for Atractylodes lancea (Thunb.) DC., No. 20190128-2 for Phellodendron amurenseRupr., No. 20190128-3 for Achyranthes bidentata Blume., No. 20190128-4 for Coix lacryma-jobi var. ma-yuen (Rom.Caill.) Stapf) have been deposited by Zhixing He at the Institute of Basic Research in Clinical Medicine of Zhejiang Chinese Medical University (Hangzhou, China). | Y - UPLC |
| ([Liu et al., 2020](#_ENREF_71)) | - The sunflower head enzymatic hydrolysate, [Chengdu Yikangtang Pharmaceutical Co. Ltd (Chengdu, China)] | N | N |
| ([Lou et al., 2020](#_ENREF_80)) | - Dendrobium candidum leaves, [Yunnan Alpine Agriculture Co., Ltd (Yunnan, China)] | N | N |
| ([Pan et al., 2020](#_ENREF_98)) | - Liriodendron chinense (Hemsl.) Sarg barks, 500g - [Qionglai City, Chengdu, Sichuan Province, China] | Y - A voucher specimen (EELC-201712) was characterized by Dr. Hao-Yu Ye of State Key Laboratory of Biotherapy, Sichuan University, Chengdu, China and deposited in the Kidney Research Institute, West China Hospital of Sichuan University, Chengdu, China. | Y - UPLC |
| ([Yao et al., 2020](#_ENREF_164)) | - *Polygonum cuspidatum Sieb.et Zucc,* - *Sargentodoxa cuneate(Oliv.)Rehd.et Wils* - *Smilax glabra Roxb.* - *Lonicera japonica ;unb.* - *Lysimachia christinae Hance.* - *Phellodendron chinense Schneid.* - *Commiphora myrrha Engl.* - *Angelica dahurica(Fisch.ex Hoffm.) Benth. et Hook. F* - [Anguo Herb Market (China).] | Y - Identified by Dr. Xirong He and complied with the specification of Pharmacopoeia of the People’s Republic of China (2015). | N |
| ([Zuo et al., 2020](#_ENREF_197)) | - *Viola yedoensis* Makino (Viola) - *Taraxacum mongolicum* Hand.-Mazz. (Taraxacum) - *Lobelia chinensis* Lour.(Lobelia) - Root of *Isatis indigotica* Fort. - [Jiangxi Provincial Hospital of Traditional Chinese Medicine, Nanchang, China.] - Ratio = 1:1:1:1 | Y - The voucher specimens of the four herb materials were deposited at the Herbarium of the Jiangxi University of Traditional Chinese Medicine with the number 150705. | Y - HPLC |
| ([Chen et al., 2019](#_ENREF_7)) | - *Tinospora sinensis* - *Terminalia chebula Retz* - *Trogopterori faeces* - [herbal medicine markets across Hehua City (Chengdu Province, China).] - Ratio = 5:4:2 | N | Y - HPLC |
| ([Cheng et al., 2019](#_ENREF_20)) | - *Alismatis Rhizoma* [Sichuan Guoqiang Chinese Herbal Pieces Factory] - *Rhizoma Smilacis Glabrae* [Sichuan Guoqiang Chinese Herbal Pieces Factory] | N | N |
| ([Fang et al., 2019](#_ENREF_28)) | - The cortex of *Eucommia ulmoides*, [Tian Qi Tang pharmacy (Zhangshu China)], 505.73g | Y - Identified by Bei Wang, the Vice Director of Pharmacists from the Nanchang Institute for Drug Control (Nanchang, China). Voucher specimens were deposited at the laboratory of the Jiangxi University of Traditional Chinese Medicine. | N |
| ([Huang et al., 2019a](#_ENREF_44)) | - *Phellodendri Chinensis* Cortex, [Nanjing Tongrentang Pharmaceutical Co., Ltd. (Nanjing, China)], 1000g - *Atractylodis Rhizoma*, [Nanjing Tongrentang Pharmaceutical Co., Ltd. (Nanjing, China)], 1000g | Y - Authenticated by Dr. Jun Chen (China Pharmaceutical University). | Y - UPLC-Q-TOF/MS |
| ([Huang et al., 2019b](#_ENREF_46)) | - The rhizomes of *Smilax glabra* [Zhangshu County, Jiangxi Province, China] | Y - A voucher specimen was deposited at the Key Laboratory of Modern Preparation of traditional Chinese medicine (TCM), Jiangxi University of TCM (Nanchang, China). | Y - HPLC |
| ([Liang et al., 2019](#_ENREF_68)) | - *Rhizoma Smilacis Glabrae* Extracts [Kangmei Pharmaceutical Co. Ltd. (Puning, China)] | Y - Identified by Dr. Wei Zou (Department of Pharmacy, Hunan Province Maternal and Child Health Hospital) | Y - HPLC–DAD-MS/MS |
| ([Liu et al., 2019](#_ENREF_74)) | - Extract of shelled fruits of *O. Sativa* [Yunnan, China] | N | Y - HPLC |
| ([Ma et al., 2019a](#_ENREF_89)) | - Ethanolic extract of *Polygonum cuspidatum* [Zhejiang Chinese Medical University Medical Pieces., Ltd] | Y - Prepared according to Chinese Pharmacopeia (2015 Edition) | Y - HPLC |
| ([Ma et al., 2019b](#_ENREF_90)) | - *Polygonum cuspidatum* [Department of Pharmacy, Dongfang Hospital, Beijing University of Chinese Medicine] | N | N |
| ([Peng et al., 2019](#_ENREF_101)) | - Chrysanthemum extract | N | N |
| ([Wang et al., 2019a](#_ENREF_125)) | - The rhizome of *Smilax glabra Roxb*., [Zhejiang Chinese Medicine University Pieces Factory] | N | N |
| ([Wang et al., 2019b](#_ENREF_129)) | - *Rhizome dioscorea nipponica* (15g), - *Rhizoma polygoni cuspidati* (15 g), - *Caulis lonicerae* (30 g), - *Radix saposhnikoviae* (15 g), - *Radix clematidis* (15 g), - *Rhizoma smilacis glabrae* (15 g), - *Radix cyathulae* (15 g), - *Rhizoma ligustici chuanxiong* (15 g), - *Rhizoma dioscoreae hypoglaucae* (12 g), - *Fructus chaenomelis* (15 g), - *Radix glycyrrhizae* (6 g), - sodium alginate (1 g). | N | Y |
| ([Wang et al., 2019c](#_ENREF_131)) | - Chicory (*Cichorium intybus L*.) | Y - Authenticated by Professor Yonghong Yan (Beijing University of Chinese Medicine). | N |
| ([Yang et al., 2019a](#_ENREF_157)) | - *Desmodium styracifolium* - *Ventriculi galli mucosa* - *Alisma orientalis* - *Sand cattle* - *Astragalus* - *Plantago seed* - *Corydalis corydalis* - Licorice - [(Qingdao Gla Dandong Pharm Co., Ltd., Ge’ermu, China)] | Y - National Medicine Permission  Drug | N |
| ([Yang et al., 2019c](#_ENREF_160)) | - The aerial parts of *Terminthia paniculata* (Wall. ex G. Don) C.Y. Wu &T.L. Ming (Anacardiaceae), [Shidixin Village of Yuanjiang County, Yunnan Province, China], | Y - Authenticated by Prof. Hua Peng (Kunming Institute of  Botany, CAS). | Y - HPLC |
| ([You et al., 2019](#_ENREF_171)) | - *Cassia twig*, 10g - *D. nipponica*, 0.3g - *P. Cuspidatum,* 15g - *Honeysuckle rattan*, 30g - *Radix cyathulae,* 15g - *Glabrous greenbrier*, 15g - *Radix saposhnikoviae,* 15g - *Radix clematidis*, 15g - *Rhizoma chuanxiong*, 15g - *Coix seed,* 15g - *Glycyrrhizae radix et rhizoma*, 6g - *Sodium alginate,* 2g - [Qingdao Huanghai Pharmaceutical (Qingdao, China).] | N | N |
| ([Yuan et al., 2019](#_ENREF_173)) | - N/A | N | N |
| ([Zhang et al., 2019a](#_ENREF_180)) | - *Viola yedoensis Makino*, [Jiangxi Zhangshu Tianqitang Traditional Chinese Medicine Pieces Co. Ltd. (Zhangshu, China)], 100g - *Taraxacum mongolicum* Hand.-Mazz., [Jiangxi Zhangshu Tianqitang Traditional Chinese Medicine Pieces Co. Ltd. (Zhangshu, China)], 100g - *Lobelia chinensi Lour.*, [Jiangxi Zhangshu Tianqitang Traditional Chinese Medicine Pieces Co. Ltd. (Zhangshu, China)], 100g - *Isatis indigotica Fort*., [Jiangxi Zhangshu Tianqitang Traditional Chinese Medicine Pieces Co. Ltd. (Zhangshu, China)], 100g | Y - stored in the National Engineering Research Center of Traditional Chinese Medicine Solid Preparation Manufacturing Technology, Jiangxi University of Traditional Chinese Medicine (Jiangxi, China). | Y - UHPLC-Q-TOF-MS/MS Analysis |
| ([Zhang et al., 2019b](#_ENREF_182)) | - *S. moellendorffii Herba* (SM) - *Smilacis glabrae Rhizoma*(SGR) - *Plantaginis Semen* (PS) - [Jointown Pharmaceutical Group Co., Ltd., China.] | Y-Authenticated by authors (Prof. Ke-li Chen). Specimens of these materials were deposited in the herbarium, Hubei University of Chinese Medicine, China | Y-HPLC |
| ([Zhang et al., 2019c](#_ENREF_185)) | - The dried purple sweet potato powder (Ipomoea batatas L. cultivar Eshu No. 8) [the Puzetian Food Co. Limited (Wuhan, Hubei, China).] | N | Y - HPLC |
| ([Cho et al., 2018](#_ENREF_21)) | - Leaves of *Dendropanax morbifera* (Aralicaceae), [Jeollanamdo Wando provincial government in Jeonnam, Korea] | Y - A voucher specimen (MNUCSS-DM-02) was deposited in the Mokpo National University (Muan, Korea). | Y - HPLC |
| ([Lu et al., 2018](#_ENREF_83)) | - *Codonopsis tangshen* Oliv. - [Shennongjia forestry region of Hubei Province] | Y - Authenticated by Dr. Jingyu He (Guangzhou Institute of Advanced Technology, Chinese Academy of Sciences, Guangzhou, China). | Y |
| ([Song et al., 2018](#_ENREF_112)) | - Leaf of *Cudrania tricuspidata* [Jeonnam Forest Resource Institute (Naju, South Korea)], 100 g | Y - Identified by Dr. Deuk-Sil Oh affiliated to the Jeonnam Forest Resource Institute | Y - HPLC |
| ([Su et al., 2018](#_ENREF_114)) | - Dried Polyrhachis vicina Rogers, 1000g - [Nanning city (Guangxi, China)] | Y - The voucher specimen (PR-201505) was identified by Professor Xianbiao Zeng from Guangxi Institute of Chinese Medicine & Pharmaceutical Science and was deposited in the Herbarium of Guangxi Institute of Chinese Medicine & Pharmaceutical Science. | Y - GC-MS |
| ([Tian et al., 2018](#_ENREF_117)) | - *Radix Astragali* - *Fructus Corni,* - *Rhizoma Coptidis* - *Radix Puerariae Lobatae* - *Cortex Mori* - *Herba Eupatorii* - [Tongrentang Pharmacy Branch (Tangshan,China)] - Rate = 5:5:4:4:3:2 | N | N |
| ([Wei et al., 2018](#_ENREF_138)) | - *Phellodendri cortex*, [Tongrentang Pharmacy (Changchun, China)] - *Atractylodis rhizome*, [Tongrentang Pharmacy (Changchun, China)] - Ratio = 1:1 | Y - Authenticated by Professor Shumin Wang (Changchun University of Traditional Chinese Medicine, China) | N |
| ([Wu et al., 2018a](#_ENREF_140)) | - *Rhizoma Smilacis Glabrae*, 35g - *Rhizoma Dioscoreae Hypoglaucae*,18g - *Pseudobulbus Cremastrae Seu Pleiones,*15g - *Semen Vaccariae*, 10g - *Radix Achyranthis Bidentatae*,10g - [Department of Pharmacy of the Guangzhou Military Command General Hospital] | N | Y - HPLC |
| ([Wu et al., 2018b](#_ENREF_149)) | - Vaccaria tablets, 30 g - white mustard seed tablets, 30 g - abutilon seed tablets, 30 g - plantago seed tablets, 60 g - [Shanghai Union Dispensary Co.] | N | Y - HPLC |
| ([Xiong et al., 2018](#_ENREF_153)) | - *Lagotis brachystachys Maxim* [Chengdu Chinese medicinal materials Market], 100g | Y-Identified by Zhong Guoyue, researcher of Jiangxi University of Traditional Chinese Medicine | N |
| ([Yu et al., 2018](#_ENREF_172)) | - Pheretima, [Sichuan Rejuvenation Hall Pharmaceutical Co., Ltd., China.], 10g - *Amomum kravanh Pirre ex Gagnep*, [Sichuan Rejuvenation Hall Pharmaceutical Co., Ltd., China.], 6g - *Cortex Phellodendri Chinensis*, [Sichuan Rejuvenation Hall Pharmaceutical Co., Ltd., China.], 10g - *Atractylodes Lancea (Thunb.) DC.*, [Sichuan Rejuvenation Hall Pharmaceutical Co., Ltd., China.], 9g - *Coix lacryma-jobi L.var.ma-yuen (Roman.)Stapf(Yi Yi)*, [Sichuan Rejuvenation Hall Pharmaceutical Co., Ltd., China.], 20g - *Cyathula officinalis Kuan.*, [Sichuan Rejuvenation Hall Pharmaceutical Co., Ltd., China.], 10g | Y - Prepared according to Chinese Pharmacopeia (2015 Edition) | Y |
| ([Yuk et al., 2018](#_ENREF_174)) | - Leaves of *Toona Sinensis* [tree communities in Sacheon, South Korea], 200 g | Y - A voucher specimen (KRIBB 0000583) was deposited in the Korea Research Institute of Bioscience and Biotechnology | Y - UPLC-qToF MS |
| ([Zhang et al., 2018a](#_ENREF_178)) | - The fruits of *Chaenomeles sinensis* (Thouin) Koehne, [Yaorong papaya Bio-Tech Development Co., Ltd (Hubei, China)], 5g | N | Y - HPLC |
| ([Zhou et al., 2018](#_ENREF_191)) | - Fraxini Cortex, [Shangluo, Shaanxi Province (batch number 20160507), Pingdingshan, Henan Province (batch number 20160412), Anguo, Hebei Province (batch number 20160502)], 300g | Y - Identified by Dr. G. Zhang from Shaanxi University of Chinese Medicine. | Y - HPLC |
| ([Zuo et al., 2018](#_ENREF_196)) | - Whole plants of Viola yedoensis Makino (Viola, lot: 150309), - Whole plants of Taraxacum mongolicum Hand. Mazz. (Taraxacum, lot: 150420), - Whole plants of Lobelia chinensis Lour. (Lobelia, lot: 150417) - Isatis indigotica Fort. roots (Isatidis, lot: 150317) - [Jiangxi Provincial Hospital of Traditional Chinese medicine, Nanchang, China] | Y - Prepared according to Chinese Pharmacopeia (2015 Edition) | Y - HPLC |
| ([Chen et al., 2017a](#_ENREF_14)) | - *Selaginella tamariscina*, [Jilin Pharmacy (Changchun, China)], 500g | N | N |
| ([Han et al., 2017](#_ENREF_33)) | - *Rhizoma Polygoni Cuspidati* (lot No. 130701), [Guangdong Hexiang Pharmaceutical Co. Ltd., China] - *Ramulus Cinnamomi,* (lot No. 28113011), [Guangdong Province Medicine Company Herbal Pieces Factory, China] | N | N |
| ([Kim et al., 2017](#_ENREF_54)) | - The dried whole plant of *Salvia Plebeia* R. BR. (Labiatae), [Gyeonggi Province, Korea in 2015], 100g | Y - Authenticated by Dr. Jin Kyu Kim. A voucher specimen (voucher No.: G58) was deposited in the herbarium at the Gyeonggi Institute of Science and Technology Promotion. | Y - UHPLC‑HR MS/MS |
| ([Lee et al., 2017](#_ENREF_62)) | - *Mollugo pentaphylla*, [Yangpyung, Kyounggi-do], 1000g | Y - Confirmed taxonomically by Dr. Geung-Joo Lee of the Chungnam National University. A voucher specimen (no. KIOM201701018962) was deposited at the Korean herbarium of Standard Herbal Resources at the KIOM. | N |
| ([Li et al., 2017a](#_ENREF_64)) | - Sunflower head powders, [Baicheng, Jilin, China, in October 2015], 10g | N | Y |
| ([Li et al., 2017b](#_ENREF_65)) | - Dried and pulverized stems of *Aristolochia bracteolata* | N | N |
| ([Wang et al., 2017a](#_ENREF_130)) | - Chicory | Y - Authenticated by Professor  Yong-Hong Yan (Traditional Chinese Medicine Appraisal Teaching and Research Section of Beijing University of Chinese Medicine) | Y - HPLC |
| ([Wang et al., 2017b](#_ENREF_132)) | - Cichorii Herba | Y | N |
| ([Wang et al., 2017c](#_ENREF_135)) | - Cortex Fraxini, [Guoda pharmacy (Shenyang, China)], 200g | Y - Identified by Professor Jingming Jia (Department of TCM, Shenyang Pharmaceutical University, Shenyang, China). A voucher specimen was deposited at the College of Traditional Chinese Medicine, Shenyang Pharmaceutical University. | Y - HPLC |
| ([Wu et al., 2017](#_ENREF_148)) | - Vaccaria tablets (30 g; batch number 140220) - White mustard seed tablets (30 g; batch number LY1505016) - Abutilon seed tablets (30 g; batch number 160517HY) - Plantago seed tablets (60 g; batch number 151225) - [Shanghai Chinese Traditional Pharmaceutical Technology Co., Ltd. (China)] | N | Y - LC–MS |
| ([Xia et al., 2017](#_ENREF_150)) | - *Plantago depress Willd,* [Guilin City, Guangxi Province in China) | Y - Identified by Professor Li He of Hubei University of Arts and Science in China. A voucher specimen (no. PDWE 20150922) was deposited in the Herbarium of College of Pharmacy, Hubei University of Arts and Science, China. | N |
| ([Yong et al., 2017](#_ENREF_166)) | - *Ganoderma applanatum*, [Guangdong Yuewei Edible Fungi Technology Co. (Guangzhou, China)], 100 g | Y - A voucher specimen (YW20170517-GA) was deposited in the herbarium of Guangdong Institute of Microbiology. | Y - HPLC |
| ([Yoon et al., 2017a](#_ENREF_168)) | - The leaves of *Quercus acuta* Thunb. (Fagaceae), [Wando Arboretum (Wando, Republic of Korea)], 10g | Y - Identified by Dr. Deuk-Sil Oh affiliated to the Wando Arboretum. A voucher specimen (MNUCSS-QA-01) was deposited at Mokpo National University (Muan, Republic of Korea) | Y - GC‑MS |
| ([Yoon et al., 2017b](#_ENREF_170)) | - The leaves of *Camellia japonica* L. (*C. japonica*), [Jeollanamdo Wando Arboretum, in Jeonnam, Korea], 10g | Y - A voucher specimen (MNUCSS-CJ-01) was deposited at Mokpo National University (Muan, Korea). | Y - GC‑MS |
| ([Zhang et al., 2017a](#_ENREF_176)) | - *Gnaphalium Affine* D. Don, [Yuhuan by Xi-biao Zhang, Zhejiang province, PR China in November 2015], 500g | Y - Identified by Licensed Pharmacist Yi-bo Feng, Tongde Hospital of Zhejiang Province and the voucher specimens (SQC20151101) was deposited at 139 the Key Laboratory of Research and Development of Chinese Medicine140 of Zhejiang Province, Zhejiang Academy of Traditional Chinese Medicine. | Y - HPLC-QTOF-MS |
| ([Zhang et al., 2017b](#_ENREF_179)) | - Ginkgo folium, [Beijing Sanhe pharmaceutical co., LTD], 100g | N | N |
| ([Zhao et al., 2017](#_ENREF_186)) | - *Selaginella moellendorffii,* [Yi Chang in Hubei province, China], 2kg | Y - Identified by author. Specimens of these plants were deposited in the herbarium, Hubei University of Chinese Medicine, China. | Y - HPLC |
| ([Chen et al., 2016a](#_ENREF_8)) | - *Glabrous Greenbrier Rhizome 60g* - *Dioscorea septemloba Thunb 30g* - *Maydis stigma 15g* - *coix seed 30g* - *Alismatis rhizome 15g* - *Humulus scandens 15g* - *Parasiticloranthus 15g* - *Herba Siegesbeckiae 18g* - *turmeric 12g* - *CorydalisRhizoma 18g* - *Citrus medica 12g* - [Medical Pieces Co., Ltd., ofZhejiang Chinese Medical University (Hangzhou, Zhejiang, China).] | N | N |
| ([Fei et al., 2016](#_ENREF_29)) | - A whole plant of *Dioscorea tokoro Makino*, [Bozhou City, Anhui Province in China] | Y - Authenticated by Professor HeHuang of Zhejiang University in China. A voucher specimen (no. DTME 20150517) was deposited in the Herbarium of College of Pharmacy, Zhejiang University, China for future reference. | N |
| ([Han et al., 2016](#_ENREF_34)) | - *Radix rehmanniae praeparata*, 20g - *Cornus officinalis*, 20g - *Poria cocos*, 20g - *Cortex moutan*, 15g - *Rhizoma dioscoreae*, 20g - *Rhizoma alismatis*, 20g - *Amomum villosum*, 10g - *Achyranthes bidentatae*, 15g - *Semen plantaginis*, 30g - *Rhizoma smilacis glabrae*, 30g - *Dioscorea septemloba*, 20g - [Chinese Pharmacy at the First Affiliated Hospital of Heilongjiang University of Chinese Medicine] | N | N |
| ([Jhang et al., 2016](#_ENREF_50)) | - Dried *Mesona procumbens* Hemsl., [a local farmer (Hsinchu, Taiwan).], 10g | N | Y |
| ([Kou et al., 2016](#_ENREF_58)) | - *Olibanum* - *Caulis Tinosporae Sinesis* - *Semen Cassiae Obtusifoliae* - *Brag zhun* - *Semen Abelmoschi* - *Bhizoma Acori Calami* - *Adhatoda vasica Nees* - *Catechu* - *Fructus Chebulae* - *Benzoinum* - *Fructus Terminaliae Billerica* - *Radix Aconiti Penduli* - *Radix Aucklandiae* - *Moschus* - *Fructus Phyllanthi Emblicae* - [Qinghai Provincial Tibetan Medical Hospital, the authority in the area on Tibetan medicine] | N | N |
| ([Sheu et al., 2016](#_ENREF_106)) | - The male flowers, pericarps, seeds, leaves, and twigs of *Dimocarpus longan* Lour., [private farms in Hongjia (Xuejia District, Tainan City, Taiwan)] | N | Y |
| ([Shi et al., 2016a](#_ENREF_108)) | - *Atractylodes chinensis* (DC.) Koidz. - *Phellodendron chinense* Schneid., - *Coix lacryma-jobi* L. var.mayuen (Roman.)Stapf. - *Achyranthes bidentata* Bl. - *Smilax glabra* Roxb. - Lonicerajaponica Thunb. - [Department of Chinese Medicine Chemistry, Nanjing University of Chinese Medicine] | N | N |
| ([Shi et al., 2016b](#_ENREF_109)) | - Miracle Fruit (*Synsepalum dulcificum*), 25g | N | N |
| ([Wang et al., 2016a](#_ENREF_121)) | - *Dendropanaxchevalieri,* [Jiujiang, Jiangxi] | Y - Authenticated by Professor Zhang Shouwen, Department of Traditional Chinese Medicine Resources, Jiangxi University of Traditional Chinese Medicine | N |
| ([Wang et al., 2016b](#_ENREF_124)) | - Angelica sinensis radix (*Angelica sinensis* (Oliv.) Diels), 8g - Chuanxiong rhizome (*Ligusticum chuanxiong* Hort.), 8g - Paeoniae radix alba (*Paeonia lactiflora* Pall.), 4g - Rehmanniae radix praeparata (*Rehmannia glutinosa Libosch*.), 8g - [Jiangyin Tianjiang Pharmaceutical Co., Ltd. (Jiangyin, China)] | N | Y - UPLC-MS |
| ([Wang et al., 2016c](#_ENREF_126)) | - Mature leaves of *Tradescantia albiflora* (TA) Kunth (*Commelinaceae*), 100g | Y - Identified and authenticated by the botanist and a voucher specimen (TAIF-PLANT-199332) has been retained at the Herbarium of Taiwan Forestry Research Institute, Taipei, Taiwan | Y |
| ([Wang et al., 2016d](#_ENREF_128)) | - Rhizoma Smilacis Glabrae - Plantago asiatica L. - Kudzu root - Cichorium intybus L. - Seeds of Coix lacryma-jobi L. - Alismatis Rhizoma | N | N |
| ([Wang et al., 2016e](#_ENREF_134)) | - Cortex Fraxini, [(batch number：140501; source: Shanxi China), Guoda pharmacy (Shenyang, China)] | Y - identified by Professor Jingming Jia (Department of TCM, Shenyang Pharmaceutical University, Shenyang, China). A voucher specimen (No. 2015-1011) was deposited at the College of Traditional Chinese Medicine, Shenyang Pharmaceutical University. | Y - HPLC-FT-ICR-MS |
| ([Yong et al., 2016](#_ENREF_167)) | - Dried fruiting body (100 g) of *Cordyceps Militaris*, [Guangdong Yuewei Edible Fungi Technology Co. (Guangzhou, China)], 100g | Y - Voucher specimen was deposited in the herbarium of Guangdong Institute of Microbiology (YW20140825-CM). | Y - HPLC |
| ([Yoon et al., 2016](#_ENREF_169)) | - Flowers of *Corylopsis coreana* Uyeki (Hamamelidaceae) flos, [the Jogye Mountain, in Jeonnam Province, Korea], 10g | Y - A voucher specimen (MNUCSS-CC-01) was deposited in the Mokpo National University (Muan, Republic of Korea). | Y |
| ([Zhu et al., 2016](#_ENREF_193)) | - *Phellodendri chinensis Schneid.,* (90 g) - *A. chinensis (DC.)* Koidz., (45 g) - *A. bidentata* Bl., (45 g) | N | N |
| ([Amat et al., 2015](#_ENREF_2)) | - Seed of *Apium graveolent*, 30g - Root of *Apium graveolent*, 30g - Seed of *Cuscuta chinensis*, 20g - Seed of *Cichorium glandulosum*, 15g - Root of *Foeniculum vulgare*, 30g - Root of *Cichorium glandulosum*, 15g - [Xinjiang Autonomous Region Traditional Uighur Medicine Hospital (Urumqi, China)] | Y - Authenticated by associate chief pharmacist Anwar Talip. The voucher specimens (NU-110108, NU-100908, NU-110123, NU-110113, NU-110128, NU-100111) have been deposited in the Xinjiang Autonomous Region Traditional Uighur Medicine Hospital (Urumqi, China). | N |
| ([Chen et al., 2015b](#_ENREF_15)) | - Atractylodes lancea - Cortex phellodendri - [Beijing Tongrentang Pharmaceutical Co., Ltd] - Ratio = 1:1/1:2/2:1 | N | N |
| ([Guo et al., 2015](#_ENREF_31)) | - Si-Wu-Tang: - Rehmannia glutinosa,12 g - Angelica sinensis, 10 - Radix Paeoniae Alba, 12g, - Rhizoma Chuanxiong,8 g - Er-Miao-San: - Cortex Phellodendri Chinensis, 15 g, - of Atractylodes Lancea., 15 g - [Chinese Herbal Medicine Dispensary of Shanghai East Hospital.] | N | N |
| ([Huo et al., 2015](#_ENREF_49)) | - Leaves of *Perilla frutescens*, [Changchun suburb, Jilin Province of China], 3000g | Y - Authenticated by Bao-Min Feng, College of Life Science and Technology, Dalian University | Y - HPLC |
| ([Kodithuwakku et al., 2015](#_ENREF_55)) | - *Phragmites communis* Trin., - *Berchemia floribunda* (wall.) Brongn, - *Mallotus apelta* (Lour.) Müll. Arg - *Schefflera arboricola* (Hayata) Merr, - Cinnamomum camphara (L.) J.Presl - *Panax notoginseng* (Burkill) F.H.Chen - [JiangSu Medicine Company (Nangjing, China)] | Y - Authenticated by Prof. Qin min Jian in School of Chinese Herbal Medicine, China Pharmaceutical University and all the voucher specimens were deposited in the Herbarium of School of traditional Chinese medicine, China Pharmaceutical University | Y |
| ([Kou et al., 2015](#_ENREF_59)) | - Indian frankincense (*Boswellia serrata*), 150 g - *Tinospora* spp., 150 g, - *Cassia tora*, 120 g, - *gypsum slag*, 75 g, - *HuangKuizi*, 120 g, - *Acorus calamus (Tibetan subspecies)*, 120 g, - *Justicia adhatoda*, 110 g, - *Acacia catechu*, 75 g, - *Terminalia chebula*, 150 g, - *Styrax benzoin*, 60 g, - *MaoHezi*, 150 g, - *Aconitum pendulum Busch*, 75 g, - *Saussurea lappa*, 150 g, - *musk* (*Moschus* spp.), 1.5 g, - *Phyllanthus emblica*, 150 g - [Qinghai Provincial Tibetan Medical Hospital] | N | N |
| ([Liu et al., 2015a](#_ENREF_77)) | - *Rhizoma Smilacis Glabrae*, 35 g - *Rhizoma Dioscoreae Hypoglaucae*, 18 g, - *Pseudobulbus Cremastrae seu Pleiones*, 15 g - *Semen Vaccariae,* 10 g - *Radix Achyranthis Bidentatae*, 10 g - [Pharmacy of Guangzhou General Hospital] - (batch No. 110916; 10 g/package) | N | N |
| ([Liu et al., 2015b](#_ENREF_78)) | - Isolated soy protein (Solpro 931) and isoflavones (Solgen 40/s) [ Solbar industries Ltd. (Israel)], 25g - Soy flour [LandReclamation Dragon and King Foods Co., Ltd. (Heilongjiang, China)], purified daidzein [Shanxi Sciphar Hi-tech Industry Co. Ltd], 40g | N | Y - HPLC |
| ([Ma et al., 2015a](#_ENREF_86)) | - Cortex of *Phellodendron chinense Schneid* (Rutaceae), 120g - Rhizome of *Atractylodes lancea* (Thunb.) DC. (Asteraceae), 60g - Root of A*chyranthes bidentata* BL (Amaranthaceae), 60g - Seed of *Coix lacryma-jobi* L. (Poaceae), 120g - [Medicinal Materials Co. of Jiangsu Province (Nanjing, P.R. China)] | Y - Prepared according to the State Pharmacopoeia of People's Republic of China (Chinese Pharmacopoeia Committee, 2010) | Y - UPLC–MS |
| ([Ma et al., 2015b](#_ENREF_87)) | - Cortex of *Phellodendron chinense* Schneid (Rutaceae), 120g - Rhizome of Atractylodes lancea (Thunb.) DC.(Asteraceae), 60g - Root of Achyranthes bidentata BL (Amaranthaceae), 60g - Seed of Coix lacryma-jobi L. (Poaceae), 120g - [Medicinal Materials Co.of Jiangsu province (Nanjing, P. R.China] | Y - Prepared according to Pharmacopoeia of People's Republic of China (2010 edition) | Y - UPLC-MS |
| ([Shan et al., 2015](#_ENREF_104)) | - *Dioscoreae Nipponicae* Rhizoma | Y - Prepared according to Chinese Pharmacopeia (2010 Edition) | N |
| ([Sun et al., 2015](#_ENREF_115)) | - *Smilax glabra Roxb.,* rhizome, 35g - *Heterosmilax japonica Kunth*, rhizome, 18g - *Cremastra appendiculata* (D.Don) Makino, Rhizome, 15g - *Vaccaria hispanica* (Mill.) Rauschert, seed, 10g - *Achyranthes bidentata* Blume, root, 10g - [Nanhai Pengyang Pharmaceutical Co., Ltd.] | Y - Identified by Professor Xin-Rong Wu (Pharmacy Department of General Hospital of Guangzhou Military Command, Guangzhou, China) and Director Hui-Chan Hou (Chinese Medicine Department of Guangzhou Institute for Drug Control, Guangzhou, China). Voucher specimens were deposited in the Herbarium of Guangzhou Institute for Drug Control. The dosages of the herbs in XZCBF are based on the theory of Chinese Materia Medica. | Y - HPLC |
| ([Tung et al., 2015](#_ENREF_118)) | - Leaves of *Rhododendron oldhamii* Maxim., [Lion Head Mountain of Taipei county in Taiwan] | Y - The voucher specimen (voucher no. 6) was deposited at the herbarium of the Department of Forestry and Natural Resources, National Chiayi University (NCYU), Taiwan. The species were identified by Dr. Lei-Chen Lin (NCYU). | Y - HPLC |
| ([Wu et al., 2015](#_ENREF_144)) | - The roots and rhizomes of *Smilax riparia*, [Tieling, Liaoning Province, China], 5000g | Y - Authenticated as Smilax riparia by Prof. Ye Zhou from Tianjin Medical University, Tianjin, China. A voucher specimen (SR-2010-10) was stored at the College of Pharmacy, Tianjin Medical University, Tianjin, China. | Y - HPLC |
| ([Yang et al., 2015](#_ENREF_161)) | - *Polyporus umbellatus* (Pers.) Fries, 72g - *Alisma orientale* (Sam.) Juz, 120g - *Atractylodes macrocephala* Koidz.,72g - *Poria cocos* (Schw.) Wolf, 72g - *Cinnamomum cassia* (L.) J. Pres, 48g - [Jiangsu Pharmaceutical Corporation (Nanjing, P. R. China)] | Y - Prepared according to Pharmacopoeia of People's Republic of China (2010 edition) | Y - UPLC–MS |
| ([Zeng et al., 2015](#_ENREF_175)) | - *Lagotis brevituba Maxim*.extract [Chengdu Lotus Pond medicinal material market] | Y - Identified by Researcher Zhong Guoyue | N |
| ([Zhou et al., 2015](#_ENREF_189)) | - Rhizoma dioscoreae nipponese, [Heilongjiang Province Drug Company (Harbin, China)] | Y - The voucher specimens (hlj‑201104) of the herb were authenticated by Professor Ke Fu, Institute of Traditional Chinese Medicine. | N |
| ([Chen et al., 2014a](#_ENREF_4)) | - *Davallia formosana*, [Kaohsiung, Taiwan, in July 2010], 50000g | Y - Authenticated by Dr. Hsien-Chang Chang (Division of Pharmacognosy, National Laboratories of Food and Drugs, Department of Health, Taiwan). A voucher specimen was deposited at the Department of Medicinal Chemistry, College of Pharmacy, Taipei Medical University, Taipei, Taiwan. | Y |
| ([Chen et al., 2014c](#_ENREF_17)) | - Artemisia apiacea, 15g - Radix Gentianae Macrophyllae, 15g - Fructus corni, 10g - rhizoma smilacis glabrae, 15g - Rhizoma Dioscoreae Tokoro, 10g - Ash Bark, 10g - semen plantaginis, 15g - Sappan Wood, 8g - [Dongfang Hospital, the second clinical medical college of Beijing University of Chinese Medicine] | N | N |
| ([Hong et al., 2014](#_ENREF_37)) | - The dried rhizome of *Smilacis Glabrae Rhizoma*, [Beijing Tong Ren Tang Pharmacy (Beijing, China)] | N | Y - HPLC |
| ([Liu et al., 2014](#_ENREF_75)) | - *Poecilobdella manillensis* (Nanning Deying Biology Co. LTD) | N | N |
| ([Lu et al., 2014](#_ENREF_82)) | - Rhizoma Dioscoreae Nipponicae, [the Heilongjiang Province Drug Company (Harbin, China).] | Y - The voucher specimens (hlj-201104) of the herb were authenticated by Professor Ke Fu, Institute of Traditional Chinese Medicine, Heilongjiang University of Chinese Medicine. | Y - HPLC |
| ([Pan et al., 2014](#_ENREF_96)) | - *Cortex Phellodendri Chinensis* - *Rhizome Atractylodis* - *Radix Achyranthis Bidentatae* - *Semen Coicis, Smilacis Glabrae Rhixoma* - [Nanjing Baixin Pharmacy] | Y - Identified by Professor Wu Dekang, Department of Chinese Medicine Identification, School of Pharmacy, Nanjing University of Chinese Medicine | N |
| ([Shang et al., 2014](#_ENREF_105)) | - Artemisia apiacea, 15g - Radix Gentianae Macrophyllae, 15g - Fructus corni, 10g - rhizoma smilacis glabrae, 15g - Rhizoma Dioscoreae Tokoro, 10g - Ash Bark, 10g - semen plantaginis, 15g - Sappan Wood, 8g - [Dongfang Hospital, the second clinical medical college of Beijing University of Chinese Medicine] | N | N |
| ([Su et al., 2014](#_ENREF_113)) | - *Rhizoma Dioscoreae septemlobae*, [commercially available], 400g | Y - Identified by Prof. Zhigang Ma, College of Pharmaceutical Science, Lanzhou University. | Y - HPLC |
| ([Wang et al., 2014](#_ENREF_133)) | - Caulis Lonicerae - Rhizoma Polygoni Cuspidati - Discorea nipponica Makino - [Affiliated Hospital of Medical College Qingdao University] | N | Y |
| ([Wu et al., 2014c](#_ENREF_146)) | - *Smilax riparia* | N | N |
| ([Yan et al., 2014](#_ENREF_156)) | - Leonurus | Y | N |
| ([Chen et al., 2013a](#_ENREF_9)) | - *Rhizoma smilacis glabrae*, - *Coix seed* - *Cichorium intybus* - *Radix Puerariae* - *Plantain herb* - *Rhizoma alismatis* - [Po Chi Lam pharmacy] | Y | Y |
| ([Chen et al., 2013b](#_ENREF_10)) | - The raw materials of the fresh rhizome of *Dolichos falcata* Klein, [July 2011 in Yunan province of China], 1000g | Y - Identified by Prof. Dingrong Wan, College of Pharmaceutical Sciences, South-Central University for Nationalities, China. The voucher specimen (No.: S20120725) has been deposited at the Herbarium situated in College of Pharmaceutical Sciences | Y - HPLC |
| ([Ding et al., 2013](#_ENREF_26)) | - Polyporus, 180 g - Poria, 180 g - Rhizoma alismatis, 300g - Rhizoma atractylodis macrocephalae, 180g - Cortex cinnamomic, 180 g - [Medicinal Materials Co. of Jiangsu Province, China] | Y - Prepared according to the recipe of Wuling San recommended by the State Pharmacopoeia of China (Chinese Pharmacopoeia Committee, 2010) | N |
| ([Hu et al., 2013b](#_ENREF_42)) | - Dried ripe fruit of *Gardenia jasminoides* (Ellis.), [HuangQingRenJian drugstore (No: 1010004)], 100g | Y - Prepared according to State Pharmacopoeia of People’s Republic of China (2010 edition) | N |
| ([Kodithuwakku et al., 2013](#_ENREF_56)) | - *Phragmites communis* Trin., - *Berchemia floribunda (wall)* Brongn, - *Mallotus apelta* (Lour.) Muell.-Arg, - *Schefflera arboricola* Hayata., - *Cinnamomum camphara* (L.) Presl - *Panax notoginseng* - JiangSu Medicine Company (Nangjing, China) | Y - Authenticated by Prof. Qin min Jian in School of Chinese Herbal Medicine, China Pharmaceutical University and all the voucher specimens were deposited in the Herbarium of School of Traditional Chinese Medicine, China Pharmaceutical University. | Y - HPLC |
| ([Liu et al., 2013](#_ENREF_73)) | - *Artemisiae Scopariae Herba* - *Glechomae Herba* - *Rhizome of Whiteback Greenbrier* - Ratio = 1:1:1 | N | N |
| ([Pan et al., 2013](#_ENREF_99)) | - *Radix Achyranthis Sylvestris* - *Pseudobulbus Cremastrae* - *Rhizoma Smilacis Chinae* - *Semen Pharbitidis* - *Pollen Typhae* - [the Shanghai Pudong New Area, Kang Qiao Herbal Pieces Factory (Shanghai, China).] - Ratio = 1.5:1.5:1:1:1.5 | Y - Authentication of these herbs was performed by Professor Xiling Li of the Botany Department, Shanghai University of TCM. Voucher specimens (Collection No. 2009-UA027, 2009-UA028, 2009-UA007, 2009-UA24, and 2009-UA382) were deposited in the Laboratory of Chinese Herbal Medicine and Herbal Formula, Department of TCM, School of Pharmacy, Shanghai University of TCM. | N |
| ([Shi et al., 2013](#_ENREF_107)) | - Cortexes of *Phellodendron chinensis* Schneid, - Rhizome of *Atractylodes chinensis* (Thunb.) DC - Roots of *Achyranthes bidentata* BL., - Seeds of Coix Lachiryma-jobi L. var. maxima Makino, - Stems of *L. japonica* Thunb, - Roots of *S. g1abra* Roxb - [the Jiangsu Chinese Crude Drug Co.] | Y - Identified and authenticated by Professor Chengen Wang, College of Pharmacy, Nanjing University of Traditional Chinese Medicine, Nanjing, China | N |
| ([Silva et al., 2013](#_ENREF_111)) | - The underground parts of *Jatropha isabellei* Mull Arg, [Cacequi (Rio Grande do Sul, Brazil) in May of 2008] | Y - A exsiccate was archived as a voucher specimen in the herbarium of the Biology Department at UFSM (SMDB 11816). | N |
| ([Wei et al., 2013](#_ENREF_137)) | - *Polyrhachis vicina Roger* (Guangxi Nanning Sanle Nourishing health products Development Center) | Y - Identified by Professor He Kaijia of Guangxi Research Institute of Traditional Chinese Medicine | Y - GC-MS |
| ([Xu et al., 2013](#_ENREF_155)) | - *Rhizoma Smilacis Glabrae*, [Hunan Province, China in December 2011 (No. 111207)], 2000g | Y - Identified and authenticated by Prof. Wang-Chun Gen of the School of Pharmacy of the Nanjing University of Chinese Medicine. Voucher specimens were deposited in the Nanjing University of Chinese Medicine, Nanjing 210038, PR China. | Y - HPLC |
| ([Yang et al., 2013](#_ENREF_158)) | - *Caulis Akebiae* - *Achyranthis Bidentatae Radix* - *Plantaginis Semen* - *Leonuri Herba* - *Atractylodes Lancea (Thunb.)Dc.* - *Phellodendri Chinrnsis Cortex* - [Huzhou Hospital of Traditional Chinese Medicine] | Y - Prepared according to Pharmacopoeia of People's Republic of China (2010 edition) | N |
| ([Ho et al., 2012](#_ENREF_35)) | - *Balanophora laxiflora*, [Taichung County, in the midlands of Taiwan], 5000g | Y - Identified by Dr. Chao-Lin Kuo, and a voucher specimen (voucher no. 4672) was deposited in the herbarium of the China Medical University, Taichung, Taiwan. | Y |
| ([Hou et al., 2012](#_ENREF_38)) | - Longan seed, [Joben Bio-Medical Co. (Kaohsiung, Taiwan)] | N | Y - HPLC |
| ([Hua et al., 2012](#_ENREF_43)) | - *Phellodendri Chinensis* Cortex, 10 g - Coicis Semen, 15 g - *Achyranthis Bidentatae* Radix, 15 g - *Dioscoreae Hypoglaucae* Rhizoma, 10 g - *Anemarrhenae* Rhizoma, 5 g - [Zhejiang Chinese crude drug Co.ltd.] | Y -Iidentified by Associate Professor Xilin Chen, College of Pharmaceutical Science, Zhejiang Chinese Medical University, China. | Y - HPLC |
| ([Jiang et al., 2012](#_ENREF_52)) | - The leaves of *Mangifera indica L.* (Anacardiaceae), [Nanning (Guangxi Province, China) in the month of April 2011], 500g | Y - Identified by Professor Sui-Qing Cheng, Henan University of Traditional Chinese Medicine. Voucher specimen (no. 11015) is kept in the herbarium of Hospital. | N |
| ([Kuo et al., 2012](#_ENREF_60)) | - Hibiscus sabdariffa L., [Taitung District Farm - Association, Taiwan.], 100g | N | Y - HPLC |
| ([Shi et al., 2012](#_ENREF_110)) | - The best quality commercial branches of *Morus alba L.* (Moraceae), [Medicinal Materials Co. of Jiangsu Province, China], 500g | Y - The voucher specimen (NU-20010) of the herb was deposited in the Herbarium of Nanjing University and was authenticated by Prof. J.W. Cheng, Nanjing University of Traditional Chinese Medicine, Nanjing, China | Y - HPLC |
| ([Yao et al., 2012](#_ENREF_163)) | - *Rhizoma Dioscoreae Nipponicae*, [Heilongjiang Province Drug Company (Harbin, China)] | N | Y - HPLC |
| ([Yi et al., 2012](#_ENREF_165)) | - *Prunus mume* fruit, [Fujian Pharmaceutical CO., LTD, R.P.China], 500g | Y - Authenticated by Cheng-Fu Li, Xiamen Hospital of Traditional Chinese Medicine, P.R.China (voucher specimen number HU/CE-03172). | N |
| ([Cao et al., 2011](#_ENREF_3)) | - [Shanghai Putuo District Hospital of Traditional Chinese Medicine Preparation Room] | N | N |
| ([Zhang et al., 2011](#_ENREF_181)) | - *Smilacis Glabrae Rhixoma*, [Guangdong Yifang Pharmaceutical Co., Ltd], 35g - *Dioscorea tokoro Makino*, [Guangdong Yifang Pharmaceutical Co., Ltd],18g - *Pseudobulbus Cremastrae Seu Pleiones*, [Guangdong Yifang Pharmaceutical Co., Ltd], 15g - *Vaccariae Semen*, [Guangdong Yifang Pharmaceutical Co., Ltd], 10g - *Achyranthis Bidentatae* Radix, [Guangdong Yifang Pharmaceutical Co., Ltd], 10g | N | N |
| ([Dalbeth et al., 2010](#_ENREF_24)) | - Early season skim milk, late season skim milk and MPC 85 skim milk [Fonterra Co-operative Group, Palmerston North, New Zealand] | N | N |
| ([Hu et al., 2010](#_ENREF_40)) | - *Phellodendron* cortex, 120g - *Atractylodis* rhizome, 60g - *Achyranthes* root, 120g - *Coix* seed, 120g - [Medicinal Materials Co. of Jiangsu Province, PR China.] | Y - Prepared by the Chinese Pharmacopoeia (Chinese Pharmacopoeia  Committee, 2005) | N |
| ([Li et al., 2010](#_ENREF_66)) | - *Angelicae Sinensis Radix* - *Hedysarum Multijugum Maxim.* - [Hebei Baoding Gucheng Medicine Co. LTD] - Ratio = 5:1 | N | N |
| ([Lo et al., 2010](#_ENREF_79)) | - Casein or soya protein [ICN no. 905456; ICN Biomedicals, Inc.] combined with palm oil [(Fluka 70 905; Sigma-Aldrich Co., St Louis, MO, USA] or safflower-seed oil[(ICN Biomedicals, Inc.] | N | N |
| ([Lü et al., 2010](#_ENREF_84)) | - *Cortex Phel lodendri* *Chinensis*, [Anhui Xiehecheng co. ltd.], 125g - *Rhizoma Atracty lodis*, [Anhui Xiehecheng co. ltd.], 125g | Y - Identified by Nanjing University of Chinese Medicine, Pharmacy Professor Chen Jianwei | N |
| ([Wang et al., 2010b](#_ENREF_127)) | - cortex of Phellodendron chinense Schneid (Rutaceae), 120g - rhizome of Atractylodes lancea (Thunb.) DC (Asteraceae), 180g - root of Achyranthes bidentata BL (Amaranthaceae), 60g - [Medicinal Materials Co. of Jiangsu Province, P.R. China.] | Y - Prepared by the Chinese Pharmacopoeia (Chinese Pharmacopoeia Committee, 2005) | Y - HPLC |
| ([Chen et al., 2009](#_ENREF_12)) | - *Crude rhubarb*, 30g - *Calcined oyster shell*, 50g - *Waterplantain* rhizome, 30g - *Red sage* root, 15g - Sophora flower, 30g - Aconite root, 15g - *Scullcap* root, 30g | N | N |
| ([Ma et al., 2009](#_ENREF_91)) | - The aerial parts of *Paederia Scandens* (LOUR.) MERRILL (Rubiaceae), [market specializing in herbs (BoZhou, Herb Market, China) in December of 2006], 3000g | Y - Authenticated by Dr. Wenming Cheng of the Department of Pharmacognosy, School of Pharmacy, Anhui Medical University, Hefei, China, and voucher specimens (EPS) were deposited in our laboratory (Dept. of Pharmacology, Anhui Medical University, Hefei 230032, China). | Y |
| ([Xiang et al., 2009](#_ENREF_151)) | - *Atractylodes Lancea* (Thunb.) Dc, 15g - *Phellodendri Chinrnsis* Cortex, 15g - *Achyranthis Bidentatae* Radix, 15g - *Leonuri Herba*, 15g - *Pseudobulbus Cremastrae Seu Pleiones*, 10g | N | N |
| ([WG et al.](#_ENREF_139)) | - *Polygonum cuspidatum* decoction, [Department of Pharmacy, Dongfang Hospital, Beijing University of Chinese Medicine], (40 g/25 mL) per bag | N | N |

# Supplementary Tables S5. Detailed information on patented formulations, botanical or chemical in the included studies.

| Study | Formulation | Source | Species, concentration | Quality control reported? (Y/N) | Chemical analysis reported? (Y/N) |
| --- | --- | --- | --- | --- | --- |
| ([Fan et al., 2018](#_ENREF_27)) | Tongfengning Capsule | Changchun University of Chinese Medicine Rheumatism Hospital | - N/A | N | N |
| ([Huijuan et al., 2017](#_ENREF_48)) | . Qi-Zhu-Xie-Zhuo-Fang (QZXZF) | SICHUAN  NEO-GREEN pharmaceutical technology CO., LTD | - Astragalus *mongholicus* Bunge, (Astragalus) - *Atractylodes macrocephala* Koidz., (rhizome atractylodis macrocephalae) - *Coix lacryma-jobi* L., (Jobstears Seed) - *Pyrrosia lingua* (Thunb.) Farw., (Pyrrosia Leaf) - *Smilax glabra* Roxb., (Smilacis Glabrae Rhizoma) - *Salvia miltiorrhiza* Bge., (Dan-shen Root) - *Cuscuta chinensis* Lam., (Chinese dodder Seed) - *Isaria cicadae* Miq., (Fungus Sclerotia on Cicada) | N | N |
| ([Xie et al., 2017](#_ENREF_152)) | Compound tufuling oral-liquid | Medical Pieces Co., Ltd., of Zhejiang Chinese Medical University (Hangzhou, Zhejiang, China). | - Rhizoma smilacis Glabrae, 30g - Rhizoma dioscoreae collettii, 30g - *Curcuma longa*, 12g - *Herba siegesbeckiae*, 18g - Rhizoma corydalis, 18g - Semen coicis, 30g - *Loranthus parasiticus*, 15g - Stigma maydis, 15g | N | Y - Rapid-performance liquid chromatography identification method |
| ([Rozza et al., 2016](#_ENREF_103)) | ZinutriK | Akademy Pharma | - Kaempferol (dry extract of Ginkgo Biloba leaf), 10mg - Baicalin (dry extract of Scutellaria Baicalensis root), 50mg - Dry extract of green coffee seed, 100mg - Rutin, 50mg | Y - Each of the components has a purity consistent with the  European Pharmacopoeia requirements. | Y |
| ([Honda et al., 2014](#_ENREF_36)) | Chrysanthemum  flower oil (CFO; Kaneka Chrysflavone^TM^) | Prepared by ([Honda et al., 2014](#_ENREF_36)) | - Ethanolic extract of chrysanthemum flowers, 20% (w/w) - Medium-chain triglyceride (MCT, Riken Vitamin Co., Ltd., Tokyo, Japan) 60% (w/w) - Diglycerine monooleate (Riken Vitamin Co., Ltd.) at about 20% (w/w) | Y - Prepared conformed to the Japanese Pharmacopoeia, 15^th^ edition. | Y |
| ([Zhang et al., 2009](#_ENREF_177); [Zhou et al., 2013](#_ENREF_187)) | A series of tongfeng granule | Yueyang Hospital of Integrative Traditional Chinese and Western Medicine, Shanghai University of Traditional Chinese Medicine | - Huzhang Tongfeng Granule: - Polygoni Cuspidati Rhizoma et Radix, 7.55g - Notopterygii Rhizoma et Radix, 7.55g - Angelicae Sinensis Radix, 7.55g - Artemisiae Scopariae Herba, 7.55g - Atractylodes lancea (Thunb.) DC., 7.55g - Poria Cocos(Schw.) Wolf., 7.55g - Cyathulae Radix, 7.55g - Polyporus Umbellatus(Pers)Fr., 7.55g - Alisma Orientale (Sam.) Juz., 7.55g - Pinus tabulaeformis Carr., 7.55g - Licorice, 7.55g - Yinlian Tongfeng Granule： - Artemisiae Scopariae Herba, 5.58g - Lysimachiae Herba, 5.58g - Maydis Seigma, 5.58g | N | N |
|  |  |  |  |  |  |

# Supplementary Tables S6. Detailed information on isolated chemical compounds in the included studies.

| Study | Compound, concentration | Source | Purity (%) (and grade, if applicable) | Quality control reported? (Y/N) |
| --- | --- | --- | --- | --- |
| ([Adachi et al., 2017](#_ENREF_1)) | Pure compound | Wako Pure Chemical Industries, Ltd. | N/A | N |
| ([Chen et al., 2020a](#_ENREF_11)) | Pure compound | JingZhu Biological Technology (Nanjing, China) | >99% | N |
| ([Cui et al.](#_ENREF_22); [2020](#_ENREF_23)) | Pure compound | Phloretin [MedChemExpress, Monmouth Junction, NJ, USA] | 99.7% | N |
| ([Han et al., 2020](#_ENREF_32)) | Pure compound | Shanghai Macklin Biochemical Co., Ltd. (Shanghai, China) | 95% | N |
| ([Kang et al., 2020](#_ENREF_53)) | Isolated compound | Purified from herbaceous peony flower, [Chemistry Laboratory of Henan University of Chinese Medicine] | 54.72% | N |
| ([Dang et al., 2019](#_ENREF_25)) | Isolated compound | Purified from *Mori Cortex*, [Chenguang Biotech Group Co.,Ltd., Handan, Hebei] | N/A | N |
| ([Lyu et al., 2019](#_ENREF_85)) | Pure compound | Purified form *Pulsatilla Chinensis* by research group (Jiangxi University of Traditional Chinese Medicine, Nanchang, China) | ≥ 98.50% | Y - NMR |
| ([Pan et al., 2019](#_ENREF_97)) | Pure compound | Sigma-Aldrich (St. Louis, MO). | 98% | N |
| ([Wang et al., 2019d](#_ENREF_136)) | Pure compound | National Institutes for Food and Drug Control | ≥ 98% | N |
| ([Xu et al., 2019](#_ENREF_154)) | Pure compound | N/A | N/A | N |
| ([Yang et al., 2019b](#_ENREF_159)) | Isolated compound | Purified from Oxytropis falcata Bunge, [Qinghai, China] | 80% | N |
| ([Yang et al., 2019d](#_ENREF_162)) | Isolated compound | Purified from *Ganoderma atrum* | > 99.8% | N |
| ([Lin et al., 2018](#_ENREF_70)) | Pure compound | Purified form *Gnaphalium affine* D. Don [collected from Yuhuan, Zhejiang province, PR China in September 2015] | > 98% | Y - ^1^H NMR |
| ([Liu et al., 2018](#_ENREF_76)) | Isolated compound | Purified from *Poecilobdella manillensis* Lesson (Nanning Jinhaikekang Biomedical Technology Co. LTD) | 96% | N |
| ([Louxin et al., 2018](#_ENREF_81)) | Pure compound | Chengdu Manster Biotechnology Co., Ltd. | N/A | N |
| ([Qin et al., 2018](#_ENREF_102)) | Pure compound | Department of New Drug Research & Development, Institute of Materia Medica (Beijing, China) | > 95% | Y - ESI-MS and ^1^H NMR |
| ([Wang et al., 2018](#_ENREF_123)) | Pure compound | Aladdin Industrial Corporation (Shanghai, China). | 98% purity, analytical-grade reagent | Y - HPLC |
| ([Zhang et al., 2018b](#_ENREF_183)) | Isolated compound | Obtained from 70% EtOH extract of D. spongiosa [Anguo medicinal market, Hebei province, China] | N/A | Y - HPLC |
| ([Zhu et al., 2018](#_ENREF_192)) | Isolated compound | Purified form Green tea leaves [Chinese market] | 98.34% | Y - HPLC |
| ([Chen et al., 2017b](#_ENREF_18)) | Isolated compound | Extracted and purified from the branches and twigs of *Dipterocarpus Alatus* [the campus of Kasetsart University in Thailand, in April 2010] | 99% | Y – HPLC and LC-MS |
| ([Jiang et al., 2017](#_ENREF_51)) | Isolated compound | Purified form *Gnaphalium* *pensylvanicum* [Yuhuan by Xi-Biao Zhang, Zhejiang province, PR China in October 2015] | N/A | Y - UPLC-ESI-MS/MS |
| ([Li et al., 2017c](#_ENREF_67)) | Isolated compound | Purified from Humulus lupulus | N/A | N |
| ([Liu et al., 2017](#_ENREF_72)) | Pure compound | Zelang Pharmaceutical Co. Ltd. (Nanjing, China) | 95% | N |
| ([Pang et al., 2017](#_ENREF_100)) | Isolated compound | Extracted from *Gynostemma pentaphyllum* [Zelang Medical Technology Co. (Nanjing, Jiangsu, China).] | N/A | N |
| ([Zhou et al., 2017](#_ENREF_188)) | Pure compound | Heilongjiang Province Drug Company | 55.9% | Y - UPLC/MS |
| ([Zhu et al., 2017](#_ENREF_194)) | Isolated compound | Purified form *Dioscorea Collettii* Rhizomes [the First Affiliated Hospital of Anhui University of Chinese Medicine] | 97.5% | Y - HPLC |
| ([Chen et al., 2016b](#_ENREF_16)) | Isolated compound | Purified from dried roots of *Dioscorea septemloba* Thunb [the Guangzhou Chinese Herbal Medicine Co. (China)] | 80.31% | Y – HPLC |
| ([Hui et al., 2016](#_ENREF_47)) | Isolated compound | Purified from the rhizomes of *E. pungens*, [Medicinal Materials Co. of Anhui Province, P.R. China)], 5kg | > 95% | Y - HPLC |
| ([Li et al., 2016](#_ENREF_63)) | Isolated compound | Purified from the roots and rhizomes of S. riparia | 98.8% | Y – HPLC |
| ([Niu et al., 2016](#_ENREF_94)) | Isolated compound | Purified from *Mangifera indica L.*(Anacardiaceae) leaves, [Baise of Guangxi Province, China] | > 90% | Y - HPLC |
| ([Tang et al., 2016](#_ENREF_116)) | Pure compound | The National Institute for the Control of Pharmaceutical and Biological Products (Beijing, China) | > 98% | N |
| ([Chen et al., 2015a](#_ENREF_5)) | Isolated compound | Purified from green tea leaves | 81.41% | Y – HPLC |
| ([Cheng et al., 2015](#_ENREF_19)) | Isolated compound | Purified from Lippia nodiflora [Seremban, Negeri Sembilan, Malaysia, since August 2012] | N/A | Y |
| ([Hou et al., 2015](#_ENREF_39)) | Isolated compound | Purified from *Smilax Riparia* | 98.5% | Y – HPLC |
| ([Lee et al., 2015](#_ENREF_61)) | Pure compound | Provided by Professor Duh Chang-Yi,  Department of Marine Biotechnology and Resources, National Sun Yat-sen University | N/A | N |
| ([Meng et al., 2015](#_ENREF_92)) | Pure compound | Qingze Pharmaceutical, Inc. | 98% | N |
| ([Wang et al., 2015](#_ENREF_122)) | Pure compound | Plant Bioengineering (Xi'an, P. R.China) | > 90.0% | N |
| ([Zhang et al., 2015](#_ENREF_184)) | Isolated compound | Purified from dried purple sweet potato powder [Puzetian Food Co. Limited (Wuhan, China).] | N/A | Y – HPLC-ESI-MS/  MS |
| ([Chen et al., 2014b](#_ENREF_6)) | Isolated compound | Purified from dioscorea | 61.2% | N |
| ([Ma et al., 2014](#_ENREF_88)) | Isolated compound | Purified from *cordyceps militaris* [College of Life Science of South China Normal University, Guangzhou, China.] | Total sugar: 65.2 %,  protein: 0.21 % | N |
| ([Su et al., 2014](#_ENREF_113)) | Isolated compound | Purified from *Rhizoma Dioscoreae septemlobae* | N/A | Y |
| ([Wu et al., 2014a](#_ENREF_143)) | Isolated compound | Purified from the roots and rhizomes of Smilax riparia, [Tieling, Liaoning Province of China] | 97.8% | Y - HPLC |
| ([Wu et al., 2014b](#_ENREF_145)) | Isolated compound | Purified from the roots and rhizomes of S. riparia [Tieling, Liaoning  Province, China] | Compound 1: 96.8%, compound 2:  97.3% | Y – HPLC |
| ([Wu et al., 2014d](#_ENREF_147)) | Isolated compound | Purified from the roots and rhizomes of S. riparia [Tieling, Liaoning Province of China, in September 2011] | N/A | Y - ^1^H NMR |
| ([Zhou et al., 2014](#_ENREF_190)) | Isolated compound | Heilongjiang Province Drug Company. | 55.9% | Y - UPLC fingerprint analysis and  MS |
| ([Hu et al., 2013a](#_ENREF_41)) | Pure compound | Zelang Biotechnology Co., LTD | ≥ 95% | N |
| ([Kondo et al., 2013](#_ENREF_57)) | Isolated compound | Purified from Rooibos (Aspalathus linearis) [ the Rooibos Marketing Ltd. Japan] | 91.5% | Y – HPLC |
| ([Huang et al., 2012](#_ENREF_45)) | Pure compound | Sigma (St. Louis, MO, USA) | > 98% | N |
| ([Niu et al., 2012](#_ENREF_95)) | Isolated compound | Purified from the leaves of *Mangifera indica L. (Anacardiaceae).* | > 90% | Y - HPLC |
| ([Wu et al., 2012a](#_ENREF_141)) | Isolated compound | Purified from *salvia miltiorrhiza*, [Hunyuan County, Shanxi Province of China] | > 98% | Y - HPLC‑UV |
| ([Wu et al., 2012b](#_ENREF_142)) | Isolated compound | Purified from *Salvia miltiorrhiz* [Hunyuan County, Shanxi Province of China.] | > 98% | Y-HPLC |
| ([Zhu et al., 2012](#_ENREF_195)) | Isolated compound | Purified from *Paederia scandens* [BoZhou, Herb Market, China] | N/A | N |
| ([Wang et al., 2011](#_ENREF_120)) | Pure compound | Purified from *Morus alba* L. [Medicinal Materials Co. of Jiangsu Province, R. P. China] | > 97% | Y - HPLC |
| ([Wang et al., 2010a](#_ENREF_119)) | Pure compound | Sigma-Aldrich (St. Louis, MO). | N/A | N |

# Supplementary Tables S7. Composition of herbal formula included in the review.

| **Herbal formula** | **Composition** |
| --- | --- |
| A series of tongfeng granule | *Huzhang Tongfeng Granule: Polygoni Cuspidati Rhizoma Et Radix, Notopterygii Rhizoma Et Radix, Angelicae Sinensis Radix, Artemisiae Scopariae Herba, Atractylodes Lancea (Thunb.)Dc., Poria Cocos(Schw.) Wolf., Cyathulae Radix, Polyporus Umbellatus(Pers)Fr., Alisma Orientale (Sam.) Juz., Pinus tabulaeformis Carr, Licorice; Yinlian Tongfeng Granule: Artemisiae Scopariae Herba, Lysimachiae Herba, Maydis Seigma;*  *Jinhuang Ointment: Curcuma longa Rhizoma, Radix Rhei Et Rhizome, Phellodendri Chinrnsis Cortex, Bletilla striata, A. Dahurica (Fisch.) Benth. Et Hook, Arisaematis Rhizoma, Aucklandiae Radix, Arum Ternatum Thunb., Trichosanthis Radix, Atractylodes Lancea (Thunb.)Dc., Magnolia Officinalis Rehd Et Wils., Citrus Reticulata, Scutellariae Radix, Licorice, Cortex Moutan, Coptidis Rhizoma* |
| *Alismatis Rhizoma and Rhizoma Smilacis Glabrae* decotion | *Alismatis Rhizoma, Rhizoma Smilacis Glabrae* |
| Chuanhutongfeng Mixture | *Rhizome dioscorea nipponica,, Rhizoma polygoni cuspidati, Caulis lonicerae, Radix saposhnikoviae, Radix clematidis, Rhizoma smilacis glabrae, Radix cyathulae, Rhizoma ligustici chuanxiong, Rhizoma dioscoreae hypoglaucae, Fructus chaenomelis, Radix glycyrrhizae, sodium alginate* |
| Compound qingqin liquid | *Artemisia apiacea, Radix Gentianae Macrophyllae, Fructus corni, rhizoma smilacis glabrae, Rhizoma Dioscoreae Tokoro, Ash Bark, semen plantaginis, Sappan Wood* |
| Compound Shuiniujiao | N/A |
| Compound Tufuling Granules | *Rhizoma Smilacis Glabrae, Rhizoma Dioscoreae Hypoglaucae, Pseudobulbus Cremastrae seu Pleiones, Semen Vaccariae, Radix Achyranthis Bidentatae* |
| Compound tufuling oral-liquid | *Rhizoma smilacis Glabrae, Rhizoma dioscoreae collettii, Curcuma longa, Herba siegesbeckiae, Rhizoma corydalis, Semen coicis, Loranthus parasiticus, Stigma maydis* |
| Danggui Buxue Tang | *Angelicae Sinensis Radix, Hedysarum Multijugum Maxim.* |
| *Dendrobium officinalis* Six Nostrum | *Atractylodes, Cork, Coix seed, Achyranthes, Dendrobium officinalis* |
| Er Miao Wan | *Phellodendri Chinensis Cortex, Atractylodis Rhizoma* |
| Erding Granule | *Viola yedoensis Makino, Taraxacum mongolicum Hand-Mazz., Lobelia chinensi Lour, Isatis indigotica Fort.* |
| Huashi Pill | *Desmodium styracifolium, Ventriculi galli mucosa, Alisma orientalis, Sand cattle, Astragalus, Plantago seed, Corydalis corydalis,* Licorice |
| Jianpi Huashi decoction | *Rhizoma Smilacis Glabrae, Plantago asiatica L., kudzu root, Cichorium intybus L., seeds of Coix lacryma-jobi L., Alismatis Rhizoma, tuna extract* |
| Jianpihuashi Decoction | *Rhizoma smilacis glabrae, Coix seed, Cichorium intybus, Radix Puerariae, Plantain herb, Rhizoma alismatis* |
| Jia-Wei-Si-Miao-Wan | N/A |
| Jieduxiezhuo decoction | *Radix Achyranthis Sylvestris, Pseudobulbus Cremastrae, Rhizoma Smilacis Chinae, Semen Pharbitidis, Pollen Typhae* |
| Karapxa decoction | *Apium graveolen L., Apium graveolen L., Cuscuta chinensis Lam., Cichorium glandulosum Boiss. et Huet., Foeniculum vulgare Mill, Cichorium glandulosum Boiss. et Huet.* |
| Modified Sanmiao Powder | *Atractylodes Lancea (Thunb.) Dc., Phellodendri Chinrnsis Cortex, Achyranthis Bidentatae Radix, Leonuri Herba, Pseudobulbus Cremastrae Seu Pleiones* |
| Modified Simiao Decoction | *Phellodendri Chinensis Cortex, Coicis Semen, Achyranthis Bidentatae Radix, Dioscoreae Hypoglaucae Rhizoma, Anemarrhenae Rhizoma* |
| Modified Simiao Wan (2014) | *Cortex Phellodendri Chinensis, Rhizome Atractylodis, Radix Achyranthis Bidentatae, Semen Coicis, Smilacis Glabrae Rhixoma* |
| Modified Simiaowan | *Atractlodis Rhizoma, Phellodendri Chinensis Cortex, Coicis Semen, Achyranthis Bidentatae Radix, Smilax glabra Rhizoma, Lonicera japonica Thunb* |
| Qi-Zhu-Xie-Zhuo-Fang | *Astragalus mongholicus Bunge, Atractylodes macrocephala Koidz., Coix lacryma-jobi L., Pyrrosia lingua (Thunb.) Farw., Smilaxglabra Roxb., Salvia miltiorrhiza Bge., Cuscuta chinensis Lam., Isaria cicadae Miq.* |
| Quzhuotongbi decoction | *Glabrous Greenbrier Rhizome, Dioscorea septemloba Thunb, Maydis stigma 15g, coix seed, Alismatis rhizome, Humulus scandens, Parasiticloranthus, Herba Siegesbeckiae, turmeric, CorydalisRhizoma, Citrus medica* |
| Retention Enema of Chinese Herbal Medicine | *Crude rhubarb, calcined oyster shell, waterplantain rhizome, red sage root, sophora flower, aconite root, scullcap root* |
| RuPeng15 powder | *Indian frankincense (Boswellia serrata), Tinospora spp., Cassia tora, gypsum slag, HuangKuizi, Acorus calamus (Tibetan subspecies), Justicia adhatoda, Acacia catechu, Terminalia chebula, Styrax benzoin, MaoHezi, Aconitum pendulum Busch, Saussurea lappa, musk (Moschus spp.), Phyllanthus emblica* |
| Sanmiao formula | *Atractylodes chinensis (DC.) Koidz., Phellodendron chinense Schneid., Achyranthes bidentata Bl.* |
| Serial Gout Granules | *Huzhang Tongfeng Granule: Polygoni Cuspidati Rhizoma Et Radix, Notopterygii Rhizoma Et Radix, Angelicae Sinensis Radix, Artemisiae Scopariae Herba, Atractylodes Lancea (Thunb.)Dc., Poria Cocos(Schw.) Wolf., Cyathulae Radix, Polyporus Umbellatus(Pers)Fr., Alisma Orientale (Sam.) Juz., Pinus tabulaeformis Carr, Licorice; Yinlian Tongfeng Granule: Artemisiae Scopariae Herba, Lysimachiae Herba, Maydis Seigma.* |
| Shizhifang | *Plantago seeds, white mustard seeds, vaccaria seeds, abutilon seeds.* |
| ShiZhiFang | *Vaccaria tablets, hite mustard seed tablets, abutilon seed tablets, plantago seed tablets* |
| Shuang-Qi gout capsule | *Phragmites communis Trin., Berchemia floribunda (wall.) Brongn., Mallotus apelta (Lour.) Müll. Arg., Schefflera arboricola (Hayata) Merr, Cinnamomum camphora (L.) J.Presl, Panax notoginseng (Burkill) F. H.Chen* |
| Simiao pill | *Cortex Phellodendri Chinensis, Rhizome Atractylodis, Radix Achyranthis Bidentatae, Semen Coicis* |
| Siwu decoction | *Angelica sinensis radix, Chuanxiong rhizome, Paeoniae radix alba, Rehmanniae radix praeparata* |
| Si-Wu-Tang and Er-Miao-San | *Rehmannia glutinosa, Angelica sinensis, Radix Paeoniae Alba, Rhizoma Chuanxiong, Cortex Phellodendri Chinensis, Atractylodes Lancea* |
| Spleen-Kidney Supplementing Formula | *Radix Astragali, Fructus Corni,, Rhizoma Coptidis, Radix Puerariae Lobatae, Cortex Mori, Herba Eupatorii* |
| The Chuanhu anti-gout mixture | *Caulis Lonicerae Rhizoma, Polygoni Cuspidati, Discorea nipponica Makino* |
| The *Selaginella moellendorffii* prescription | *S. moellendorffii Herba, Smilacis glabrae Rhizoma), Plantaginis Semen* |
| Tongfeng Granule | *Artemisiae Scopariae Herba, Glechomae Herba, Rhizome Of Whiteback Greenbrier* |
| Tongfengning Capsule | N/A |
| TongFengTangSan | *Tinospora sinensis, Terminalia chebula Retz, Trogopterori faeces* |
| Tu-Teng-Cao | *Polygonum cuspidatum Sieb.et Zucc., Sargentodoxa cuneate (Oliv.) Rehd. et Wils., Smilax glabra Roxb., Lonicera japonica ;unb., Lysimachia christinae Hance., Phellodendron chinense Schneid., Commiphora myrrha Engl., and Angelica dahurica (Fisch. Ex Hoffm.) Benth. et Hook. f.* |
| Wuling San | *Polyporus, Alismatis rhizome, Atractylodis macrocephalae rhizome, Poria, Cinnamomi cortex* |
| Xiaofeng Granules | *Atractylodes chinensis(DC.) Koidz., Phellodendron chinense Schneid., Coix lacryma-jobi L. var. mayuen(Roman.) Stapf., Achyranthes bidentate Bl., Smilax glabra Roxb., Lonicerajaponica Thunb.* |
| Xiezhuo Chubi Recipe | *Smilacis Glabrae Rhixoma, Dioscorea tokoro Makino, Pseudobulbus Cremastrae Seu Pleiones, Vaccariae Semen, Achyranthis Bidentatae Radix* |
| Xie-Zhuo-Chu-Bi-Fang | *Smilax glabra Roxb., Heterosmilax japonica Kunth, Cremastra appendiculata (D.Don) Makino, Vaccaria hispanica (Mill.) Rauschert, Achyranthes bidentata Blume* |
| Yellow-dragon Wonderful-seed Formula | *Cortex Phellodendri Chinensis, Rhizome Atractylodis, Radix Achyranthis Bidentatae, Semen Coicis, Cardamom, Pheretima* |
| Yellow-dragon Wonderful-seed Formula + Gypsum Fibrosum | *Cortex Phellodendri Chinensis, Rhizome Atractylodis, Radix Achyranthis Bidentatae, Semen Coicis, Cardamom, Pheretima, Gypsum* |
| ZinutriK | *Kaempferol, baicalin, dry extract of green coffee seed and rutin* |
| Zisheng Shenqi decoction | *Radix rehmanniae, praeparata, Cornus officinalis, Poria cocos, Cortex moutan, Rhizoma dioscoreae, Rhizoma alismatis, Amomum villosum, Achyranthes bidentatae, Semen plantaginis, Rhizoma smilacis glabrae, Dioscorea septemloba* |

# Reference

Adachi, S.I., Nihei, K.I., Ishihara, Y., Yoshizawa, F., and Yagasaki, K. (2017). Anti-hyperuricemic effect of taxifolin in cultured hepatocytes and model mice. *Cytotechnology* 69(2)**,** 329-336. doi: 10.1007/s10616-016-0061-4.

Amat, N., Umar, A., Hoxur, P., Anaydulla, M., Imam, G., Aziz, R., et al. (2015). Traditional Uighur Medicine Karapxa decoction, inhibits liver xanthine oxidase and reduces serum uric acid concentrations in hyperuricemic mice and scavenges free radicals in vitro. *BMC Complement Altern Med* 15**,** 131. doi: 10.1186/s12906-015-0644-1.

Cao, W., Xu, D., Wu, W., Xu, T., and Li, T. (2011). Experimental study on anti-inflammation and analgesic effects of extracts of compound Shuiniujiao. *Pharmaceutical Care and Research* 11(2)**,** 103-106. doi: 10.5428/pcar20110209.

Chen, C.Y., Huang, C.C., Tsai, K.C., Huang, W.J., Huang, W.C., Hsu, Y.C., et al. (2014a). Evaluation of the Antihyperuricemic Activity of Phytochemicals from Davallia formosana by Enzyme Assay and Hyperuricemic Mice Model. *Evid Based Complement Alternat Med* 2014**,** 873607. doi: 10.1155/2014/873607.

Chen, G., Tan, M.L., Li, K.K., Leung, P.C., and Ko, C.H. (2015a). Green tea polyphenols decreases uric acid level through xanthine oxidase and renal urate transporters in hyperuricemic mice. *J Ethnopharmacol* 175**,** 14-20. doi: 10.1016/j.jep.2015.08.043.

Chen, G.L., Wu, S., Na, S., and Li, L. (2014b). [Effect of total saponin of dioscorea on uric acid excretion indicators in chronic hyperuricemia rats]. *Zhongguo Zhong Xi Yi Jie He Za Zhi* 34(1)**,** 75-80.

Chen, H.-f., Zhang, C., Yao, Y., Li, J.-m., Du, W.-d., Li, M.-l., et al. (2019). Study on anti-hyperuricemia effects and active ingredients of traditional Tibetan medicine TongFengTangSan (TFTS) by ultra-high-performance liquid chromatography coupled with quadrupole time-of-flight mass spectrometry. *Journal of Pharmaceutical and Biomedical Analysis* 165**,** 213-223. doi: https://doi.org/10.1016/j.jpba.2018.11.038.

Chen, J., Zhou, J., Wei, S., Xie, Z., Wen, C., and Xu, G. (2016a). Effect of a traditional Chinese medicine prescription Quzhuotongbi decoction on hyperuricemia model rats studied by using serum metabolomics based on gas chromatography-mass spectrometry. *J Chromatogr B Analyt Technol Biomed Life Sci* 1026**,** 272-278. doi: 10.1016/j.jchromb.2015.10.031.

Chen, J.W., Zhou, Y., Xue, Z.Y., Li, C., Guo, J., Zhou, L.Y., et al. (2013a). [Effect of jianpihuashi decoction on rats with hyperuricemia]. *Zhong Yao Cai* 36(9)**,** 1486-1489.

Chen, L., Mola, M., Deng, X., Mei, Z., Huang, X., Shu, G., et al. (2013b). Dolichos falcata Klein attenuated the inflammation induced by monosodium urate crystals in vivo and in vitro. *J Ethnopharmacol* 150(2)**,** 545-552. doi: 10.1016/j.jep.2013.08.063.

Chen, M., Ye, C., Zhu, J., Zhang, P., Jiang, Y., Lu, X., et al. (2020a). Bergenin as a Novel Urate-Lowering Therapeutic Strategy for Hyperuricemia. *Frontiers in Cell and Developmental Biology* 8. doi: 10.3389/fcell.2020.00703.

Chen, Q., Ma, L., and Akebaier, W. (2009). Clinical study on treatment of hyperuricaemia by retention enema of Chinese herbal medicine combined with allopurinol. *Chin J Integr Med* 15(6)**,** 431-434. doi: 10.1007/s11655-009-0431-2.

Chen, W.D., Zhao, Y.L., Sun, W.J., He, Y.J., Liu, Y.P., Jin, Q., et al. (2020b). "Kidney Tea" and Its Bioactive Secondary Metabolites for Treatment of Gout. *J Agric Food Chem*. doi: 10.1021/acs.jafc.0c03848.

Chen, W.J., Wu, Y., Bi, R.B., Liu, S., Liu, Z.Y., Liu, Z.Q., et al. (2017a). Therapeutic Effects of Selaginella tamariscina on the Model of Acute Gout with Hyperuricemia in Rats Based on Metabolomics Analysis. *Chinese Journal of Chemistry* 35(7)**,** 1117-1124. doi: 10.1002/cjoc.201600810.

Chen, W.J., Wu, Y., Xu, C., Liu, S., Wang, W.Z., Song, F.R., et al. (2015b). [Study on Therapeutic Effects of Ermiao Pill and Ermiao Pill Categorized Formula in Hyperuricemic Rats Using Spectroscopic Methods]. *Guang Pu Xue Yu Guang Pu Fen Xi* 35(4)**,** 956-960.

Chen, Y., Chen, X.L., Xiang, T., Sun, B.G., Luo, H.X., Liu, M.T., et al. (2016b). Total saponins from dioscorea septemloba thunb reduce serum uric acid levels in rats with hyperuricemia through OATP1A1 up-regulation. *J Huazhong Univ Sci Technolog Med Sci* 36(2)**,** 237-242. doi: 10.1007/s11596-016-1573-z.

Chen, Y., Lu, Y., Wang, Y.N., Lin, Z.C., Gu, W., Tan, L., et al. (2014c). [Effect of compound qingqin liquid on the expression of toll-like receptor in the renal tissue of rats with urate nephropathy]. *Zhongguo Zhong Xi Yi Jie He Za Zhi* 34(6)**,** 722-727.

Chen, Y.S., Chen, C.J., Yan, W., Ge, H.M., and Kong, L.D. (2017b). Anti-hyperuricemic and anti-inflammatory actions of vaticaffinol isolated from Dipterocarpus alatus in hyperuricemic mice. *Chin J Nat Med* 15(5)**,** 330-340. doi: 10.1016/s1875-5364(17)30053-5.

Cheng, L.C., Murugaiyah, V., and Chan, K.L. (2015). Flavonoids and phenylethanoid glycosides from Lippia nodiflora as promising antihyperuricemic agents and elucidation of their mechanism of action. *J Ethnopharmacol* 176**,** 485-493. doi: 10.1016/j.jep.2015.11.025.

Cheng, S., Sun, H., Li, X., Yan, J., Peng, Z., You, Y., et al. (2019). Effects of Alismatis Rhizoma and Rhizoma Smilacis Glabrae Decoction on Hyperuricemia in Rats. *Evidence-based Complementary and Alternative Medicine* 2019. doi: 10.1155/2019/4541609.

Cho, S.S., Song, S.H., Choi, C.Y., Park, K.M., Shim, J.H., and Park, D.H. (2018). Optimization of the Extraction Conditions and Biological Evaluation of Dendropanax morbifera H. Lev as an Anti-Hyperuricemic Source. *Molecules* 23(12). doi: 10.3390/molecules23123313.

Cui, D., Liu, S., Tang, M., Lu, Y., Zhao, M., Mao, R., et al. Phloretin ameliorates hyperuricemia-induced chronic renal dysfunction through inhibiting NLRP3 inflammasome and uric acid reabsorption. (1618-095X (Electronic)).

Cui, D., Liu, S., Tang, M., Lu, Y., Zhao, M., Mao, R., et al. (2020). Phloretin ameliorates hyperuricemia-induced chronic renal dysfunction through inhibiting NLRP3 inflammasome and uric acid reabsorption. *Phytomedicine* 66**,** 153111. doi: 10.1016/j.phymed.2019.153111.

Dalbeth, N., Wong, S., Gamble, G.D., Horne, A., Mason, B., Pool, B., et al. (2010). Acute effect of milk on serum urate concentrations: a randomised controlled crossover trial. *Ann Rheum Dis* 69(9)**,** 1677-1682. doi: 10.1136/ard.2009.124230.

Dang, Y.X., Liang, D.L., Zhou, X.X., Qin, Y., Gao, Y., and Li, W.M. (2019). Protective effect of Mori Cortex on kidney in rats with hyperlipidemia and hyperuricemia based on molecular docking technique. *Chinese Traditional and Herbal Drugs* 50(5)**,** 1175-1181. doi: 10.7501/j.issn.0253-2670.2019.05.022.

Ding, X.Q., Pan, Y., Wang, X., Ma, Y.X., and Kong, L.D. (2013). Wuling san ameliorates urate under-excretion and renal dysfunction in hyperuricemic mice. *Chin J Nat Med* 11(3)**,** 214-221. doi: 10.1016/s1875-5364(13)60019-9.

Fan, H., Zhao, Y., Fu, J., Zhang, H., Wang, D., Gao, Y., et al. (2018). Anti-inflammatory effect of Tongfengning Capsule in rats with acute gouty arthritis and its mechanism. *Journal of Jilin University Medicine Edition* 44(2)**,** 270-274. doi: 10.13481/j.1671-587x.20180212.

Fang, C., Chen, L., He, M., Luo, Y., Zhou, M., Zhang, N., et al. (2019). Molecular mechanistic insight into the anti-hyperuricemic effect of Eucommia ulmoides in mice and rats. *Pharm Biol* 57(1)**,** 112-119. doi: 10.1080/13880209.2019.1568510.

Fei, Y., Ye, D., Fan, X.F., and Dong, F.Q. (2016). Effect of Dioscorea tokoro Makino extract on hyperuricemia in mice. *Tropical Journal of Pharmaceutical Research* 15(9)**,** 1883-1887. doi: 10.4314/tjpr.v15i9.10.

Guo, L.F., Chen, X., Lei, S.S., Li, B., Zhang, N.Y., Ge, H.Z., et al. (2020). Effects and Mechanisms of Dendrobium officinalis Six Nostrum for Treatment of Hyperuricemia with Hyperlipidemia. *Evid Based Complement Alternat Med* 2020**,** 2914019. doi: 10.1155/2020/2914019.

Guo, Y., Jiang, Q., Gui, D., and Wang, N. (2015). Chinese Herbal Formulas Si-Wu-Tang and Er-Miao-San Synergistically Ameliorated Hyperuricemia and Renal Impairment in Rats Induced by Adenine and Potassium Oxonate. *Cell Physiol Biochem* 37(4)**,** 1491-1502. doi: 10.1159/000438517.

Han, B., Gong, M., Li, Z., Qiu, Y., and Zou, Z. (2020). NMR-Based Metabonomic Study Reveals Intervention Effects of Polydatin on Potassium Oxonate-Induced Hyperuricemia in Rats. *Oxid Med Cell Longev* 2020**,** 6943860. doi: 10.1155/2020/6943860.

Han, B., Zhu, C.X., Shi, W., Huang, H.Z., Hu, X.G., Zhou, X.M., et al. (2017). Effect of Rhizoma Polygoni Cuspidati and Ramulus Cinnamomi compatibility on uric acid metabolism and urinary neutrophil gelatinase-associated lipocalin and kidney injury molecule-1 in rats with hyperuricemia. *Chin J Integr Med* 23(7)**,** 535-542. doi: 10.1007/s11655-016-2649-0.

Han, J., Xie, Y., Sui, F., Liu, C., Du, X., Liu, C., et al. (2016). Zisheng Shenqi decoction ameliorates monosodium urate crystal-induced gouty arthritis in rats through anti-inflammatory and anti-oxidative effects. *Mol Med Rep* 14(3)**,** 2589-2597. doi: 10.3892/mmr.2016.5526.

Ho, S.T., Tung, Y.T., Huang, C.C., Kuo, C.L., Lin, C.C., Yang, S.C., et al. (2012). The Hypouricemic Effect of Balanophora laxiflora Extracts and Derived Phytochemicals in Hyperuricemic Mice. *Evid Based Complement Alternat Med* 2012**,** 910152. doi: 10.1155/2012/910152.

Honda, S., Kawamoto, S., Tanaka, H., Kishida, H., Kitagawa, M., Nakai, Y., et al. (2014). Administered chrysanthemum flower oil attenuates hyperuricemia: mechanism of action as revealed by DNA microarray analysis. *Biosci Biotechnol Biochem* 78(4)**,** 655-661. doi: 10.1080/09168451.2014.890028.

Hong, Q., Yu, S., Mei, Y., Lv, Y., Chen, D., Wang, Y., et al. (2014). Smilacis Glabrae Rhizoma reduces oxidative stress caused by hyperuricemia via upregulation of catalase. *Cell Physiol Biochem* 34(5)**,** 1675-1685. doi: 10.1159/000366369.

Hou, C.W., Lee, Y.C., Hung, H.F., Fu, H.W., and Jeng, K.C. (2012). Longan seed extract reduces hyperuricemia via modulating urate transporters and suppressing xanthine oxidase activity. *Am J Chin Med* 40(5)**,** 979-991. doi: 10.1142/s0192415x12500723.

Hou, P.Y., Mi, C., He, Y., Zhang, J., Wang, S.Q., Yu, F., et al. (2015). Pallidifloside D from Smilax riparia enhanced allopurinol effects in hyperuricemia mice. *Fitoterapia* 105**,** 43-48. doi: 10.1016/j.fitote.2015.06.002.

Hu, Q.H., Jiao, R.Q., Wang, X., Lv, Y.Z., and Kong, L.D. (2010). Simiao pill ameliorates urate underexcretion and renal dysfunction in hyperuricemic mice. *J Ethnopharmacol* 128(3)**,** 685-692. doi: 10.1016/j.jep.2010.02.012.

Hu, Q.H., Miao, M.X., Lu, G., and Ji, H. (2013a). Effects of quercetin on expression of renal NLRP3 and TLRs in rats with uric acid nephtopathy. *Chinese Traditional and Herbal Drugs* 44(24)**,** 3496-3502. doi: 10.7501/j.issn.0253-2670.2013.24.013.

Hu, Q.H., Zhu, J.X., Ji, J., Wei, L.L., Miao, M.X., and Ji, H. (2013b). Fructus Gardenia Extract ameliorates oxonate-induced hyperuricemia with renal dysfunction in mice by regulating organic ion transporters and mOIT3. *Molecules* 18(8)**,** 8976-8993. doi: 10.3390/molecules18088976.

Hua, J., Huang, P., Zhu, C.M., Yuan, X., and Yu, C.H. (2012). Anti-hyperuricemic and nephroprotective effects of Modified Simiao Decoction in hyperuricemic mice. *J Ethnopharmacol* 142(1)**,** 248-252. doi: 10.1016/j.jep.2012.04.052.

Huang, B., Hu, X., Wang, J., Li, P., and Chen, J. (2019a). Study on chemical constituents of herbal formula Er Miao Wan and GC-MS based metabolomics approach to evaluate its therapeutic effects on hyperuricemic rats. *J Chromatogr B Analyt Technol Biomed Life Sci* 1118-1119**,** 101-108. doi: 10.1016/j.jchromb.2019.04.032.

Huang, J., Zhu, M., Tao, Y., Wang, S., Chen, J., Sun, W., et al. (2012). Therapeutic properties of quercetin on monosodium urate crystal-induced inflammation in rat. *J Pharm Pharmacol* 64(8)**,** 1119-1127. doi: 10.1111/j.2042-7158.2012.01504.x.

Huang, L., Deng, J., Chen, G., Zhou, M., Liang, J., Yan, B., et al. (2019b). The anti-hyperuricemic effect of four astilbin stereoisomers in Smilax glabra on hyperuricemic mice. *J Ethnopharmacol* 238**,** 111777. doi: 10.1016/j.jep.2019.03.004.

Hui, W., Yongliang, Y., Yongde, C., Guo, L., Li, L., Zhonglin, Y., et al. (2016). Hypouricemic and Nephroprotective Effects of Emodinol in Oxonate-Induced Hyperuricemic Mice are Mediated by Organic Ion Transporters and OIT3. *Planta Med* 82(4)**,** 289-297. doi: 10.1055/s-0035-1558212.

Huijuan, W., Xiaoxu, C., Rui, S., Xinghui, L., Beibei, T., and Jianchun, M. (2017). Qi-Zhu-Xie-Zhuo-Fang reduces serum uric acid levels and ameliorates renal fibrosis in hyperuricemic nephropathy rats. *Biomed Pharmacother* 91**,** 358-365. doi: 10.1016/j.biopha.2017.04.031.

Huo, L.N., Wang, W., Zhang, C.Y., Shi, H.B., Liu, Y., Liu, X.H., et al. (2015). Bioassay-Guided Isolation and Identification of Xanthine Oxidase Inhibitory Constituents from the Leaves of Perilla frutescens. *Molecules* 20(10)**,** 17848-17859. doi: 10.3390/molecules201017848.

Jhang, J.J., Ong, J.W., Lu, C.C., Hsu, C.L., Lin, J.H., Liao, J.W., et al. (2016). Hypouricemic effects of Mesona procumbens Hemsl. through modulating xanthine oxidase activity in vitro and in vivo. *Food Funct* 7(10)**,** 4239-4246. doi: 10.1039/c6fo00822d.

Jiang, Y., Lin, Y., Hu, Y.J., Song, X.J., Pan, H.H., and Zhang, H.J. (2017). Caffeoylquinic acid derivatives rich extract from Gnaphalium pensylvanicum willd. Ameliorates hyperuricemia and acute gouty arthritis in animal model. *BMC Complement Altern Med* 17(1)**,** 320. doi: 10.1186/s12906-017-1834-9.

Jiang, Y., You, X.Y., Fu, K.L., and Yin, W.L. (2012). Effects of Extract from Mangifera indica Leaf on Monosodium Urate Crystal-Induced Gouty Arthritis in Rats. *Evid Based Complement Alternat Med* 2012**,** 967573. doi: 10.1155/2012/967573.

Kang, L., Miao, J.X., Cao, L.H., Miao, Y.Y., Miao, M.S., Liu, H.J., et al. (2020). Total glucosides of herbaceous peony (Paeonia lactiflora Pall.) flower attenuate adenine- and ethambutol-induced hyperuricaemia in rats. *J Ethnopharmacol* 261**,** 113054. doi: 10.1016/j.jep.2020.113054.

Kim, J.K., Kim, W.J., Hyun, J.M., Lee, J.S., Kwon, J.G., Seo, C., et al. (2017). Salvia plebeia Extract Inhibits Xanthine Oxidase Activity In Vitro and Reduces Serum Uric Acid in an Animal Model of Hyperuricemia. *Planta Med* 83(17)**,** 1335-1341. doi: 10.1055/s-0043-111012.

Kodithuwakku, N.D., Feng, Y.D., Zhang, Y.Y., Pan, M., Fang, W.R., and Li, Y.M. (2015). The molecular insight into the antihyperuricemic and renoprotective effect of Shuang Qi gout capsule in mice. *J Ethnopharmacol* 163**,** 278-289. doi: 10.1016/j.jep.2015.01.013.

Kodithuwakku, N.D., Pan, M., Zhu, Y.L., Zhang, Y.Y., Feng, Y.D., Fang, W.R., et al. (2013). Anti-inflammatory and antinociceptive effects of Chinese medicine SQ gout capsules and its modulation of pro-inflammatory cytokines focusing on gout arthritis. *J Ethnopharmacol* 150(3)**,** 1071-1079. doi: 10.1016/j.jep.2013.10.016.

Kondo, M., Hirano, Y., Nishio, M., Furuya, Y., Nakamura, H., and Watanabe, T. (2013). Xanthine oxidase inhibitory activity and hypouricemic effect of aspalathin from unfermented rooibos. *J Food Sci* 78(12)**,** H1935-1939. doi: 10.1111/1750-3841.12304.

Kou, Y., Li, Y., Ma, H., Li, W., Li, R., and Dang, Z. (2016). Uric acid lowering effect of Tibetan Medicine RuPeng15 powder in animal models of hyperuricemia. *J Tradit Chin Med* 36(2)**,** 205-210.

Kou, Y.Y., Li, Y.F., Xu, M., Li, W.Y., Yang, M., and Li, R.L. (2015). Effects of RuPeng15 Powder (RPP15) on Monosodium Urate Crystal-Induced Gouty Arthritis in Rats. *Evid Based Complement Alternat Med* 2015**,** 527019. doi: 10.1155/2015/527019.

Kuo, C.-Y., Kao, E.-S., Chan, K.-C., Lee, H.-J., Huang, T.-F., and Wang, C.-J. (2012). Hibiscus sabdariffa L. extracts reduce serum uric acid levels in oxonate-induced rats. *Journal of Functional Foods* 4(1)**,** 375-381. doi: https://doi.org/10.1016/j.jff.2012.01.007.

Lee, H.P., Lin, Y.Y., Duh, C.Y., Huang, S.Y., Wang, H.M., Wu, S.F., et al. (2015). Lemnalol attenuates mast cell activation and osteoclast activity in a gouty arthritis model. *J Pharm Pharmacol* 67(2)**,** 274-285. doi: 10.1111/jphp.12331.

Lee, Y.M., Shon, E.J., Kim, O.S., and Kim, D.S. (2017). Effects of Mollugo pentaphylla extract on monosodium urate crystal-induced gouty arthritis in mice. *BMC Complement Altern Med* 17(1)**,** 447. doi: 10.1186/s12906-017-1955-1.

Li, H.G., Hou, P.Y., Zhang, X., He, Y., Zhang, J., Wang, S.Q., et al. (2016). Hypouricemic effect of allopurinol are improved by Pallidifloside D based on the uric acid metabolism enzymes PRPS, HGPRT and PRPPAT. *Fitoterapia* 113**,** 1-5. doi: 10.1016/j.fitote.2016.06.015.

Li, L., Teng, M., Liu, Y., Qu, Y., Zhang, Y., Lin, F., et al. (2017a). Anti-Gouty Arthritis and Antihyperuricemia Effects of Sunflower (Helianthus annuus) Head Extract in Gouty and Hyperuricemia Animal Models. *Biomed Res Int* 2017**,** 5852076. doi: 10.1155/2017/5852076.

Li, Y.P., Wu, S., Ran, A., Xu, D.Y., Wei, J.M., and Zhao, Z.L. (2017b). ARISTOLOCHIA BRACTEOLATE RETZ. ATTENUATES HYPERURICEMIA IN A METABOLIC ARTHRITIS RAT MODEL. *Afr J Tradit Complement Altern Med* 14(4)**,** 180-187. doi: 10.21010/ajtcam.v14i4.21.

Li, Y.Q., Wei, Y.H., and Han, X. (2010). Effects of Danggui Buxue Tang on heart muscle function in hyperuricemic rats. *Chinese Pharmaceutical Journal* 45(7)**,** 524-526.

Li, Z.J., Li, Z., Dong, X.Y., Lu, L.F., and Wang, C.L. (2017c). Hypouricemic and nephroprotective effects of total flavonoids from the residue of supercritical CO2 extraction of Humulus lupulus in potassium oxonate-induced mice. *Pak J Pharm Sci* 30(2)**,** 493-497.

Liang, G., Nie, Y., Chang, Y., Zeng, S., Liang, C., Zheng, X., et al. (2019). Protective effects of Rhizoma smilacis glabrae extracts on potassium oxonate- and monosodium urate-induced hyperuricemia and gout in mice. *Phytomedicine* 59**,** 152772. doi: 10.1016/j.phymed.2018.11.032.

Lin, X., Shao, T., Huang, L., Wen, X., Wang, M., Wen, C., et al. (2020). Simiao Decoction Alleviates Gouty Arthritis by Modulating Proinflammatory Cytokines and the Gut Ecosystem. *Front Pharmacol* 11**,** 955. doi: 10.3389/fphar.2020.00955.

Lin, Y., Liu, P.G., Liang, W.Q., Hu, Y.J., Xu, P., Zhou, J., et al. (2018). Luteolin-4'-O-glucoside and its aglycone, two major flavones of Gnaphalium affine D. Don, resist hyperuricemia and acute gouty arthritis activity in animal models. *Phytomedicine* 41**,** 54-61. doi: 10.1016/j.phymed.2018.02.002.

Liu, G., Chen, X., Lu, X., Zhao, J., and Li, X. (2020). Sunflower head enzymatic hydrolysate relives hyperuricemia by inhibiting crucial proteins (xanthine oxidase, adenosine deaminase, uric acid transporter1) and restoring gut microbiota in mice. *Journal of Functional Foods* 72. doi: 10.1016/j.jff.2020.104055.

Liu, H.J., Pan, X.X., Liu, B.Q., Gui, X., Hu, L., Jiang, C.Y., et al. (2017). Grape seed-derived procyanidins alleviate gout pain via NLRP3 inflammasome suppression. *J Neuroinflammation* 14(1)**,** 74. doi: 10.1186/s12974-017-0849-y.

Liu, J., Xu, L.L., and Xu, Y. (2013). Antigout active fractions in Tongfeng Granule. *Chinese Traditional and Herbal Drugs* 44(5)**,** 590-594. doi: 10.7501/j.issn.0253-2670.2013.05.018.

Liu, N., Wang, Y., Yang, M., Bian, W., Zeng, L., Yin, S., et al. (2019). New Rice-Derived Short Peptide Potently Alleviated Hyperuricemia Induced by Potassium Oxonate in Rats. *J Agric Food Chem* 67(1)**,** 220-228. doi: 10.1021/acs.jafc.8b05879.

Liu, X.H., Huang, M.Q., Lin, Z.W., Zhen, H.S., Liu, A.T., and Zhang, K.Y. (2014). Anti-gout effect of Poecilobdella manillensis. *Chinese Traditional and Herbal Drugs* 45(12)**,** 1747-1750. doi: 10.7501/j.issn.0253-2670.2014.12.018.

Liu, X.H., Zhao, Y.X., Zhou, Y.M., Huang, M.Q., Huang, S.S., Zhen, H.S., et al. (2018). Anti-gout effect of hirudin and its mechanism. *Chinese Traditional and Herbal Drugs* 49(6)**,** 1365-1370. doi: 10.7501/j.issn.0253-2670.2018.06.020.

Liu, Y.W., Sun, W.F., Zhang, X.X., Li, J., and Zhang, H.H. (2015a). Compound Tufuling Granules ([characters: see text]) regulate glucose transporter 9 expression in kidney to influence serum uric acid level in hyperuricemia mice. *Chin J Integr Med* 21(11)**,** 823-829. doi: 10.1007/s11655-015-2052-2.

Liu, Z.M., Ho, C.S., Chen, Y.M., and Woo, J. (2015b). Can soy intake affect serum uric acid level? Pooled analysis from two 6-month randomized controlled trials among Chinese postmenopausal women with prediabetes or prehypertension. *Eur J Nutr* 54(1)**,** 51-58. doi: 10.1007/s00394-014-0684-1.

Lo, H.C., Wang, Y.H., Chiou, H.Y., Lai, S.H., and Yang, Y. (2010). Relative efficacy of casein or soya protein combined with palm or safflower-seed oil on hyperuricaemia in rats. *Br J Nutr* 104(1)**,** 67-75. doi: 10.1017/s0007114510000310.

Lou, X.J., Wang, Y.Z., Lei, S.S., He, X., Lu, T.T., Zhan, L.H., et al. (2020). Beneficial Effects of Macroporous Resin Extract of Dendrobium candidum Leaves in Rats with Hyperuricemia Induced by a High-Purine Diet. *Evid Based Complement Alternat Med* 2020**,** 3086106. doi: 10.1155/2020/3086106.

Louxin, B., Miaoyanyan, F., and Miaomingsan (2018). Effect of arctiin on rat hyperuricemia model. *Acta Medica Mediterranea* 34(6)**,** 1739-1742. doi: 10.19193/0393-6384_2018_6_266.

Lu, F., Liu, L., Yu, D.H., Li, X.Z., Zhou, Q., and Liu, S.M. (2014). Therapeutic effect of Rhizoma Dioscoreae Nipponicae on gouty arthritis based on the SDF-1/CXCR 4 and p38 MAPK pathway: an in vivo and in vitro study. *Phytother Res* 28(2)**,** 280-288. doi: 10.1002/ptr.4997.

Lu, X.Y., Zhou, F.H., Dong, Y.Q., Gong, L.N., Li, Q.Y., Tang, L., et al. (2018). Codonopsis tangshen Oliv. Amelioration Effect on Diabetic Kidney Disease Rats Induced by High Fat Diet Feeding Combined with Streptozotocin. *Nat Prod Bioprospect* 8(6)**,** 441-451. doi: 10.1007/s13659-018-0187-5.

Lü, Y.Z., Hu, Q.H., Wang, X., Ouyang, Z., and Kong, L.D. (2010). Effects of Ermiao Pill water extracts on imbalance of urate levels and its related genes and protein levels in hyperuricemic mice. *Chinese Traditional and Herbal Drugs* 41(3)**,** 418-423.

Lyu, S., Ding, R., Liu, P., OuYang, H., Feng, Y., Rao, Y., et al. (2019). LC-MS Analysis of Serum for the Metabolomic Investigation of the Effects of Pulchinenoside b4 Administration in Monosodium Urate Crystal-Induced Gouty Arthritis Rat Model. *Molecules* 24(17). doi: 10.3390/molecules24173161.

Ma, C.H., Kang, L.L., Ren, H.M., Zhang, D.M., and Kong, L.D. (2015a). Simiao pill ameliorates renal glomerular injury via increasing Sirt1 expression and suppressing NF-kappaB/NLRP3 inflammasome activation in high fructose-fed rats. *J Ethnopharmacol* 172**,** 108-117. doi: 10.1016/j.jep.2015.06.015.

Ma, C.H., Kang, L.L., Ren, H.M., Zhang, D.M., and Kong, L.D. (2015b). Simiao pill ameliorates renal glomerular injury via increasing Sirt1 expression and suppressing NF-κB/NLRP3 inflammasome activation in high fructose-fed rats. *J Ethnopharmacol* 172**,** 108-117. doi: 10.1016/j.jep.2015.06.015.

Ma, L., Zhang, S., Yuan, Y., and Gao, J. (2014). Hypouricemic actions of exopolysaccharide produced by Cordyceps militaris in potassium oxonate-induced hyperuricemic mice. *Curr Microbiol* 69(6)**,** 852-857. doi: 10.1007/s00284-014-0666-9.

Ma, T.H., Sheng, T., Tian, C.M., Xing, M.Y., Yan, L.J., and Xia, D.Z. (2019a). [Effect of ethanolic extract of Polygonum cuspidatum on acute gouty arthritis in mice through NLRP3/ASC/caspase-1 axis]. *Zhongguo Zhong Yao Za Zhi* 44(3)**,** 546-552. doi: 10.19540/j.cnki.cjcmm.20180925.001.

Ma, W.G., Wang, J., Bu, X.W., Zhang, H.H., Zhang, J.P., Zhang, X.X., et al. (2019b). Effects of Polygonum cuspidatum on AMPK-FOXO3α Signaling Pathway in Rat Model of Uric Acid-Induced Renal Damage. *Chinese Journal of Integrative Medicine* 25(3)**,** 182-189. doi: 10.1007/s11655-017-2979-6.

Ma, Y., Zhou, L.L., Yan, H.Y., and Liu, M. (2009). Effects of extracts from Paederia scandens (LOUR.) MERRILL (Rubiaceae) on MSU crystal-induced rats gouty arthritis. *Am J Chin Med* 37(4)**,** 669-683. doi: 10.1142/s0192415x09007156.

Meng, Z., Yan, Y., Tang, Z., Guo, C., Li, N., Huang, W., et al. (2015). Anti-hyperuricemic and nephroprotective effects of rhein in hyperuricemic mice. *Planta Med* 81(4)**,** 279-285. doi: 10.1055/s-0034-1396241.

Moher, D., Liberati, A., Tetzlaff, J., and Altman, D.G. (2009). Preferred reporting items for systematic reviews and meta-analyses: the PRISMA statement. *Ann Intern Med* 151(4)**,** 264-269, w264. doi: 10.7326/0003-4819-151-4-200908180-00135.

Niu, Y., Liu, J., Liu, H.Y., Gao, L.H., Feng, G.H., Liu, X., et al. (2016). Hypouricaemic action of mangiferin results from metabolite norathyriol via inhibiting xanthine oxidase activity. *Pharm Biol* 54(9)**,** 1680-1686. doi: 10.3109/13880209.2015.1120322.

Niu, Y., Lu, W., Gao, L., Lin, H., Liu, X., and Li, L. (2012). Reducing effect of mangiferin on serum uric acid levels in mice. *Pharm Biol* 50(9)**,** 1177-1182. doi: 10.3109/13880209.2012.663763.

Pan, H.Y., Shi, L., Xu, L., Yin, L., Zeng, W.P., Zhang, G.J., et al. (2014). Effect of active fractions from modified Simiao Wan on hyperuricemia and its mechanism. *Chinese Journal of Pharmacology and Toxicology* 28(3)**,** 380-385. doi: 10.3867/j.issn.1000-3002.2014.03.012.

Pan, J., Shi, M., Li, L., Liu, J., Guo, F., Feng, Y., et al. (2019). Pterostilbene, a bioactive component of blueberries, alleviates renal fibrosis in a severe mouse model of hyperuricemic nephropathy. *Biomed Pharmacother* 109**,** 1802-1808. doi: 10.1016/j.biopha.2018.11.022.

Pan, J., Zhang, C., Shi, M., Guo, F., Liu, J., Li, L., et al. (2020). Ethanol extract of Liriodendron chinense (Hemsl.) Sarg barks attenuates hyperuricemic nephropathy by inhibiting renal fibrosis and inflammation in mice. *J Ethnopharmacol***,** 113278. doi: 10.1016/j.jep.2020.113278.

Pan, Y., Chu, Z., Wang, W., and Yang, B. (2013). Pretreatment with Jieduxiezhuo decoction impedes elevations in serum uric acid levels in mice. *J Tradit Chin Med* 33(6)**,** 794-797. doi: 10.1016/s0254-6272(14)60014-5.

Pang, M., Fang, Y., Chen, S., Zhu, X., Shan, C., Su, J., et al. (2017). Gypenosides Inhibits Xanthine Oxidoreductase and Ameliorates Urate Excretion in Hyperuricemic Rats Induced by High Cholesterol and High Fat Food (Lipid Emulsion). *Med Sci Monit* 23**,** 1129-1140. doi: 10.12659/msm.903217.

Peng, A., Lin, L., Zhao, M., and Sun, B. (2019). Identifying mechanisms underlying the amelioration effect of Chrysanthemum morifolium Ramat. 'Boju' extract on hyperuricemia using biochemical characterization and UPLC-ESI-QTOF/MS-based metabolomics. *Food Funct* 10(12)**,** 8042-8055. doi: 10.1039/c9fo01821b.

Qin, Z., Wang, S., Lin, Y., Zhao, Y., Yang, S., Song, J., et al. (2018). Antihyperuricemic effect of mangiferin aglycon derivative J99745 by inhibiting xanthine oxidase activity and urate transporter 1 expression in mice. *Acta Pharm Sin B* 8(2)**,** 306-315. doi: 10.1016/j.apsb.2017.05.004.

Rozza, F., Trimarco, V., Izzo, R., Grassi, D., and Ferri, C. (2016). Effects of a Novel Fixed Combination of Nutraceuticals on Serum Uric Acid Concentrations and the Lipid Profile in Asymptomatic Hyperuricemic Patients : Results from the PICONZ-UA Study. *High Blood Press Cardiovasc Prev* 23(4)**,** 381-386. doi: 10.1007/s40292-016-0168-x.

Shan, H.L., Shan, R.P., and Fu, X.C. (2015). [Hypouricemic effect of ethanol extracts from Dioscoreae Nipponicae Rhizoma]. *Zhejiang Da Xue Xue Bao Yi Xue Ban* 44(1)**,** 49-53.

Shang, X.Z., Ma, W.G., Chen, Y., Lu, Y., Wang, Y.N., Xu, Y.M., et al. (2014). [Effect of compound qingqin liquid on the expression levels of ang II and COX-2 mRNA transcription and protein expression in the renal tissue of uric acid nephropathy rats: an experimental study]. *Zhongguo Zhong Xi Yi Jie He Za Zhi* 34(7)**,** 819-825.

Sheu, S.Y., Fu, Y.T., Huang, W.D., Chen, Y.A., Lei, Y.C., Yao, C.H., et al. (2016). Evaluation of Xanthine Oxidase Inhibitory Potential and In vivo Hypouricemic Activity of Dimocarpus longan Lour. Extracts. *Pharmacogn Mag* 12(Suppl 2)**,** S206-212. doi: 10.4103/0973-1296.182176.

Shi, L., Xu, L., Yang, Y., Song, H., Pan, H., and Yin, L. (2013). Suppressive effect of modified Simiaowan on experimental gouty arthritis: an in vivo and in vitro study. *J Ethnopharmacol* 150(3)**,** 1038-1044. doi: 10.1016/j.jep.2013.10.023.

Shi, L., Zhao, F., Zhu, F., Liang, Y., Yang, F., Zhang, G., et al. (2016a). Traditional Chinese Medicine Formula "Xiaofeng granules" suppressed gouty arthritis animal models and inhibited the proteoglycan degradation on chondrocytes induced by monosodium urate. *J Ethnopharmacol* 191**,** 254-263. doi: 10.1016/j.jep.2016.06.008.

Shi, Y.C., Lin, K.S., Jhai, Y.F., Lee, B.H., Han, Y., Cui, Z., et al. (2016b). Miracle Fruit (Synsepalum dulcificum) Exhibits as a Novel Anti-Hyperuricaemia Agent. *Molecules* 21(2)**,** 140. doi: 10.3390/molecules21020140.

Shi, Y.W., Wang, C.P., Wang, X., Zhang, Y.L., Liu, L., Wang, R.W., et al. (2012). Uricosuric and nephroprotective properties of Ramulus Mori ethanol extract in hyperuricemic mice. *J Ethnopharmacol* 143(3)**,** 896-904. doi: 10.1016/j.jep.2012.08.023.

Silva, C.R., Frohlich, J.K., Oliveira, S.M., Cabreira, T.N., Rossato, M.F., Trevisan, G., et al. (2013). The antinociceptive and anti-inflammatory effects of the crude extract of Jatropha isabellei in a rat gout model. *J Ethnopharmacol* 145(1)**,** 205-213. doi: 10.1016/j.jep.2012.10.054.

Song, S.H., Park, D.H., Bae, M.S., Choi, C.Y., Shim, J.H., Yoon, G., et al. (2018). Ethanol Extract of Cudrania tricuspidata Leaf Ameliorates Hyperuricemia in Mice via Inhibition of Hepatic and Serum Xanthine Oxidase Activity. *Evid Based Complement Alternat Med* 2018**,** 8037925. doi: 10.1155/2018/8037925.

Su, J., Wei, Y., Liu, M., Liu, T., Li, J., Ji, Y., et al. (2014). Anti-hyperuricemic and nephroprotective effects of Rhizoma Dioscoreae septemlobae extracts and its main component dioscin via regulation of mOAT1, mURAT1 and mOCT2 in hypertensive mice. *Arch Pharm Res* 37(10)**,** 1336-1344. doi: 10.1007/s12272-014-0413-6.

Su, Q., Su, H., Nong, Z., Li, D., Wang, L., Chu, S., et al. (2018). Hypouricemic and Nephroprotective Effects of an Active Fraction from Polyrhachis Vicina Roger On Potassium Oxonate-Induced Hyperuricemia in Rats. *Kidney Blood Press Res* 43(1)**,** 220-233. doi: 10.1159/000487675.

Sun, W.F., Zhu, M.M., Li, J., Zhang, X.X., Liu, Y.W., Wu, X.R., et al. (2015). Effects of Xie-Zhuo-Chu-Bi-Fang on miR-34a and URAT1 and their relationship in hyperuricemic mice. *J Ethnopharmacol* 161**,** 163-169. doi: 10.1016/j.jep.2014.12.001.

Tang, H., Yang, L., Li, W., Li, J., and Chen, J. (2016). Exploring the interaction between Salvia miltiorrhiza and xanthine oxidase: insights from computational analysis and experimental studies combined with enzyme channel blocking. *RSC Advances* 6(114)**,** 113527-113537. doi: 10.1039/c6ra24396g.

Tian, C., Wang, Y., Chang, H., Li, J., and La, X. (2018). Spleen-kidney supplementing formula alleviates renal fibrosis in diabetic rats via TGF-β 1-miR-21-PTEN signaling pathway. *Evidence-based Complementary and Alternative Medicine* 2018. doi: 10.1155/2018/3824357.

Tung, Y.T., Lin, L.C., Liu, Y.L., Ho, S.T., Lin, C.Y., Chuang, H.L., et al. (2015). Antioxidative phytochemicals from Rhododendron oldhamii Maxim. leaf extracts reduce serum uric acid levels in potassium oxonate-induced hyperuricemic mice. *BMC Complement Altern Med* 15**,** 423. doi: 10.1186/s12906-015-0950-7.

Wang, C.P., Wang, X., Zhang, X., Shi, Y.W., Liu, L., and Kong, L.D. (2010a). Morin improves urate excretion and kidney function through regulation of renal organic ion transporters in hyperuricemic mice. *J Pharm Pharm Sci* 13(3)**,** 411-427. doi: 10.18433/j3q30h.

Wang, C.P., Wang, Y., Wang, X., Zhang, X., Ye, J.F., Hu, L.S., et al. (2011). Mulberroside a possesses potent uricosuric and nephroprotective effects in hyperuricemic mice. *Planta Med* 77(8)**,** 786-794. doi: 10.1055/s-0030-1250599.

Wang, J., Xu, B.B., Zeng, J.X., Zhu, J.X., Wang, X.Y., Ren, G., et al. (2016a). Effect of Dendropanaxchevalieri extracts on uric acid level in hyperuricemic mice and the possible mechanism. *Chinese Journal of New Drugs* 25(3)**,** 334-338.

Wang, M.X., Liu, Y.L., Yang, Y., Zhang, D.M., and Kong, L.D. (2015). Nuciferine restores potassium oxonate-induced hyperuricemia and kidney inflammation in mice. *Eur J Pharmacol* 747**,** 59-70. doi: 10.1016/j.ejphar.2014.11.035.

Wang, Q., Yang, Q., Cao, X., Wei, Q., Firempong, C.K., Guo, M., et al. (2018). Enhanced oral bioavailability and anti-gout activity of [6]-shogaol-loaded solid lipid nanoparticles. *Int J Pharm* 550(1-2)**,** 24-34. doi: 10.1016/j.ijpharm.2018.08.028.

Wang, R., Ma, C.H., Zhou, F., and Kong, L.D. (2016b). Siwu decoction attenuates oxonate-induced hyperuricemia and kidney inflammation in mice. *Chin J Nat Med* 14(7)**,** 499-507. doi: 10.1016/s1875-5364(16)30059-0.

Wang, S., Fang, Y., Yu, X., Guo, L., Zhang, X., and Xia, D. (2019a). The flavonoid-rich fraction from rhizomes of Smilax glabra Roxb. ameliorates renal oxidative stress and inflammation in uric acid nephropathy rats through promoting uric acid excretion. *Biomed Pharmacother* 111**,** 162-168. doi: 10.1016/j.biopha.2018.12.050.

Wang, W.L., Sheu, S.Y., Huang, W.D., Chuang, Y.L., Tseng, H.C., Hwang, T.S., et al. (2016c). Phytochemicals from Tradescantia albiflora Kunth Extracts Reduce Serum Uric Acid Levels in Oxonate-induced Rats. *Pharmacogn Mag* 12(Suppl 2)**,** S223-227. doi: 10.4103/0973-1296.182171.

Wang, X., Wang, C.P., Hu, Q.H., Lv, Y.Z., Zhang, X., Ouyang, Z., et al. (2010b). The dual actions of Sanmiao wan as a hypouricemic agent: down-regulation of hepatic XOD and renal mURAT1 in hyperuricemic mice. *J Ethnopharmacol* 128(1)**,** 107-115. doi: 10.1016/j.jep.2009.12.035.

Wang, X.Y., Zhou, J.B., Shi, B., Guo, X.L., and Yan, Q.C. (2016d). Hypouricemic and nephroprotective effects of Jianpi Huashi decoction in a rat model of hyperuricemia. *International Journal of Clinical and Experimental Medicine* 9(1)**,** 455-465.

Wang, Y., Dong, L., Liu, P., Chen, Y., Jia, S., and Wang, Y. (2019b). A Randomized Controlled Trial of Chuanhutongfeng Mixture for the Treatment of Chronic Gouty Arthritis by Regulating miRNAs. *Evid Based Complement Alternat Med* 2019**,** 5917269. doi: 10.1155/2019/5917269.

Wang, Y., Lin, Z., Zhang, B., Nie, A., and Bian, M. (2017a). Cichorium intybus L. promotes intestinal uric acid excretion by modulating ABCG2 in experimental hyperuricemia. *Nutr Metab (Lond)* 14**,** 38. doi: 10.1186/s12986-017-0190-6.

Wang, Y., Lin, Z., Zhang, B., Wang, X., and Chu, M. (2019c). Chicory (Cichorium intybus L.) inhibits renal reabsorption by regulating expression of urate transporters in fructose-induced hyperuricemia. *Journal of Traditional Chinese Medical Sciences* 6(1)**,** 84-94. doi: 10.1016/j.jtcms.2019.01.001.

Wang, Y., Lin, Z.J., Nie, A.Z., Li, L.Y., and Zhang, B. (2017b). [Effect of Chinese herb chicory extract on expression of renal transporter Glut9 in rats with hyperuricemia]. *Zhongguo Zhong Yao Za Zhi* 42(5)**,** 958-963. doi: 10.19540/j.cnki.cjcmm.2017.0029.

Wang, Y., Wang, L., Li, E., Li, Y., Wang, Z., Sun, X., et al. (2014). Chuanhu anti-gout mixture versus colchicine for acute gouty arthritis: a randomized, double-blind, double-dummy, non-inferiority trial. *Int J Med Sci* 11(9)**,** 880-885. doi: 10.7150/ijms.9165.

Wang, Y., Zhao, M., Xin, Y., Liu, J., Wang, M., and Zhao, C. (2016e). (1)H NMR and MS based metabolomics study of the therapeutic effect of Cortex Fraxini on hyperuricemic rats. *J Ethnopharmacol* 185**,** 272-281. doi: 10.1016/j.jep.2016.03.043.

Wang, Y., Zhao, M., Ye, H., Shao, Y., Yu, Y., Wang, M., et al. (2017c). Comparative pharmacokinetic study of the main components of cortex fraxini after oral administration in normal and hyperuricemic rats. *Biomed Chromatogr* 31(8). doi: 10.1002/bmc.3934.

Wang, Z., Ci, X.Y., Cui, T., Wei, Z.H., Zhang, H.B., Liu, R., et al. (2019d). Effects of Chinese herb ingredients with different properties on OAT4, URAT1 and serum uric acid level in acute hyperuricemia mice. *Chinese Traditional and Herbal Drugs* 50(5)**,** 1157-1163. doi: 10.7501/j.issn.0253-2670.2019.05.020.

Wei, G.N., Su, Q.B., He, F., Zeng, X.B., Ya, Q.K., Lü, J.H., et al. (2013). Screening and chemical component analysis of anti-hyperuricemic active fraction of ethanol extract from Polyrhachis vicina Roger in hyperuricemia model mice. *Chinese Journal of Pharmacology and Toxicology* 27(4)**,** 673-677. doi: 10.3867/j.issn.1000-3002.2013.04.012.

Wei, Z., Xu, C., Liu, S., Song, F., Liu, Z., and Qu, X. (2018). Metabonomics study of the effects of traditional Chinese medicine formula Ermiaowan on hyperuricemic rats. *J Sep Sci* 41(2)**,** 560-570. doi: 10.1002/jssc.201700985.

WG, M., J, W., XW, B., HH, Z., JP, Z., XX, Z., et al. - Effects of Polygonum cuspidatum on AMPK-FOXO3alpha Signaling Pathway in Rat Model. *- Chin J Integr Med. 2019 Mar;25(3):182-189. doi: 10.1007/s11655-017-2979-6. Epub* (- 1672-0415 (Print))**,** T - ppublish.

Wu, P., Li, J., Zhang, X., Zeng, F., Liu, Y., and Sun, W. (2018a). Study of the Treatment Effects of Compound Tufuling Granules in Hyperuricemic Rats Using Serum Metabolomics. *Evid Based Complement Alternat Med* 2018**,** 3458185. doi: 10.1155/2018/3458185.

Wu, X., Liu, L., Xie, H., Liao, J., Zhou, X., Wan, J., et al. (2012a). Tanshinone IIA prevents uric acid nephropathy in rats through NF-kappaB inhibition. *Planta Med* 78(9)**,** 866-873. doi: 10.1055/s-0031-1298487.

Wu, X., Liu, L., Xie, H., Liao, J., Zhou, X., Wan, J., et al. (2012b). Tanshinone IIA prevents uric acid nephropathy in rats through NF-κB inhibition. *Planta Med* 78(9)**,** 866-873. doi: 10.1055/s-0031-1298487.

Wu, X.H., Ruan, J.L., Zhang, J., Wang, S.Q., and Zhang, Y.W. (2014a). Pallidifloside D, a saponin glycoside constituent from Smilax riparia, resist to hyperuricemia based on URAT1 and GLUT9 in hyperuricemic mice. *J Ethnopharmacol* 157**,** 201-205. doi: 10.1016/j.jep.2014.09.034.

Wu, X.H., Wang, C.Z., Wang, S.Q., Mi, C., He, Y., Zhang, J., et al. (2015). Anti-hyperuricemia effects of allopurinol are improved by Smilax riparia, a traditional Chinese herbal medicine. *J Ethnopharmacol* 162**,** 362-368. doi: 10.1016/j.jep.2015.01.012.

Wu, X.H., Wang, C.Z., Zhang, J., Wang, S.Q., Han, L., Zhang, Y.W., et al. (2014b). Effects of Smilaxchinoside A and Smilaxchinoside C, two steroidal glycosides from Smilax riparia, on hyperuricemia in a mouse model. *Phytother Res* 28(12)**,** 1822-1828. doi: 10.1002/ptr.5207.

Wu, X.H., Yu, C.H., Zhang, C.F., Anderson, S., and Zhang, Y.W. (2014c). Smilax riparia reduces hyperuricemia in mice as a potential treatment of gout. *Am J Chin Med* 42(1)**,** 257-259. doi: 10.1142/s0192415x14200018.

Wu, X.H., Zhang, J., Wang, S.Q., Yang, V.C., Anderson, S., and Zhang, Y.W. (2014d). Riparoside B and timosaponin J, two steroidal glycosides from Smilax riparia, resist to hyperuricemia based on URAT1 in hyperuricemic mice. *Phytomedicine* 21(10)**,** 1196-1201. doi: 10.1016/j.phymed.2014.03.009.

Wu, Y., He, F., Li, Y., Wang, H., Shi, L., Wan, Q., et al. (2017). Effects of Shizhifang on NLRP3 Inflammasome Activation and Renal Tubular Injury in Hyperuricemic Rats. *Evid Based Complement Alternat Med* 2017**,** 7674240. doi: 10.1155/2017/7674240.

Wu, Y., Wang, Y., Ou, J., Wan, Q., Shi, L., Li, Y., et al. (2018b). Effect and Mechanism of ShiZhiFang on Uric Acid Metabolism in Hyperuricemic Rats. *Evid Based Complement Alternat Med* 2018**,** 6821387. doi: 10.1155/2018/6821387.

Xia, N., Li, B.A., Liu, H.J., Fan, J.B., Shen, W., and He, X.G. (2017). Anti-hyperuricemic effect of Plantago depressa Willd extract in rats. *Tropical Journal of Pharmaceutical Research* 16(6)**,** 1365-1368. doi: 10.4314/tjpr.v16i6.21.

Xiang, S.W., Lai, S.C., and Meng, Y.H. (2009). [Clinical study on modified sanmiao powder in treating chronic uric acid nephropathy]. *Zhongguo Zhong Xi Yi Jie He Za Zhi* 29(11)**,** 979-981.

Xie, Z., Wu, H., Jing, X., Li, X., Li, Y., Han, Y., et al. (2017). Hypouricemic and arthritis relapse-reducing effects of compound tufuling oral-liquid in intercritical and chronic gout: A double-blind, placebo-controlled, multicenter randomized trial. *Medicine (Baltimore)* 96(11)**,** e6315. doi: 10.1097/MD.0000000000006315.

Xiong, W.W., Zhang, H.Y., Wen, L., Wang, X.Y., Zhong, G.Y., Shi, Y.F., et al. (2018). Effect of Lagotis brachystachys Maxim extract on xanthine oxidase and renal urate transporters in hyperuricemia mice. *Chinese Journal of New Drugs* 27(13)**,** 1538-1543.

Xu, K., Liu, S., Zhao, X., Zhang, X., Fu, X., Zhou, Y., et al. (2019). Treating hyperuricemia related non-alcoholic fatty liver disease in rats with resveratrol. *Biomed Pharmacother* 110**,** 844-849. doi: 10.1016/j.biopha.2018.12.039.

Xu, W.A., Yin, L., Pan, H.Y., Shi, L., Xu, L., Zhang, X., et al. (2013). Study on the correlation between constituents detected in serum from Rhizoma Smilacis Glabrae and the reduction of uric acid levels in hyperuricemia. *J Ethnopharmacol* 150(2)**,** 747-754. doi: 10.1016/j.jep.2013.09.024.

Yan, M., An, Y.T., Li, J., Wu, Z.Z., and Wang, T. (2014). [Regulatory effect of leonurus extracts on hyperuricemia in rats]. *Zhongguo Zhong Yao Za Zhi* 39(24)**,** 4856-4859.

Yang, A., Guo, H., Fu, M., and Liu, M. (2019a). Inhibitive Effects of Huashi Pill on Formation of Renal Stones by Modulating Urine Biochemical Indexes and Osteopontin in Renal Stone Rat Models. *Med Sci Monit* 25**,** 8335-8344. doi: 10.12659/msm.916247.

Yang, H.X., Su, J.X., Jiang, G.Q., and Chen, X.P. (2013). Effect of Tongxi Powder on blood uric acid of mice with acute hyperuricemia. *Chinese Traditional and Herbal Drugs* 44(12)**,** 1635-1637. doi: 10.7501/j.issn.0253-2670.2013.12.021.

Yang, L.X., Xue, J.J., Meng, X.Y., Wang, Y.S., Wu, L.L., Lv, C.Y., et al. (2019b). Effects of total flavonoids from Oxytropis falcata Bunge on the SOCS/JAK/STAT inflammatory signaling pathway in the kidneys of diabetic nephropathy model mice. *European Journal of Inflammation* 17. doi: 10.1177/2058739219861877.

Yang, T.H., Yan, D.X., Huang, X.Y., Hou, B., Ma, Y.B., Peng, H., et al. (2019c). Termipaniculatones A-F, chalcone-flavonone heterodimers from Terminthia paniculata, and their protective effects on hyperuricemia and acute gouty arthritis. *Phytochemistry* 164**,** 228-235. doi: 10.1016/j.phytochem.2019.05.019.

Yang, Y., Zhang, D.M., Liu, J.H., Hu, L.S., Xue, Q.C., Ding, X.Q., et al. (2015). Wuling San protects kidney dysfunction by inhibiting renal TLR4/MyD88 signaling and NLRP3 inflammasome activation in high fructose-induced hyperuricemic mice. *J Ethnopharmacol* 169**,** 49-59. doi: 10.1016/j.jep.2015.04.011.

Yang, Y., Zhang, L., Jiang, G., Lei, A., Yu, Q., Xie, J., et al. (2019d). Evaluation of the protective effects of Ganoderma atrum polysaccharide on acrylamide-induced injury in small intestine tissue of rats. *Food Funct* 10(9)**,** 5863-5872. doi: 10.1039/c9fo01452g.

Yao, L., Dong, W., Lu, F., and Liu, S. (2012). An improved acute gouty arthritis rat model and therapeutic effect of rhizoma Dioscoreae nipponicae on acute gouty arthritis based on the protein-chip methods. *Am J Chin Med* 40(1)**,** 121-134. doi: 10.1142/s0192415x12500103.

Yao, R., Geng, Z., Mao, X., Bao, Y., Guo, S., Bao, L., et al. (2020). Tu-Teng-Cao Extract Alleviates Monosodium Urate-Induced Acute Gouty Arthritis in Rats by Inhibiting Uric Acid and Inflammation. *Evid Based Complement Alternat Med* 2020**,** 3095624. doi: 10.1155/2020/3095624.

Yi, L.T., Li, J., Su, D.X., Dong, J.F., and Li, C.F. (2012). Hypouricemic effect of the methanol extract from Prunus mume fruit in mice. *Pharm Biol* 50(11)**,** 1423-1427. doi: 10.3109/13880209.2012.683115.

Yong, T., Chen, S., Xie, Y., Chen, D., Su, J., Shuai, O., et al. (2017). Hypouricemic Effects of Ganoderma applanatum in Hyperuricemia Mice through OAT1 and GLUT9. *Front Pharmacol* 8**,** 996. doi: 10.3389/fphar.2017.00996.

Yong, T., Zhang, M., Chen, D., Shuai, O., Chen, S., Su, J., et al. (2016). Actions of water extract from Cordyceps militaris in hyperuricemic mice induced by potassium oxonate combined with hypoxanthine. *J Ethnopharmacol* 194**,** 403-411. doi: 10.1016/j.jep.2016.10.001.

Yoon, I.S., Park, D.H., Bae, M.S., Oh, D.S., Kwon, N.H., Kim, J.E., et al. (2017a). In Vitro and In Vivo Studies on Quercus acuta Thunb. (Fagaceae) Extract: Active Constituents, Serum Uric Acid Suppression, and Xanthine Oxidase Inhibitory Activity. *Evid Based Complement Alternat Med* 2017**,** 4097195. doi: 10.1155/2017/4097195.

Yoon, I.S., Park, D.H., Ki, S.H., and Cho, S.S. (2016). Effects of extracts from Corylopsis coreana Uyeki (Hamamelidaceae) flos on xanthine oxidase activity and hyperuricemia. *J Pharm Pharmacol* 68(12)**,** 1597-1603. doi: 10.1111/jphp.12626.

Yoon, I.S., Park, D.H., Kim, J.E., Yoo, J.C., Bae, M.S., Oh, D.S., et al. (2017b). Identification of the biologically active constituents of Camellia japonica leaf and anti-hyperuricemic effect in vitro and in vivo. *Int J Mol Med* 39(6)**,** 1613-1620. doi: 10.3892/ijmm.2017.2973.

You, W., Wang, J., Zou, Y., Che, K., Hou, X., Fei, H., et al. (2019). Modified Chuanhu anti-gout mixture, a traditional Chinese medicine, protects against potassium oxonate-induced hyperuricemia and renal dysfunction in mice. *J Int Med Res* 47(5)**,** 1927-1935. doi: 10.1177/0300060519831182.

Yu, X.N., Wu, H.Y., Deng, Y.P., Zhuang, G.T., Tan, B.H., Huang, Y.Z., et al. (2018). "Yellow-dragon Wonderful-seed Formula" for hyperuricemia in gout patients with dampness-heat pouring downward pattern: a pilot randomized controlled trial. *Trials* 19(1)**,** 551. doi: 10.1186/s13063-018-2917-8.

Yuan, X., Fan, Y.S., Xu, L., Xie, G.Q., Feng, X.H., and Qian, K. (2019). Jia-Wei-Si-Miao-Wan alleviates acute gouty arthritis by targeting NLRP3 inflammasome. *J Biol Regul Homeost Agents* 33(1)**,** 63-71.

Yuk, H.J., Lee, Y.S., Ryu, H.W., Kim, S.H., and Kim, D.S. (2018). Effects of Toona sinensis Leaf Extract and Its Chemical Constituents on Xanthine Oxidase Activity and Serum Uric Acid Levels in Potassium Oxonate-Induced Hyperuricemic Rats. *Molecules* 23(12). doi: 10.3390/molecules23123254.

Zeng, J.X., Xu, B.B., Li, M., Li, X.W., Zhu, J.X., Wang, X.Y., et al. (2015). Effect of Lagotis brevituba Maxim. extract in reducing uric acid level in hyperuricemia mice and it's mechanism. *Chinese Journal of New Drugs* 24(21)**,** 2489-2493.

Zhang, H.J., Li, L.N., Zhou, J., Yang, Q.Q., Liu, P.G., Xu, P., et al. (2017a). Effects of Gnaphalium affine D. Don on hyperuricemia and acute gouty arthritis. *J Ethnopharmacol* 203**,** 304-311. doi: 10.1016/j.jep.2017.03.057.

Zhang, M., Wang, J.F., and Wang, Y.F. (2009). [Effects of serial gout granules on insulin-resistance in primary gout patients]. *Zhongguo Zhong Xi Yi Jie He Za Zhi* 29(12)**,** 1068-1072.

Zhang, R., Zhan, S., Li, S., Zhu, Z., He, J., Lorenzo, J.M., et al. (2018a). Anti-hyperuricemic and nephroprotective effects of extracts from Chaenomeles sinensis (Thouin) Koehne in hyperuricemic mice. *Food Funct* 9(11)**,** 5778-5790. doi: 10.1039/c8fo01480a.

Zhang, S., Zhuang, J., Yue, G., Wang, Y., Liu, M., Zhang, B., et al. (2017b). Lipidomics to investigate the pharmacologic mechanisms of ginkgo folium in the hyperuricemic rat model. *J Chromatogr B Analyt Technol Biomed Life Sci* 1060**,** 407-415. doi: 10.1016/j.jchromb.2017.06.037.

Zhang, W., Du, W., Li, G., Zhang, C., Yang, W., Yang, S., et al. (2019a). Constituents and Anti-Hyperuricemia Mechanism of Traditional Chinese Herbal Formulae Erding Granule. *Molecules* 24(18). doi: 10.3390/molecules24183248.

Zhang, X.X., Sun, W.F., and Xu, W. (2011). [Assessment on the clinical efficacy and safety of xiezhuo chubi recipe in treating hyperuricemia]. *Zhongguo Zhong Xi Yi Jie He Za Zhi* 31(9)**,** 1216-1219.

Zhang, X.Y., Cheng, J., Zhao, P., Chen, K.L., and Li, J. (2019b). Screening the Best Compatibility of Selaginella moellendorffii Prescription on Hyperuricemia and Gouty Arthritis and Its Mechanism. *Evid Based Complement Alternat Med* 2019**,** 7263034. doi: 10.1155/2019/7263034.

Zhang, Y., Jin, L., Liu, J., Wang, W., Yu, H., Li, J., et al. (2018b). Effect and mechanism of dioscin from Dioscorea spongiosa on uric acid excretion in animal model of hyperuricemia. *J Ethnopharmacol* 214**,** 29-36. doi: 10.1016/j.jep.2017.12.004.

Zhang, Z.C., Su, G.H., Luo, C.L., Pang, Y.L., Wang, L., Li, X., et al. (2015). Effects of anthocyanins from purple sweet potato (Ipomoea batatas L. cultivar Eshu No. 8) on the serum uric acid level and xanthine oxidase activity in hyperuricemic mice. *Food Funct* 6(9)**,** 3045-3055. doi: 10.1039/c5fo00499c.

Zhang, Z.C., Zhou, Q., Yang, Y., Wang, Y., and Zhang, J.L. (2019c). Highly Acylated Anthocyanins from Purple Sweet Potato ( Ipomoea batatas L.) Alleviate Hyperuricemia and Kidney Inflammation in Hyperuricemic Mice: Possible Attenuation Effects on Allopurinol. *J Agric Food Chem* 67(22)**,** 6202-6211. doi: 10.1021/acs.jafc.9b01810.

Zhao, P., Chen, K.L., Zhang, G.L., Deng, G.R., and Li, J. (2017). Pharmacological Basis for Use of Selaginella moellendorffii in Gouty Arthritis: Antihyperuricemic, Anti-Inflammatory, and Xanthine Oxidase Inhibition. *Evid Based Complement Alternat Med* 2017**,** 2103254. doi: 10.1155/2017/2103254.

Zhou, M., Wang, Y.F., Zhou, R., Zhang, M., and Li, B. (2013). [Treatment of gouty arthritis in different phases by a series of tongfeng granule: an efficacy observation]. *Zhongguo Zhong Xi Yi Jie He Za Zhi* 33(12)**,** 1603-1607.

Zhou, Q., Lin, F.F., Liu, S.M., and Sui, X.F. (2017). Influence of the total saponin fraction from Dioscorea nipponica Makino on TLR2/4-IL1R receptor singnal pathway in rats of gouty arthritis. *J Ethnopharmacol* 206**,** 274-282. doi: 10.1016/j.jep.2017.04.024.

Zhou, Q., Yu, D.H., Liu, S.M., and Liu, Y. (2015). Total saponins from Discorea nipponica makino ameliorate urate excretion in hyperuricemic rats. *Pharmacogn Mag* 11(43)**,** 567-573. doi: 10.4103/0973-1296.160442.

Zhou, Q., Yu, D.H., Zhang, C., Liu, S.M., and Lu, F. (2014). Total saponins from Discorea nipponica ameliorate urate excretion in hyperuricemic mice. *Planta Med* 80(15)**,** 1259-1268. doi: 10.1055/s-0034-1383048.

Zhou, Y., Zhang, X., Li, C., Yuan, X., Han, L., Li, Z., et al. (2018). Research on the pharmacodynamics and mechanism of Fraxini Cortex on hyperuricemia based on the regulation of URAT1 and GLUT9. *Biomed Pharmacother* 106**,** 434-442. doi: 10.1016/j.biopha.2018.06.163.

Zhu, C., Xu, Y., Liu, Z.H., Wan, X.C., Li, D.X., and Tai, L.L. (2018). The anti-hyperuricemic effect of epigallocatechin-3-gallate (EGCG) on hyperuricemic mice. *Biomed Pharmacother* 97**,** 168-173. doi: 10.1016/j.biopha.2017.10.013.

Zhu, F., Yin, L., Ji, L., Yang, F., Zhang, G., Shi, L., et al. (2016). Suppressive effect of Sanmiao formula on experimental gouty arthritis by inhibiting cartilage matrix degradation: An in vivo and in vitro study. *Int Immunopharmacol* 30**,** 36-42. doi: 10.1016/j.intimp.2015.11.010.

Zhu, L., Dong, Y., Na, S., Han, R., Wei, C., and Chen, G. (2017). Saponins extracted from Dioscorea collettii rhizomes regulate the expression of urate transporters in chronic hyperuricemia rats. *Biomed Pharmacother* 93**,** 88-94. doi: 10.1016/j.biopha.2017.06.022.

Zhu, W., Pang, M., Dong, L., Huang, X., Wang, S., and Zhou, L. (2012). Anti-inflammatory and immunomodulatory effects of iridoid glycosides from Paederia scandens (LOUR.) MERRILL (Rubiaceae) on uric acid nephropathy rats. *Life Sci* 91(11-12)**,** 369-376. doi: 10.1016/j.lfs.2012.08.013.

Zuo, J., He, H., Zuo, Z., Bou-Chacra, N., and Lobenberg, R. (2018). Erding Formula in hyperuricaemia treatment: unfolding traditional Chinese herbal compatibility using modern pharmaceutical approaches. *J Pharm Pharmacol* 70(1)**,** 124-132. doi: 10.1111/jphp.12840.

Zuo, J., Zhang, W., Jian, H., Bou-Chacra, N., and Löbenberg, R. (2020). Esculetin as bioactive marker: Towards a rational scientific approach for the treatment of hyperuricemia using traditional chinese medicine. *Brazilian Journal of Pharmaceutical Sciences* 56. doi: 10.1590/s2175-97902019000417827.
